# An Adaptable, Open-Access Test Battery to Study the Fractionation of Executive-Functions in Diverse Populations

**DOI:** 10.3389/fpsyg.2021.627219

**Published:** 2021-03-30

**Authors:** Gislaine A. V. Zanini, Monica C. Miranda, Hugo Cogo-Moreira, Ali Nouri, Alberto L. Fernández, Sabine Pompéia

**Affiliations:** ^1^Departamento de Psicobiologia, Universidade Federal de São Paulo, São Paulo, Brazil; ^2^Programa de Pós Graduação em Psicologia-Psicossomática, Universidade Ibirapuera, São Paulo, Brazil; ^3^School of Public Health, Li Ka Shing Faculty of Medicine, The University of Hong Kong, Pokfulam, China; ^4^Programa de Pós Graduação em Psiquiatria, Universidade Federal de São Paulo, São Paulo, Brazil; ^5^Department of Education Studies, Faculty of Humanities, Malayer University, Malayer, Iran; ^6^Universidad Católica de Córdoba, Universidad Nacional de Córdoba, Córdoba, Argentina

**Keywords:** executive functions, human development, socioeconomic factors, inhibition, shifting, updating, attention control, adolescent

## Abstract

The umbrella-term ‘executive functions’ (EF) includes various domain-general, goal-directed cognitive abilities responsible for behavioral self-regulation. The influential unity and diversity model of EF posits the existence of three correlated yet separable executive domains: inhibition, shifting and updating. These domains may be influenced by factors such as socioeconomic status (SES) and culture, possibly due to the way EF tasks are devised and to biased choice of stimuli, focusing on first-world testees. Here, we propose a FREE (Free Research Executive Function Evaluation) test battery that includes two open-access tasks for each of the three abovementioned executive domains to allow latent variables to be obtained. The tasks were selected from those that have been shown to be representative of each domain, that are not copyrighted and do not require special hardware/software to be administered. These tasks were adapted for use in populations with varying SES/schooling levels by simplifying tasks/instructions and using easily recognized stimuli such as pictures. Items are answered verbally and tasks are self-paced to minimize interference from individual differences in psychomotor and perceptual speed, to better isolate executive from other cognitive abilities. We tested these tasks on 146 early adolescents (aged 9–15 years) of both sexes and varying SES, because this is the age group in which the executive domains of interest become distinguishable and in order to confirm that SES effects were minimized. Performance was determined by Rate Correct Scores (correct answers divided by total time taken to complete blocks/trial), which consider speed-accuracy trade-offs. Scores were sensitive to the expected improvement in performance with age and rarely/inconsistently affected by sex and SES, as expected, with no floor or ceiling effects, or skewed distribution, thus suggesting their adequacy for diverse populations in these respects. Using structural equation modeling, evidence based on internal structure was obtained by replicating the three correlated-factor solution proposed by the authors of the model. We conclude that the FREE test battery, which is open access and described in detail, holds promise as a tool for research that can be adapted for a wide range of populations, as well as altered and/or complemented in coming studies.

## Introduction

The term ‘executive functions’ (EF) encompasses several domain-general cognitive abilities that govern self-regulation of thought and behavior (Baggetta and Alexander, 2016; Friedman and Miyake, 2017). Abilities that are regarded as EFs include logical reasoning, planning, cognitive flexibility, and inhibiting automatic behaviors, and usually involve prefrontal cortex activation (Baggetta and Alexander, 2016; Friedman and Miyake, 2017). Crucially, EFs are goal-directed processes (Baggetta and Alexander, 2016) acting upon information held in working memory at any given moment and are closely related to the concept of controlled attention (Friedman and Miyake, 2017). Despite the short-term nature of these processes, executive abilities predict long-term outcomes such as mental and physical health, socioeconomic status (SES), academic and professional achievements, addictive profile, criminal behavior and many other individual characteristics in later life (see Moffitt et al., 2011). Hence their importance for public policy and their being targeted for investigation across a wide range of areas such as cognitive psychology, education, neuropsychology and social psychology.

The most influential EF model (Baggetta and Alexander, 2016) is the unity and diversity model proposed initially by Miyake et al. (2000). This model posits three independent but correlated executive domains: (1) inhibition of prepotent/automatic responses, the ability to override automatic behaviors (henceforward called “inhibition” unless specified otherwise); (2) shifting or switching, the ability to alternate between different tasks; and (3) updating content in working memory so as to retain only information that is relevant for a given goal.

Structural equation modeling shows that these three executive domains are separable (executive “diversity”) at the level of latent variables but are also intercorrelated (“unity”), a pattern of effects that has been replicated in many studies across populations of different ages and characteristics (Friedman and Miyake, 2017; Karr et al., 2018). Latent variables do not correspond directly to participants’ performance (raw scores) on executive tasks but are inferred from mathematical models that determine common elements (underlying factors) for performance on different tasks. If there is theoretical evidence that two or more tasks measure the same construct, a latent variable obtained from them will indicate their shared cognitive processes while eliminating other abilities that may also contribute to performance but do not reflect executive functioning *per se* (task impurity). For example, two tasks may involve executive shifting but one might require color perception while another might demand perceiving sounds. A latent variable obtained from performance in these tests should reflect the shared shifting component while excluding other task-specific perceptual, motor and cognitive abilities. Using more than one test to assess different cognitive constructs is also a means of mitigating the problem of determining reliability, which is generally low in EF measures since they rely on novelty and require use of strategies and problem solving that can decrease once tasks become familiar (see Sherman et al., 2011; Friedman and Miyake, 2017). Therefore, both task impurity and low reliability, which is seldom reported in executive function studies in adolescents (Nyongesa et al., 2019), are reduced by using latent factors (see Friedman and Miyake, 2017). The use of this approach has shown that separable EF domains reflect activity of different brain structures, systems and connectivity, are highly heritable and predict real-world behavior beyond measures of intelligence, to which, however, they are correlated (Friedman and Miyake, 2017).

A recent meta-analysis (Karr et al., 2018) found that this three-factor model, and also an alternative, “nested”^1^ solution, are the ones that are most commonly replicated in the literature on adolescents and adults. Karr et al. (2018) pointed out, however, that many models had inadequate fit indices. This can be due to lack of uniformity among studies regarding choice of tasks to compose the latent factors. Indeed, many studies have suggested that updating may be equated to working memory or working memory capacity and that shifting is the same as cognitive flexibility (e.g., Diamond, 2013). Additionally, there seems to be many types of executive inhibition (Friedman and Miyake, 2004) although the ‘unity and diversity’ model explores only one of them (inhibition of prepotent/automatic responses). Considering these abilities as equivalent is not strictly accurate (Morra et al., 2018), a discussion that goes beyond the scope of the present study, which used the conceptualization of executive domains strictly following Miyake et al. (2000). To use this framework, we consider that tasks that assess EF must be chosen from among those that have already been shown to be representative of each domain as per the theoretical proposal under investigation. Furthermore, it is unfortunate that most published works that did adopt this approach are difficult to replicate because they seldom provide sufficient detail on the characteristics of the tasks, instructions, stimuli and scoring methods.

The open-access test battery proposed here consists mostly of tasks adapted from those used by proponents of the model to assess the unity and diversity of the three-correlated factor model in different sociocultural contexts. To explain why this is important we begin with an overview of limitations of currently available EF test batteries. We then discuss data on the impact of diversity on EF performance and describe factors that affect these cognitive abilities that can bias EF measurements and that were taken into account in developing our test battery. Next, we explain why we assessed this test battery’s adequacy with a sample of early adolescents from a developing country and how tasks were selected and adapted for this population, including detailed explanations to enable replication.

Currently available EF test batteries include: the National Institute of Health Examiner (NIH Examiner: Kramer et al., 2014); the Cambridge Neuropsychological Test Automated Battery (CANTAB: Cambridge Cognition, 1996); the Developmental Neuropsychological Assessment (NEPSY: Korkman et al., 1998); the Neuropsychological Battery of Executive Functions and Frontal Lobes (Lázaro et al., 2012), and the Delis-Kaplan Executive Function System (D-KEFS: Delis et al., 2001). None of these were built to test the EF unity and diversity as conceptualized by proponents of the model. Also, few have been adapted for use in languages other than English and stimuli were selected for testees from developed countries and may be inadequate in other socioeconomic and cultural contexts. Consequently, their psychometric properties have seldom been determined in societies that differ from the original population for which they were built (Nyongesa et al., 2019). Furthermore, access is restricted because a fee must be paid. To improve knowledge on EF worldwide it is therefore crucial to develop measures that include tasks, instructions and stimuli that may be adapted for different types of samples and that do not have restricted access, which limits the possibility of replication in different cultural and socioeconomic environments in which research funding is limited. After all, compared to the developed countries, less privileged nations have larger populations in whom EF are more severely affected by factors such as low SES and inadequate schooling, as discussed below.

Another point concerning the abovementioned test batteries is that testees respond by pressing buttons or keys on keyboards (e.g., Huizinga et al., 2006; Prencipe et al., 2011). Although this type of response is convenient for experimenters in terms of automatized scoring, it has many shortcomings. Key pressing is affected by individual variation in psychomotor speed and speed of information processing, which may affect performance in higher-order cognitive processing tasks (Schubert et al., 2019) such as EF and varies across cultures (see Henrich et al., 2010; Kelkar et al., 2013). Additionally, response selection by key press, such as between-hand choice reaction times, which are commonly used in the literature on executive functions, is difficult to implement. This type of response seems not to require brain activation that is directly related to the stimuli themselves, but rather to another type of cognitive process, a stimulus-response association that relies on stimulus-to-response mapping (see Vidal et al., 2015). The latter go beyond the type of executive process that should be tapped to better understand EF fractionation because they introduce motor errors that are not executive errors *per se* (Vidal et al., 2015). Furthermore, most executive tasks that are answered by pressing keys require answers that are of verbal nature (i.e., indicating a color or shape of a stimulus by pressing a corresponding key). To do so, declarative content is first encoded (e.g., “red”) and then reformatted into an action-oriented procedural representation (“press key r”) with significant cognitive costs of maintaining instructions in a declarative format until it is transformed into an action (see Formica et al., 2020). This type of translation is more automatic when there is a dimensional overlap between stimuli and response (e.g., vocal responses to verbal stimuli), thus shortening reaction times (Wang and Proctor, 1996). In this way, performance is less contaminated with “translations” from responses to actions that are not automatized in real life, thus reducing executive task impurity.

All these types of psychomotor biases may be minimized by using vocal responses executed by automatized motor programs (see Vidal et al., 2015) once people have learned to speak in early childhood. Although this has disadvantages for experimenters when it comes to scoring, this response mode allows better differentiation of executive from psychomotor responses. We therefore decided to use vocal responses in our test battery, i.e., naming characteristics of stimuli which represent common objects, numbers, shapes, colors and semantic categories that were selected using criteria from published reports on types of stimuli that are adequate for people from varying sociocultural and educational backgrounds (Izard et al., 2009; Rasmussen and Bisanz, 2011; Fernández and Abe, 2018). The above-mentioned confounding psychomotor effects may be even greater when executive tasks involve time-limited exposure to stimuli, as do many EF tasks. This can distort findings in studies with confirmatory factor models (Schweizer et al., 2019) such as those used when studying EF unity and diversity. This happens because participants can fail to answer in the allotted time in different proportions and so differ in terms of the subset of items that are used in the analyses. Establishing stimulus exposure time can also be difficult when testing samples that vary in terms of socio-cultural factors and age. A possible solution is enabling testees to control stimuli presentation and response time by using self-paced tasks (e.g., McMillan et al., 2007; Lawlor-Savage and Goghari, 2016), as proposed here.

Another factor that should be considered when devising executive tasks is that most of them require testees to work as fast as possible while avoiding errors and then measuring performance either by the number of correct responses or the time testees take to respond to each stimulus or block of stimuli. This approach does not recognize (Hedge et al., 2018) that more errors are committed if tasks are done quickly, while accuracy may be increased by completing tasks them at a slower pace. Because of this speed-accuracy trade-off, analyzing speed and accuracy separately may lead to contradictory findings (see Vandierendonck, 2017). To avoid this and maximize the chances of detecting the effects of interest, we combined these metrics, integrating speed and accuracy aspects of performance using the Rate Correct Score (RCS): number of correct responses divided by total time taken to finish each task (see Vandierendonck, 2017). This yields scores that show the number of correct responses per unit of time (seconds). Lower scores mean that less correct responses are given per second, or that the task is more difficult than when RCS values are higher. According to Vandierendonck (2017), the RCS score accounts for a larger proportion of variance than speed and accuracy scores individually. This is true in cases in which speedy responses lead to more errors, and also when only speed or accuracy are affected. However, RCS may have skewed distributions, so this aspect must be checked as it may adversely affect many types of statistical analysis and increase sensitivity to outliers in small samples (Vandierendonck, 2017). Additionally, all tasks must be completed through to the end, with a no-discontinuation rule to avoid psychometric distortions (von Davier et al., 2019).

In addition to these limitations regarding the use of psychomotor responses and speed v. accuracy trade-offs, other important factors that may mask EF task performance include culture and SES of samples, their developmental trajectories and sex differences in cognitive abilities, as discussed next.

A widely cited study claims that samples from Western, Educated, Industrialized, Rich, and Democratic (WEIRD) populations are the least representative of human behavior worldwide (Henrich et al., 2010; Rad et al., 2018). Henrich et al. (2010) found that most psychological processes show cross-cultural variation beyond aspects of social cognition and moral judgment which would be expected to vary. Culture, in the broad sense of the word, includes country/nation of origin, social groups, levels of income, customs, neighborhoods, etc., which seems to regulate how people perceive, explain and respond to various phenomena (Triandis, 1996), impacting the way they process information and make executive decisions (Masuda and Nisbett, 2001; Duffy and Kitayama, 2007; Henrich et al., 2010; Kelkar et al., 2013). Cultural differences therefore pose challenges in cognitive assessment; they incorporate bias that hinders the comparability of data from samples of different cultures/contexts (Norman et al., 2011; Fernández and Abe, 2018; Foulkes and Blakemore, 2018).

Cultural differences may, in part, reflect differences across or within countries in terms of SES. Low SES and the stressful everyday life events with which it is associated (Zhang et al., 2019) directly impact brain development, including alterations in areas such as the prefrontal cortex (Johnson et al., 2016; Foulkes and Blakemore, 2018) and, therefore, executive functioning, through various still unclear biological mechanisms (Johnson et al., 2016; Haft and Hoeft, 2017). Together, these factors result in lower SES individuals tending to score worse on executive measures, within and between countries, but not necessarily both (see Howard et al., 2019).

Socioeconomic status encompasses many material and non-material factors such as education, income, job prestige and neighborhood (Farah, 2017). Ideally, many of these characteristics should be considered jointly (Farah, 2017) in studies which explore its effects on cognition, such as testees’ and parental schooling, living conditions and family purchasing power (see Sirin, 2005; Sullivan et al., 2016; Farah, 2017). These SES effects reach higher effect sizes when multiple executive function measures are used (Lawson et al., 2018). The reason for this remains unknown. Although multiple measures of the same domains may reduce measurement error (Lawson et al., 2018), they might also increase the probability of finding SES effects due to characteristics of tasks/stimuli that are not executive in nature and that benefit performance in better cognitively stimulated or schooled individuals. For example, people who have had inadequate schooling might score lower in tasks that involve manipulating items that represent low frequency words not because their executive abilities are impaired but because they do not have adequate representations of the concepts that they are supposed to manipulate. Therefore, EF of individuals from different SES, cultures or contexts should only be compared when there is evidence that SES/cultural/context-related differential test requirements are strictly executive in nature.

Age also plays an important role in EF performance, which emerges in preschoolers and continues to develop until adulthood, along with brain maturation (Anderson et al., 2001; Galvan et al., 2006; Huizinga et al., 2006; Steinberg et al., 2009; McAuley and White, 2011; Prencipe et al., 2011; Foulkes and Blakemore, 2018). Importantly, it is in early adolescence that the executive domains of interest here become dissociable (Karr et al., 2018), possibly due to major age- and pubertally induced brain changes (e.g., Goddings et al., 2019). The developmental trajectories of these domains are also variable (Reimers and Maylor, 2005; Huizinga et al., 2006; Best and Miller, 2010; Magar et al., 2010; Tamnes et al., 2010; McAuley and White, 2011). Relations between executive domains also change with age although there is no consensus on how they do so (see review by Best and Miller, 2010; Karr et al., 2018). An importance issue is that most studies in this field used different statistical models and/or tasks that do not tap the executive abilities conceptualized by Miyake et al. (2000). Although several studies report EF domains as becoming separable during early adolescence (Latzman and Markon, 2010; Wu et al., 2011; Li et al., 2015), adding (e.g., Hartung et al., 2020) or using less domains (e.g., St Clair-Thompson and Gathercole, 2006; van der Sluis et al., 2007; Xu et al., 2013) changes model structure, as well as the pattern of intercorrelations across latent factors. In a similar vein, many studies of this population have misunderstood the domains (see Morra et al., 2018), by using maintenance of information in working memory/working memory capacity as a proxy for updating, and cognitive flexibility in place of shifting (e.g., Lehto et al., 2003; Latzman and Markon, 2010; McAuley and White, 2011; Rose et al., 2011; Arán-Filippetti, 2013; Poon, 2018; Li et al., 2019; Theodoraki et al., 2019). Work that selected tasks based on Miyake et al.’s (2000) study were fewer in number (e.g., Huizinga et al., 2006; van der Sluis et al., 2007; Lee et al., 2013; Xu et al., 2013; Nyongesa et al., 2019) and only some were conducted in non-WEIRD countries (Duan et al., 2010; Wu et al., 2011; and Xu et al., 2013, all in China; see also are Nyongesa et al., 2019). In the present scenario, it is noteworthy that culture of origin or SES were basically ignored as factors that could have played a part in the variability across results from different studies. Nevertheless, executive domains seem to become more dissociable as adolescents age, irrespective of this omission (e.g., Wu et al., 2011; Lee et al., 2013; Xu et al., 2013).

Other characteristics of tasks in previous studies of under-aged participants could also have confounded developmental effects. Because reaction times decrease from early childhood to mid-adolescence, when they reach asymptotic values (Kail, 1991; Fry and Hale, 2000; Span et al., 2004; Rose et al., 2011), if stimuli are available for only a short fixed rate of time, younger or slower testees may be prevented from processing and responding to some or many trials. Assuming these are executive errors or treating them as missing data distorts findings as discussed above. Furthermore, using tasks that required key presses and speedy responses may lead to executive function measurements that are highly contaminated by psychomotor abilities, which might explain the intercorrelation between executive and motor abilities in children and adolescents (Rigoli et al., 2012; Ludyga et al., 2019). In contrast, once speaking is automatized, vocal responses facilitate comparisons of testees of different ages, since time of lexical access when naming pictures in conditions with presence of distractors, for instance, has been found to be very similar in children, adolescents and adults (Jerger et al., 2002). Naming is, however, dependent on word knowledge, lexical access and search mechanisms that improve until adulthood and decline in the elderly (Kavé et al., 2010). Hence care must be taken to choose word names that are well known to participants.

There are also different sex-related developmental trajectories in motor development (Thomas and French, 1985; Quatman-Yates et al., 2012; Katic et al., 2013) and other abilities that are not executive in nature, but contribute to performance in executive tasks: males usually outperform females on tasks involving spatial cognition, while the opposite is true for tasks involving verbal processing, but these effects are usually minimal (e.g., Miller and Halpern, 2014). Some other cognitive abilities, such as naming colors and objects, may also differ between sexes in some populations/cultures, but not in others (Wolff et al., 1983). Nonetheless, there seems to be no clear pattern of effects of sex differences in EFs as such, although this has seldom been investigated; when sex differences are assessed they are usually absent or subtle and inconsistent (for examples in adolescents, see: Tamnes et al., 2010; Xu et al., 2013).

In sum, the lack of uniform EF unity and diversity models in adults and adolescents may stem from the fact that very few studies in this area: (1) assumed that culture and SES interfere in performance and controlled for these effects; (2) selected representative tasks from each domain, so may have inadvertently assessed other cognitive/executive abilities; and (3) failed to account for the effects on executive performance of psychomotor speed, which varies across individuals, ages, sexes, culture and SES. In light of these issues, it seems opportune to propose a test battery that addresses these limitations, which was undertaken here.

### The Present Study

This study describes the process of development of a test battery that considers the unity and diversity model of EF as it was firstly conceptualized. The battery was proposed as being adaptable considering diversity within or between populations, including metrics that minimize biases of psychomotor abilities. In the spirit of contributing to the open science movement we named our battery FREE (Free Research Executive Evaluation).

To ensured that the test battery showed content validity (see Sherman et al., 2011): (1) tests were selected based on a theoretical model (Miyake et al., 2000); (2) there are literature reviews to support the model (e.g., Friedman and Miyake, 2017; Karr et al., 2018); and (3) the studied constructs and their operationalization/scoring (with RCS) were clearly defined. Details on the tasks, number of trials and types of stimuli can be found in the “Materials and Methods” section and the Supplementary Material I.

Having developed the test battery we reasoned that it would be necessary to explore some of its psychometric properties. Our first attempt to do so, described herein, involved a Brazilian sample of early adolescents who were from different SES backgrounds. It is in this phase of life that the three executive domains of interest seem to become separable (Karr et al., 2018). Hence, if the test battery were to capture the distinguishable nature of the executive domains, this would be a good indication that it holds promise and can be explored in other studies, including those in adolescence, a key phase of life for executive development.

Although Brazil is regarded as an upper-middle-income country (World Bank, 2019), it has a large poverty-stricken and under- or inadequately schooled population (OECD, 2017), so the EF effects from a wide range of SES could be investigated. Obtaining evidence that the scores on each task were sensitive to expected developmental (age) and other demographic factors (SES and sex) served as criterion-related evidence of validity (Sherman et al., 2011). Also, we aimed to obtain evidence based on internal structure by trying to replicate the three correlated factor solution of the EF unity and diversity model found in North American adults via structural equation modeling.

Our objectives were to show: (1) no clear ceiling or floor effects on performance of tasks because they were designed and piloted to be adequate for testees of different ages and levels of executive proficiency; (2) the expected developmental improvement in performance with age in all tasks, but possibly less improvement in inhibition because some studies have found this ability to mature later (see Discussion); (3) either an inconsistent pattern of SES findings (effects not found in both the tasks in each domain) or effects of low effects sizes, because stimuli were selected to be highly familiar and easy to recognize, even by those with low SES and/or inadequate schooling; (4) inconsistent or low effect sizes for sex effects as these are rarely found in the literature; (5) correlation between performance in the executive tasks; and (6) an indication that the tasks are able to pick up the *separability* of the executive domains, which was tested with a three factor (see text footnote 1) confirmatory structural equation model solution, with correlated yet separable executive domains at the level of latent variables following Miyake et al.’s (2000) model. In this phase of our work, we focused on the description of the test battery and did not intend to find the best factor solution for our sample, nor test alternative models to that of Miyake et al. (2000), on which the tasks were based.

## Materials and Methods

### Participants

We tested a convenience sample of 146 (80 girls) 9- to 15-year-old children/adolescents of varying SES, drawn from public and private schools from a megalopolis in Brazil, the City of São Paulo. Participants were enrolled in the local equivalent of the United States grades 4 through 9. They had normal or corrected vision, were native Portuguese-speakers and regarded by legal guardians as typically developing based on a detailed health questionnaire. Exclusion criteria were having been held back in school for a year or more and being a student with special needs, which would have characterized them as having clinical or cognitive limitations. Those who were on daily medication were also excluded due to possible presence of chronic clinical disorders that could affect cognition and/or use of medication that could affect executive, perceptual and motor abilities.

### Procedures

This study has approved by the local Ethics Committee (CAAE # 56284216.7.0000.5505 and 50662015.3.0000.5505). The sample size was chosen to be similar to that of Miyake et al.’s (2000) study (*N* = 137), in which the three-factor model of EF unity and diversity was proposed.

Firstly, we searched the literature to select tasks that fulfilled our criteria, as detailed below. We then adapted the tasks, stimuli and instructions to make them as familiar and simple as possible to try to minimize cultural and SES effects, which in all cases involved consulting panels of experts in cognitive psychology (data not shown). Next, we piloted the tasks on people of different ages and SES to ensure that the instructions were clear, and that ceiling and floor effects were avoided (data not shown). We then compiled an administration and correction manual and piloted it for three rounds (data not shown) including, respectively, 17, 17, and 10 health professionals with no or minimum experience in neuropsychological testing. This was done to evaluate their ability to administer and correct the tasks only using the manual, *without any input* from the experimenters who proposed the FREE. Based on these pilot studies, the manual was altered to enhance clarity and the revised version was them reviewed by 10 neuropsychologists with more than 9 years of clinical experience, who made minor suggestions. Only the final version of the manual is appended in Supplementary Material II (in English) and III (in Portuguese).

The team of examiners was then trained by the experimenters to administer and correct the tasks based on the Portuguese version of the test manual. Testing did not start until they had acquired familiarity with the procedures. Schools were then contacted and those interested in taking part in the study allowed us to approach the students and legal guardians, who were shown 4-min videos describing the study. Having obtained informed assent and guardian consent, the latter were asked to provide medical history and demographic details by filling in questionnaires including SES metrics and other behavioral data that will not be discussed here.

The participants were tested at their schools in individual sessions. Executive tasks were administered using touchscreen tablets in one of four pseudorandom orders that alternated executive tasks with behavioral questionnaires that will be described elsewhere. The order of tasks for each participant was randomized by shuffling four orders and placing them on a list. As the experiment progressed, each examiner picked a test order from the list that corresponded to each testees’ successive number. After reading or being read the instructions (testee’s choice) for each task, testees briefly practiced with some stimuli to ensure they understood the tasks (except for inhibition tasks, for which there were no practice trials, following the literature: Strauss et al., 2006). If participants understood the instructions, they went on to the task. If not, instructions were explained again until testees managed to perform the practice trials correctly or reported having understood what they were supposed to do. Inter-rater reliability was estimated in around 10% of the sample. The measures reported here took around 30 min test completion time. Breaks were offered and taken if testees asked for them. Participants were reimbursed for their transportation expenses and provided with a “science partner” certificate.

### Measures

#### FREE (Free Research Executive Evaluation) Test Battery

##### Criteria for the selection of tasks, stimuli and general task characteristics

We searched the literature to find non-copyrighted executive function measures that did not require complex equipment or software to measure reaction time for each stimulus so that they would be accessible for a wide range of poorly funded experimenters worldwide. Specifically, the tasks were selected from published EF studies that involved confirmatory factor analyses to examine fractionation of these cognitive functions into three domains as conceptualized by Miyake et al. (2000): inhibition, shifting and updating. To determine latent variables, we chose two tasks that tap each of these three executive abilities, both of which displayed good factor loading in their domains in models with adequate fit indices. The only exception^2^ was the Happy Sad Stroop task (adapted from Lagattuta et al., 2011; Kramer et al., 2015). The selected tasks are described in the Material section and detailed in the Supplementary Material I.

Testing material included a pen/pencil, a stopwatch (for the experimenter) and a touch screen tablet^3^ on which the tasks were presented using PDF files, which can be read by many open source software. To reduce cognitive overloading and test anxiety, instructions were kept to a minimum, included sentences such as “when you forget…” instead of “if you forget” and the executive tasks were named “activities” and not “tests.”

The slides had white background for more contrast with written instructions and stimuli. Instructions were printed in black ink in sans serif (see Vandendorpe, 2013) font Calibri 24 for easy on-screen reading. Criteria used to select stimuli required them to be adaptable to distinct cultural/SES contexts in which potential participants have at least some level of familiarity with written symbols such as numbers, and basic reading proficiency. All stimuli were visual, which purportedly reduce possible SES effects (see Constantinidou et al., 2011). When possible, we used pictures which represented objects (nouns) that are easily recognized and named by young children to ensure that most testees would be very familiar with them. All pictures were static line drawings, meaning they did not depict movement. For tasks that could not involve pictures, we prioritized using numbers instead of letters since numbers are easier to process for those with low or inadequate schooling (Izard et al., 2009; Rasmussen and Bisanz, 2011; Fernández and Abe, 2018). The only exception was the Stroop Victoria (see text footnote 2) task which involves reading words and identifying colors, while all the other stimuli were black, white and gray to allow for testees with dyschromatopsia/color-blindness (up to 8% of males: Chan et al., 2014). To avoid mixing stimuli that would take different naming times in the same task, the required vocal responses to stimuli had similar numbers of syllables (in Portuguese) on the same task (e.g., circle/square, big/small).

Except for the Stroop (inhibition) tasks, which classically present various stimuli on a single page and are not preceded by practice trials, stimuli were presented one at a time to avoid dividing or sharing attention or interference from irrelevant stimuli while viewing each target (see Vidal et al., 2015), which could be differently sensitive to cultural/SES effects. The other tasks were preceded by practice stimuli to ensure testees understood them. However, these were kept to a minimum (enough trials to ensure testees understood each tasks based on pilot studies) because practice may lead people to develop strategies as the task progresses and consequently rely less on executive functions (e.g., Spreen and Strauss, 1998).

In the lower right hand corner of each test page/slide there was a picture showing testees could go on to the next page after each response (self-paced tasks). Testees changed slides themselves by swiping the screen, but clicking a mouse or pressing a spacebar could be alternatives. Testees were asked to complete tasks as quickly as possible while avoiding mistakes. Responses were always vocal so that we could obtain EF measures that would be less contaminated by individual differences on factors such as perceptual/psychomotor speed, coordination, dexterity and laterality, which vary among sexes, change over development and can be affected by SES (e.g., Sullivan et al., 2016), possibly independently from executive functioning.

Throughout the tasks the examiners noted testees’ answers on answers sheets and used a stopwatch to time completion of each block or trial, depending on the task. Slides were numbered to help examiners keep track of responses. The type of answer to each stimulus was suggested in the instructions for each task (e.g., “small”), but similar answers were acceptable if they clearly expressed the same meaning (e.g., “little”). Self-corrected errors were not counted as such because testees’ scores were already penalized by the extra time taken to do so (Lagattuta et al., 2011). Self-corrections also enabled detection of errors that may be of interest when studying EF, such as difficulty choosing correct responses, inhibiting irrelevant information, monitoring performance, correcting and adjusting responses (see Vidal et al., 2015), although this was not analyzed here.

Scores used were RCS, which take speed v. accuracy trade-off into account (see above). Speed of vocal answers and/or motor responses to progress through slides were controlled internally in the inhibition and shifting tasks (absolute cost measures, as per proponents of the model). These tasks include: (1) blocks of trials in which testees use various cognitive abilities but little in the way of specific executive functioning (baseline or control blocks); and (2) one “executive block” which involves the same abilities but has added executive requirement. In these cases, absolute costs were calculated by the subtraction method: performance on the executive blocks minus that in control conditions (control blocks), assuming this “isolates” executive components of the task from other cognitive processes such as perceptual and naming speeds. Controls for speed were not included in the updating task just as they were not in the studies of Miyake, Friedman et al. on adults (e.g., Miyake et al., 2000; Friedman et al., 2008) or studies with younger populations (e.g., St Clair-Thompson and Gathercole, 2006; van der Sluis et al., 2007; Tamnes et al., 2010).

##### Executive tasks

**FIGURE 1:**
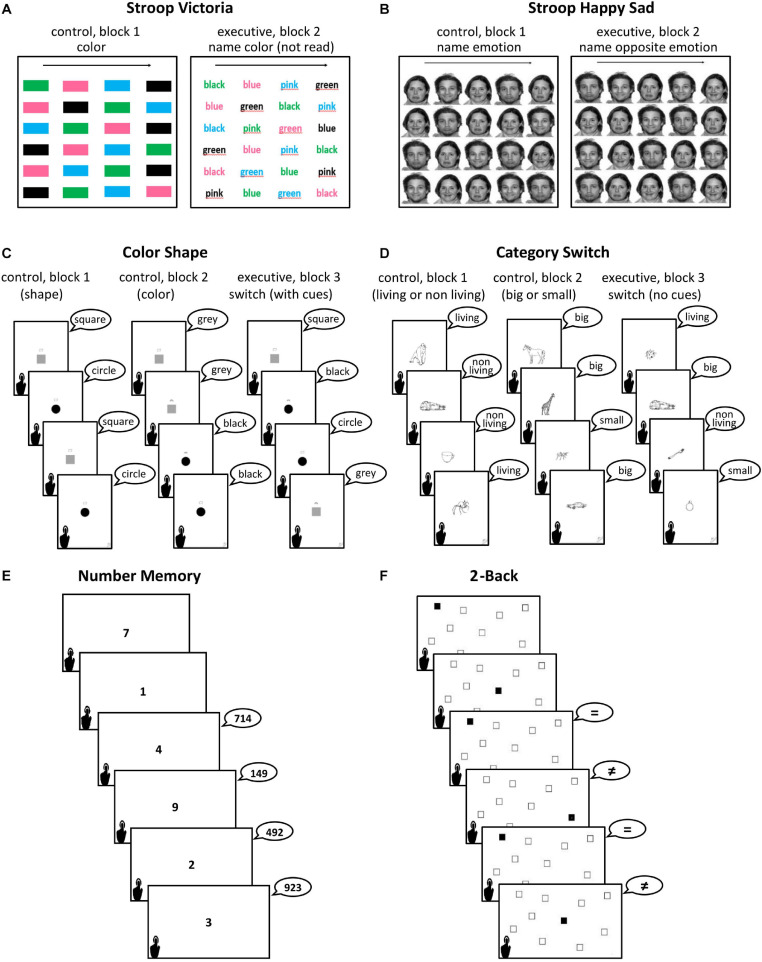
Illustration of the two tasks of each of the three executive domains: inhibition tasks **(A,B)**, shifting tasks **(C,D)** and updating tasks **(E,F)**. All illustrated answers in speech bubbles are correct. See texts and Supplementary Material I for details.

Due to the limited number of words in this Journal, we have briefly described the tasks below. Details of tasks, number of trials and practice trials, stimuli characteristics, and rationale for adaptations may be found in Supplementary Material I. PowerPoint slides showing the tasks themselves are provided to allow other researchers to edit them to fit their local requirements. Details of how to administer and correct tasks, as well as answer sheets for all tasks, are included in Supplementary Material II (in English) and III (in Portuguese). The Executive tasks are illustrated in Figure 1.

###### Inhibition tasks

*Stroop Color-Naming task*: Victoria version (based on Strauss et al., 2006): testees are asked to name (say out loud) the color of 24 stimuli displayed on a single screen per block. In block 1 (baseline, control), the stimuli are color patches. In block 2 (executive block), they are written color names printed in incongruous ink colors (e.g., “green” written in blue ink), which demands inhibiting naming by reading, which leads to faster lexical access than naming the ink color. The main measure of interest was the cost of inhibition (scores on block 2 minus those in block 1). We also provided another block in which stimuli were words that were not colors but were printed in different colors, but we did not use this task here because we followed cost measures used by Miyake et al. (2000).

*Happy Sad Stroop* (adapted from Lagattuta et al., 2011 and Kramer et al., 2015): testees are asked to name the emotions on 20 facial expressions on black and white photographs displayed on the same screen per block. First, they name the expressions they see (happy or sad; block 1, control or baseline). Next (block 2), they must name the opposite emotion to the ones they see (e.g., a happy face must be named as sad, which requires inhibition of automatic emotion naming). Here too the inhibition cost was the main score of interest.

###### Shifting tasks

*Color Shape task* (based on Miyake et al., 2004): consists in classifying pictures (black and gray squares and circles) according to a cue shown just above them. In the first block 20 stimuli are presented under an abstract shape cue figure that testees must classify by shape (circle or square). In the second block, the cue is a rainbow, which indicates that the 20 stimuli are to be classified by color (gray or black). In the third block (executive block), testees answer depending on the cue, which varied from trial to trial, involving switching classifications of 40 stimuli. Blocks 1 and 2 were used as baselines. RCS in block 3 minus the sum of RCS in the prior control blocks indicated shifting costs.

*Category Switch task* (based on Friedman and Miyake, 2004): consists in classifying pictures (black and white line drawings) that represent concrete nouns as entities that may be living or non-living (“dead”) (block 1), big or small compared to a real soccer ball (block 2) and, in the third block (executive block), sequentially switching between the previous classifications, without cues. The number of stimuli and scores were the same as the Color Shape task.

###### Updating tasks

*Number Memory* (adapted from the Letter Memory task; Miyake et al., 2000): the testees are shown single digit numbers on sequential slides. As testees move on from one slide to the next, they are asked to report the last three digits (trios) seen, in the same order as they were shown. As the task progresses, they must continuously update the information held in working memory, discarding the first digit in each trio and adding the new digit that appears next, involving a total 24 updating opportunities. This task does not include a baseline measure and scores are total RCS.

*Spatial 2-Back task* (adapted from Friedman et al., 2008): the testees are shown ten square outlines spread across the screens in fixed locations. On each screen one of these squares is black. As the task progresses, testees must compare the location of the black square they see with the location of the black square two slides back. The required answer is whether the locations match or not (total of 66 updating opportunities, 24 of which required a match response). This task has no control condition and RCS were calculated as per the Number Memory task.

#### Socioeconomic Status

##### Family purchasing power

Determined following guidelines of the Brazilian Market Research Association^4^ (ABEP, 2019; for a version in English^5^). The questionnaire, answered by one of the guardians, attributes points based on the number of items in responders’ homes (i.e., number of cars, motorcycles, bathrooms, refrigerators, freezers, computers, DVDs, washing and drying machines, dishwasher, microwave, full-time housemaid), whether the street where they live is paved, has piped water supply and mean educational attainment of parents/guardians (instead of education of the ‘household’s breadwinner’ proposed in this scale, because many families had difficulty deciding who was their breadwinner). We used the scores obtained from this scale as a continuous variable in the statistical analysis. The ABEP scale was used because other SES measures traditionally used in the international literature involve determining earnings and parental occupation, which is not suitable for Brazil due to its fluctuating economy, rampant unemployment and widespread informal work (see Colom and Flores-Mendoza, 2007).

### Statistical Analysis

Inter-rater reliability was determined by Intraclass Correlation Coefficients (ICC) with 95% confidence intervals with data from 15 participants whose performance was rated by four different examiners. This was done for EF tasks/blocks accuracy and completion time using the SPSS statistical package, version 23 (IBM Corp., 2012) based on a mean-rating (*k* = 4) consistency.

Descriptive statistics were determined for all raw and RCS measures with Statistica software version 13.5 for Windows, also used in the inferential analyses of RCS, which involved univariate General Linear Models (GLM). In these models, performance (RCS per block in each task and RCS inhibition and shifting absolute costs) was the dependent measure, sex was used as a categorical predictor and age (in months) and SES scores were entered as continuous predictors. We also ran a similar model for the updating tasks including another continuous predictor: the average RCS of the control blocks in the inhibition and shifting tasks as a measure of composite speed. We provide *p*-values together with many other estimates from regression models, analyses of variance and effect sizes that are obtained from the GLM: (1) *F*-values and degrees of freedom; (2) coefficient of multiple determination (multiple *R*^2^), or the percent of variance in the dependent variable that is explained by the set of predictors. – We directed more attention to findings with *R*^2^ values of 0.13 to 0.25, considered medium effect sizes, and those above 0.26, regarded as large effect sizes (Ellis, 2010); (3) adjusted *R*^2^, which corrects for number of predictor variables, to allow comparison between models; (4) unstandardized regression coefficients (*B*) for every variable with significant effects to aid in the interpretation of results [there is a one-unit increase in the dependent variable for every increase (positive B) or decrease (negative B) in the coefficient values]; and (5) partial eta squared ($\eta_{\text{p}}^{2}$) for each significant factor: medium effects sizes are usually regarded as between 0.06 and 0.14, while those larger than 0.14 are high effect sizes (Ellis, 2010). Results pertaining to tasks and factors that are not mentioned below did not reach statistical significance. To determine the relation between executive task scores we used Pearson correlations. The level of significance of all these analyses was 5%. We did not adjust for multiple comparison because we explicitly declared *p*-value together with the effect size as recommended.

To test the adequacy of the test battery as a whole, a three-factor confirmatory factor analysis (CFA), using Mplus v. 8.5 (Muthén and Muthén, 2015), was run to try to replicate Miyake et al.’s (2000) model. Following these authors, in the three-factor model we included executive cost measures for the inhibition and shifting tasks but for updating, only total scores (no cost measures were obtained). Unlike Miyake et al. (2000), scores were RCS (not accuracy or reaction times) and latent factors were determined by performance on two instead of three task for each domain, which is acceptable in multifactorial models (Bollen and Davis, 2009). Bayesian inference was used due to the sample size (Lee and Song, 2004; Hoofs et al., 2018; Jacobucci and Grimm, 2018). The fit indices used for evaluating Bayesian CFA were (Hoofs et al., 2018): (1) Bayesian posterior predictive checking using chi-square 95% confidence interval for the difference between the observed and the replicated chi-square values (values that include zero indicate good fit); and (2) posterior predictive *p*-value (PPP: values closer to 0.5 indicate better fit and should not be below 0.05). Convergence criterion was checked via Proportional Scale Reduction (PSR) factor which must be close enough to 1 for each parameter. The priors used for the Bayesian CFA were the *default* implemented in Mplus as specified by Asparouhov and Muthén (2010, p. 58) as follows: factor loading and intercepts normal distribution (0,∞), residual variances inverse-gamma distribution (0,−1), and factor covariances of Inverse-Wishart prior (0,−*p*−1), where *p* is the size of the matrix [in our case, we had Inverse-Wishart prior (0,−4)]. The Monte Carlo simulation was employed next, *post hoc*, to evaluate the robustness of this finding given that the sample size was initially proposed as being equivalent to that in the study on which the model was based (Miyake et al., 2000). Outliers were verified to detect possible measurement errors, but none were excluded in the GLM and CFA.

## Results

Detailed demographics per age are shown in Table 1. Overall, the mean (±SD) age of the sample was 12.1 (±2.0) years. Participants were evenly distributed between sexes. Only two participants (a 10-and a 15-year-old) failed to understand the instructions, which occurred only for the 2-Back task. Performance in these cases was entered in the databank as missing values. Additionally, we had two missing values in the Stroop Happy Sad task, and one in the Number Memory task. No imputation for missing values was used in descriptive analyses, GLM and CFA.

**TABLE 1:** Demographic characteristics of the sample, per age.

**Age (years)**	**Girls (*N* = 80)**	**Boys (*N* = 66)**	**Total (*N* = 146)**	Socioeconomic score* mean (±SD)
9	1	5	6	25.14 (±7.06)
10	10	9	19	21.68 (±6.41)
11	16	9	25	30.04 (±9.55)
12	15	11	26	29.58 (±7.93)
13	11	6	17	29.88 (±10.14)
14	18	13	31	30.33 (±10.82)
15	9	12	21	31.08 (±10.29)

*For illustrative purposes it may be useful to consider the approximate mean monthly family income at the time of the study in US dollars according to the range of points: 45–100 points (about US$ 6,400 or more); 38–44 points (about US$ 2,800); 29–37 points (about US$ 1,400); 23–28 points (US$ 770); 17–22 points (about US$ 440); 0–16 points (about US$ 180) (ABEP, 2019).

Inter-rater reliability metrics for accuracy and speed measures of all blocks/tasks ranged from good to excellent (ICC ≥ 0.90; see Supplementary Material I, Table 1S). Descriptive data for performance on executive tasks are shown in Table 2 in terms of accuracy and completion time for each task to allow comparisons with prior and future studies. Accuracy was very high, as expected for self-paced tasks with adequate instructions and stimuli. Hence, speed differences were the main drivers of effects. Table 2 also presents RCS, a measure that combines accuracy and speed that is efficient in accounting for more variance than either measures of errors and reaction time alone, except when large speed and accuracy effects sizes occur in opposite directions (Vandierendonck, 2018), which was not the case.

**TABLE 2:** Descriptive statistics of raw score and Rate Correct Scores (RCS: accuracy divided by total time in seconds), expressed so that higher scores indicate better performance in all blocks per tasks and absolute executive costs (RCS of executive blocks minus RCS of control blocks) according to each executive domain.

	**Raw scores**		**RCS**						
**Tasks/blocks**	**Speed (s: mean ± SD)**	**Accuracy (no: mean ± SD)**	**Mean (±SD)**	**Confidence interval (**−**95%/+95%)**	**Minimun**	**Maximun**	**Coef. Var**	**Skewness (±SE)**	**Kurtosis (±SE)**
**Inhibition tasks**									
Stroop Victoria – Block 1 (control: name color patches)	17.24 (±4.75)	23.97 (±0.18)	1.48 (±0.36)	1.42/1.54	0.55	2.67	24.51	0.48 (±0.20)	0.72 (±0.40)
Stroop Victoria – Block 2 (executive: name ink of color names)	31.27 (±11.40)	23.36 (±2.10)	0.84 (±0.29)	0.79/0.88	0.03	1.92	34.60	0.53 (±0.20)	1.14 (±0.40)
Stroop Victoria – Inhibition cost (reversed ± sign)	—	—	0.64 (±0.31)	0.69/0.59	−0.04	1.52	47.97	0.32 (±0.20)	0.20 (±0.40)
Stroop Happy Sad – Block 1 (control: name emotion)	16.17 (±4.29)	19.80 (±0.60)	1.30 (±0.31)	1.25/1.35	0.45	2.53	23.89	0.36 (±0.20)	1.42 (±0.40)
Stroop Happy Sad – Block 2 (executive: name opposite emotion)	23.37 (±7.20)	19.30 (±1.32)	0.89 (±0.24)	0.85/0.93	0.34	1.67	26.95	0.22 (±0.20)	0.32 (±0.40)
Stroop Happy Sad – Inhibition cost (reversed ± sign)	—	—	0.41 (±0.24)	0.45/0.37	−0.15	1.14	59.00	0.58 (±0.20)	0.39 (±0.40)
**Shifting tasks**									
Color Shape – Block 1 (control: classify by shape)	21.50 (±5.54)	19.94 (±0.38)	0.98 (±0.23)	0.94/1.02	0.46	1.75	23.28	0.33 (±0.20)	0.41 (±0.40)
Color Shape – Block 2 (control: classify by color)	20.51 (±5.10)	19.94 (±0.26)	1.03 (±0.25)	0.99/1.07	0.50	1.82	24.24	0.41 (±0.20)	−0.06 (±0.40)
Color Shape – Sum control blocks	—	—	1.00 (±0.23)	0.96/1.04	0.53	1.67	22.70	0.33 (±0.20)	−0.02 (±0.40)
Color Shape – Block 3 (executive: switch classification)	78.81 (±18.38)	39.87 (±0.47)	0.53 (±0.12)	0.51/0.55	0.26	0.95	22.22	0.52 (±0.20)	0.57 (±0.40)
Color Shape – Shifting cost (reversed ± sign)	—	—	0.47 (±0.18)	0.50/0.44	−0.07	1.06	38.60	−0.36 (±0.20)	0.59 (±0.40)
Category Switch – Block 1 (control: classify as living/non-living)	26.60 (±9.94)	19.88 (±0.43)	0.82 (±0.23)	0.78/0.86	0.23	1.43	27.86	0.05 (±0.20)	−0.12 (±0.40)
Category Switch – Block 2 (control: classify as big/small)	29.24 (±9.55)	19.52 (±1.00)	0.72 (±0.18)	0.69/0.75	0.21	1.12	25.00	−0.38 (±0.20)	0.03 (±0.40)
Category Switch – Sum control blocks	—	—	0.76 (±0.19)	0.17/0.21	0.23	1.25	24.85	−0.18 (±0.20)	−0.18 (±0.40)
Category Switch – Block 3 (executive: switch classification)	94.30 (±30.16)	35.36 (±6.05)	0.40 (±0.13)	0.38/0.43	0.07	0.83	33.43	0.33 (±0.20)	−0.13 (±0.40)
Category Switch – Shifting cost (reversed ± sign)	—	—	0.35 (±0.16)	0.38/0.33	−0.05	1.03	44.20	−0.52 (±0.20)	2.23 (±0.40)
**Updating tasks**									
Number Memory – (Total score) *	143.33 (±53.00)	21.81 (±3.20)	0.17 (±0.06)	0.16/0.18	0.04	0.31	38.33	0.25 (±0.20)	−0.36 (±0.40)
2-Back – (Total score) *	173.83 (±68.80)	53.45 (±9.77)	0.35 (±0.13)	0.33/0.37	0.08	0.74	40.45	0.69 (±0.20)	0.28 (±0.40)
Composite speed	—	—	1.06 (±0.20)	1.02/1.09	0.55	1.74	19.40	0.21 (±0.20)	0.04 (±0.40)

Stroop Victoria included 24 stimuli per block, Happy Sad Stroop, 20 stimuli; Shifting task control blocks included 20 stimuli, and 40 stimuli in block 3; The Number Memory task included a maximum of 24 updatings, the 2-Back task, 66 updatings; the composite speed was the mean RCS of the control blocks of the inhibition and shifting tasks; absolute costs in the updating tasks were not obtained, following the literature (e.g., Miyake et al., 2000). Kurtosis reported as excess kurtosis; Kolmogorov–Smirnov values were all >0.20 except for Number Memory (= 0.05) and 2-Back (= 0.01). *One outlier removed from the analysis (scores above mean ± 4 SD).

**FIGURE 2:**
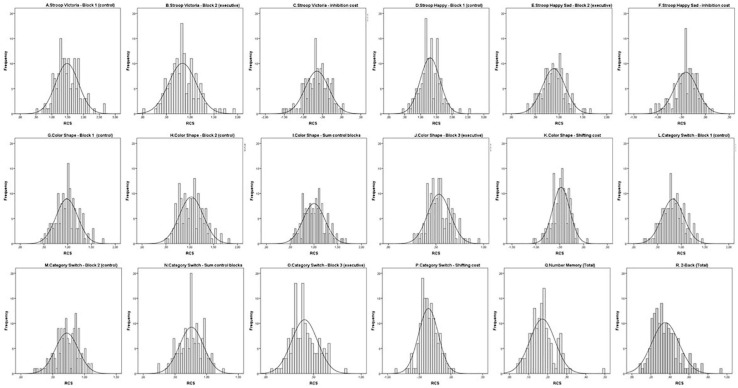
Histograms with the frequency distribution of RCS values in the different blocks of tasks: Inhibition tasks **(A–F)**, shifting tasks **(G–P)** and updating tasks **(Q,R)**. The curve (line) refers to the estimated normal distribution.

**FIGURE 3:**
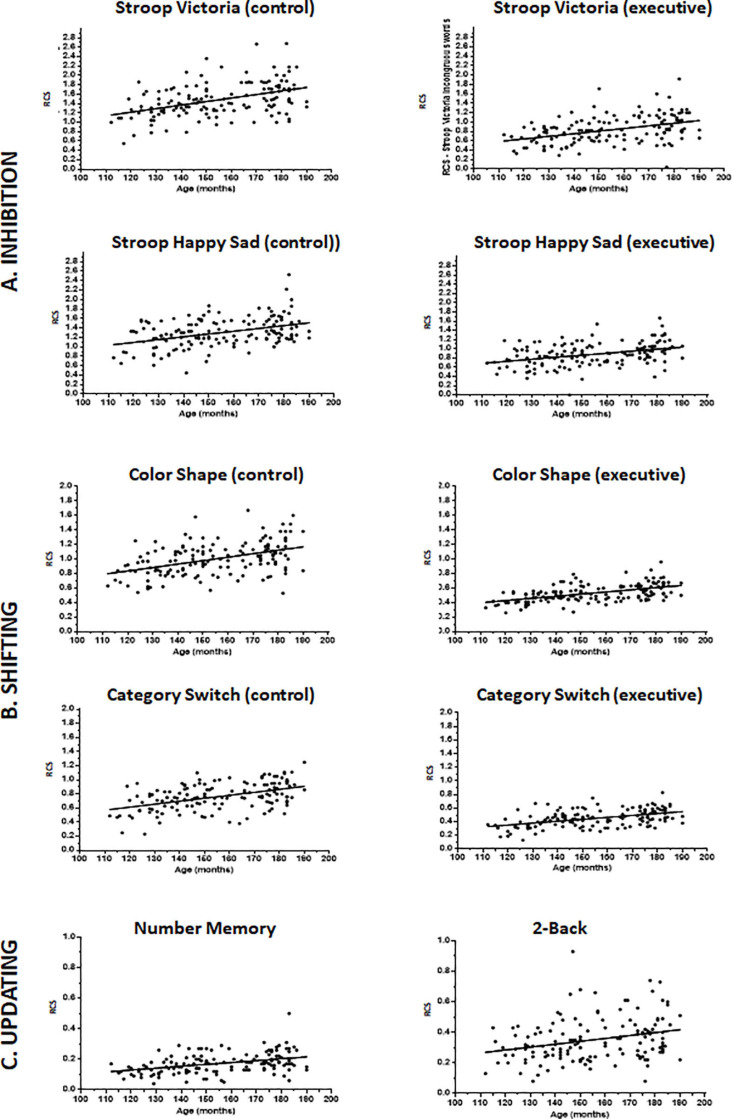
Scatterplot trajectory illustrating Rate Correct Scores (RCS) values per age (months) in the inhibition **(A)**, shifting **(B)**, and updating **(C)** tasks. For inhibition and shifting tasks, scores in the control blocks are on the left, in the executive blocks in the middle and the absolute executive costs are on the far right. Only total scores are represented in the graphs for updating tasks as they do not include control conditions. The regressed line illustrates the association of performance with age, with no correction for socioeconomic status or sex.

We found no evidence of ceiling and floor RCS effects and most distribution metrics (see Table 2 and Figures 2, 3) showed no distortions in data distribution in terms of skewness, kurtosis and normality tests with the exception of 2-Back task [skewness *z*-score (skewness/SE skewness) = 3.50 when cut-offs for normality considering our sample size is 3.29 (see Kim, 2013)] (Table 2 and Figure 2). On the inhibition and shifting tasks the 95% confidence interval values of the RCS of the executive blocks were lower and did not overlap with those of the control blocks (Table 2), showing actual executive costs, that is, greater difficulty in performing the executive blocks than simpler operations such as naming colors and categorizing objects, as expected (see Figure 3). This was confirmed with within-participant repeated measure GLM for each of these tasks with the factor block (two levels: control blocks and executive block). In all cases, RCS in the executive blocks indicated higher difficulty in doing the tasks compared to the control blocks [Stroop Victoria: *F*(1,145) = 634.32; *p* < 0.001; $\eta_{\text{p}}^{2}$ = 0.814; Stroop Happy Sad: *F*(1,143) = 413.698; *p* ≤ 0.001; $\eta_{\text{p}}^{2}$ = 0.743; Color Shape: *F*(1,145) = 980.038; *p* < 0.001; $\eta_{\text{p}}^{2}$ = 0.871; Category Switch: *F*(1,145) = 747.187; *p* < 0.001; $\eta_{\text{p}}^{2}$ = 0.837].

### Effects of Age, Sex and Socioeconomic Status

The joint effects of age, sex and SES explained variance in performance in the GLM of all blocks/tasks with medium to large effect sizes (*R*^2^ from 0.14 to 0.28) with the exception of: (a) the inhibition costs in both the Stroop tasks, which did not reach statistical significance (Table 3); and (b) the small effect sizes in the shifting cost measures (*R*^2^ of 0.04 and 0.06; Table 4) and that of the 2-Back task (*R*^2^ = 0.08; Table 5). In the latter case, adjusting for composite speed (average RCS of the control blocks in the inhibition and shifting task sets) led the model to reach a much higher effect size (*R*^2^ = 0.08 to 0.23), an adjustment that also improved the model of the Number Memory task (*R*^2^ = 0.18 to 0.26).

**TABLE 3:** Results from the General Linear Models for executive **inhibition** tasks/blocks and absolute executive costs, including age, sex and socioeconomic status (SES) effects.

**Tasks/blocks**	**Effects**	***B***	**(df)F**	***p***	**$\eta_{\text{p}}^{2}$**	**Multiple *R*^2^**	**Adjusted *R*^2^**
**Stroop Victoria**	Age (months)	**0.00677**	(1,141) = 25.60	**<0.01**	**0.15**	**0.21**	**0.19**
Block 1 (control: name color patches)	SES (score)	**0.00594**	**(1,141) = 4.20**	**0.04**	**0.03**		
	Sex	−0.01753	(1,141) = 0.41	0.52	<0.01		
Block 2 (executive: name ink of color names)	Age (months)	**0.00529**	**(1,141) = 23.96**	**<0.01**	**0.14**	**0.20**	**0.18**
	SES (score)	0.00405	(1,141) = 2.99	0.08	0.02		
	Sex	−0.02986	(1,141) = 1.84	0.18	<0.01		
Absolute inhibition cost (reversed ± sign)	Age (months)	0.00148	(1,141) = 1.36	0.24	<0.01	0.02	<0.01
	SES (score)	0.00189	(1,141) = 0.47	0.49	<0.01		
	Sex	0.01233	(1,141) = 0.23	0.63	<0.01		
**Stroop Happy Sad**	Age (months)	**0.00526**	**(1,140) = 20.27**	**<0.01**	**0.13**	**0.17**	**0.15**
Block 1 (control: name emotion)	SES (score)	0.00422	(1,140) = 2.74	0.10	0.02		
	Sex	0.02322	(1,140) = 0.94	0.33	<0.01		
Block 2 (executive: name opposite emotion)	Age (months)	**0.00390**	**(1,140) = 16.74**	**<0.01**	**0.11**	**0.16**	**0.15**
	SES (score)	**0.00461**	**(1,140) = 4.92**	**0.03**	**0.03**		
	Sex	0.00067	(1,140) = <0.001	0.97	<0.01		
Absolute inhibition cost (reversed ± sign)	Age (months)	0.00137	(1,140) = 1.68	0.20	<0.01	0.02	<0.01
	SES (score)	−0.00038	(1,140) = 0.03	0.87	<0.01		
	Sex	0.02255	(1,140) = 1.10	0.30	<0.01		

*B, unstandardized regression coefficients; df, degrees of freedom. Bolded values refer to statistical significance.*

**TABLE 4:** Results from the General Linear Models for executive **shifting** tasks/blocks and absolute executive costs, including age, sex and socioeconomic status (SES) effects.

**Tasks/blocks**	**Effects**	***B***	**(df)F**	***p***	**$\eta_{\text{p}}^{2}$**	**Multiple *R*^2^**	**Adjusted *R*^2^**
**Color Shape**	Age (months)	**0.00373**	**(1,141) = 18.21**	**<0.01**	**0.11**	**0.14**	**0.12**
Block 1 (control: classify by shape)	SES (score)	0.00214	(1,141) = 1.28	0.26	<0.01		
	Sex	−0.01224	(1,141) = 0.47	0.49	<0.01		
Block 2 (control: classify by color)	Age (months)	**0.00516**	**(1,141) = 31.51**	**<0.01**	**0.18**	**0.21**	**0.19**
	SES (score)	0.00200	(1,141) = 1.01	0.31	<0.01		
	Sex	−0.00865	(1,141) = 0.21	0.64	<0.01		
Sum of control blocks	Age (months)	**0.00439**	**(1,141) = 27.02**	**<0.01**	**0.16**	**0.19**	**0.17**
	SES (score)	0.00210	(1,141) = 1.32	0.25	<0.01		
	Sex	−0.00993	(1,141) = 0.33	0.56	<0.01		
Block 3 (executive: switch classification)	Age (months)	**0.00279**	**(1,141) = 45.21**	**<0.01**	**0.24**	**0.27**	**0.26**
	SES (score)	0.00105	(1,141) = 1.38	0.24	<0.01		
	Sex	−0.00264	(1,141) = 0.10	0.75	<0.01		
Absolute shifting cost (reversed ± sign)	Age (months)	**0.00159**	**(1,141) = 4.70**	**0.03**	**0.03**	**0.04**	**0.02**
	SES (score)	0.00104	(1,141) = 0.43	0.51	<0.01		
	Sex	−0.00729	(1,141) = 0.23	0.63	<0.01		
**Category Switch**	Age (months)	**0.00373**	**(1,141) = 18.78**	**<0.01**	**0.12**	**0.17**	**0.16**
Block 1 (control: classify as living/non-living)	SES (score)	**0.00381**	**(1,141) = 4.18**	**0.04**	**0.03**		
	Sex	−0.01998	(1,141) = 1.30	0.26	<0.01		
Block 2 (control: classify as big/small)	Age (months)	**0.00382**	**(1,141) = 36.58**	**<0.01**	**0.20**	**0.28**	**0.27**
	SES (score)	0.00246	(1,141) = 3.23	0.07	0.02		
	Sex	−**0.03516**	**(1,141) = 7.45**	**<0.01**	**0.05**		
Sum of control blocks	Age (months)	**0.00384**	**(1,141) = 32.71**	**<0.01**	**0.19**	**0.26**	**0.25**
	SES (score)	**0.00305**	**(1,141) = 4.41**	**0.04**	**0.03**		
	Sex	−**0.02993**	**(1,141) = 4.78**	**0.03**	**0.03**		
Block 3 (executive: switch classification)	Age (months)	**0.00253**	**(1,141) = 26.68**	**<0.01**	**0.16**	**0.24**	**0.23**
	SES (score)	**0.00321**	**(1,141) = 9.11**	**<0.01**	**0.06**		
	Sex	−0.00136	(1,141) = 0.02	0.89	<0.01		
Absolute shifting cost (reversed ± sign)	Age (months)	**0.00130**	**(1,141) = 4.30**	**0.04**	**0.03**	**0.06**	**0.04**
	SES (score)	−0.00015	(1,141) = 0.01	0.90	<0.01		
	Sex	−**0.02856**	**(1,141) = 4.97**	**0.03**	**0.03**		

*B, unstandardized regression coefficients; df, degrees of freedom. Executive costs were reversed signed so that higher scores indicate better performance in all domains. Bolded Values refer to statistical significance.*

**TABLE 5:** Results from the General Linear Models for executive updating tasks/blocks, including age, sex and socioeconomic status (SES) effects.

**Tasks/blocks**	**Effects**	***B***	**(dg)F**	***p***	**$\eta_{\text{p}}^{2}$**	**Multiple *R*^2^**	**Adjusted *R*^2^**
**Number Memory**	Age (months)	**0.00120**	**(1,139) = 22.66**	**<0.01**	**0.14**	**0.18**	**0.16**
Total score*	SES (score)	0.00056	(1,139) = 1.06	0.30	<0.01		
	Sex	−0.00784	(1,139) = 2.33	0.13	0.02		
With control for composite speed	Age (months)	**0.00067**	**(1,137) = 5.93**	**0.02**	**0.04**	**0.26**	**0.24**
	SES (score)	0.00022	(1,137) = 0.18	0.67	<0.01		
	Sex	−0.00609	(1,137) = 1.54	0.22	<0.01		
	Speed	**0.11481**	**(1,137) = 15.88**	**<0.01**	**0.10**		
**2-Back**	Age (months)	**0.00175**	**(1,140) = 9.72**	**<0.01**	**0.06**	**0.08**	**0.06**
Total score*	SES (score)	0.00034	(1,140) = 0.08	0.78	<0.01		
	Sex	−0.01709	(1,140) = 2.21	0.14	<0.01		
With control for composite speed	Age (months)	0.00028	(1,138) = 0.23	0.63	0.04	**0.23**	**0.20**
	SES (score)	−0.00063	(1,138) = 0.30	0.58	<0.01		
	Sex	−0.01125	(1,138) = 1.38	0.24	<0.01		
	Speed	**0.31557**	**(1,138) = 25.08**	**<0.01**	**0.15**		

B, unstandardized regression coefficients; df, degrees of freedom. *Total score refers to RCS without correction for composite speed. The composite speed was the mean RCS of the control blocks of the inhibition and shifting tasks as absolute costs in the updating tasks cannot be obtained due to a lack of control conditions in these tasks, as per the literature. We therefore ran models including this composite as a continuous factor to attempt to correct for perception/psychomotor speed. Bolded Values refer to statistical significance.

Overall, the highest contributing factor in all models was age (except for inhibition costs). Performance significantly improved with age in all individual blocks of all tasks with medium to large effect sizes ($\eta_{\text{p}}^{2}$ for age from 0.06 to 0.28). Differently, age effects on executive costs were much smaller [shifting costs: $\eta_{\text{p}}^{2}$ = 0.03] or were not present at all in the case of inhibition, probably because performance improved with age due to faster responses. The same goes for updating tasks when we controlled for composite speed (Table 5), in which case age effects decreased considerably (Number Memory: $\eta_{\text{p}}^{2}$ = 0.14 to 0.04; 2-Back: $\eta_{\text{p}}^{2}$ = 0.04, reduced to lack of significance).

Only in rare and inconsistent instances (effects found in blocks of one task but not the other one in the same domain) were sex and SES significant predictors of performance. Sex effects favoring girls were inconsistent and of small effect size, having occurred only in the Category Switch task regarding classification by size ($\eta_{\text{p}}^{2}$ = 0.05), and in its shifting cost metric ($\eta_{\text{p}}^{2}$ = 0.03), which also included this same type of classification for half of the stimuli. Similarly, SES effects were small and inconsistent. Lower SES was associated only with worse performance in color patch naming (control block: $\eta_{\text{p}}^{2}$ = 0.03) in the Stroop Victoria task, in the executive block of Stroop Happy Sad ($\eta_{\text{p}}^{2}$ = 0.03), and in the living/non-living classification ($\eta_{\text{p}}^{2}$ = 0.03) and shifting executive block ($\eta_{\text{p}}^{2}$ = 0.06) of the Category Switch task.

### Relations Within Executive Task Scores

Intercorrelations between performance measures (see Table 2S of the Supplementary Material I) were in general higher between performance in blocks of tasks (and cost measures) within the same domains than across tasks and domains. Importantly, performance in all blocks/tasks with executive requirements correlated with each other (*r*s ranging between 0.25 and 0.48), indicating a small-to-moderate degree of overlap across cognitive elements.

### A Three-Factor Model of the Unity and Diversity of Executive Functions

**FIGURE 4:**
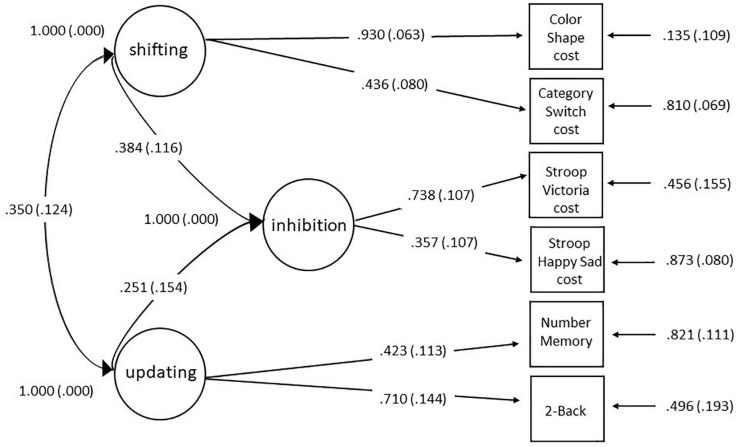
Three correlated factor solution obtain with confirmatory factor analysis of the unity and diversity executive functions model, including factor loadings of each executive measure (represented as squares) on each executive latent variable domain (represented as the ovals). See text for model fits. In the shifting and inhibition measures, scores were reversed ± signed so that higher scores always indicate better performance.

The three-correlated factor solution of the confirmatory factor analyses (Figure 4) fit the data well: 95% CI chi-square = −7.841 to 35.613. Concerning PSR, Table 3S of the Supplementary Material I shows that only 8500 iterations for convergence of the model below 1.1 were necessary. Indeed, the parameter values did not change across the running of the iteration and the PSR still remains close to 1 (see Asparouhov and Muthén, 2010). PPP = 0.140 was acceptable. The model with its factor loadings, error and correlations among latent variables is shown in Figure 4. The reliabilities for each task were: Category Switch cost = 0.444 (95% Credibility Interval [CrI] = 0.267 to 0.627), Color Shape cost = 0.856 (95% CrI = 0.562 to 0.995), Number Memory = 0.367 (95% CrI = 0.100 to 0.632), 2-Back = 0.689 (95% CrI = 0.297 to 0.990), Stroop Victoria cost = 0.408 (95% CrI = 0.113 to 0.919), and Happy Sad Stroop cost = 0.271 (95% CrI = −0.007 to 0.601).

Results of the Monte Carlo simulation can be found in Table 4S of the Supplementary Material I and showed the sample size to be robust enough in different aspects following three criteria proposed by Muthén and Muthén (2002): (1) bias of the parameters and their standard error; (2) the proportion of replications for which the 95% confidence interval contains the true population parameter value; and (3) power, or the percentage significance coefficient. However, it should be borne in mind that the inhibition latent factor was underpowered in respect to the power criteria due to the low factor loadings of the inhibition cost scores on their latent factor.

## Discussion

This study aimed to determine the adequacy of the FREE battery to assess the unity and diversity model of executive functions (Miyake et al., 2000) in a young sample with variable SES from a developing country. Inter-rater reliability was good to excellent (Koo and Li, 2016) and, overall, we found that performance on the tasks assessed with RCS, which combines speed and accuracy (Vandierendonck, 2018): (1) was generally distributed symmetrically, not heavily or lightly tailed relative to a normal distribution and did not indicate having reached ceiling and floor effects, making them adequate for most types of statistical analyses; (2) was sensitive to improvement with age, except in measures of inhibition costs, reflecting the expected developmental trajectory of executive functions; and (3) displayed no consistent evidence of affect of sex, as found in most studies on executive functions, nor of SES, indicating that it may be appropriate for use in samples with varying socioeconomic inequalities. Most importantly, the unity and diversity structure of the model found in WEIRD adults (Miyake et al., 2000) was replicated, although we used a younger, culturally and socioeconomically diverse population compared to the one of the original study. Each of these points will be detailed below.

Firstly, the selection of stimuli, type of presentation, response mode and performance metric seem to have been adequate as data distribution was largely normal (see Kim, 2013) using tasks with the following characteristics: (1) presenting easily recognizable stimuli (Izard et al., 2009; Rasmussen and Bisanz, 2011; Fernández and Abe, 2018); (2) using self-paced (Schweizer et al., 2019) tasks; (3) requiring vocal responses (Wang and Proctor, 1996); (4) allowing for self-corrections (Vidal et al., 2015); and (5) use of scores in the form of RCS (Vandierendonck, 2017). Data distribution such as this is important because the use of the most powerful inferential statistical procedures are often affected by the presence of positively or negatively skewed, flattened, or steep distribution (see Cramer and Howitt, 2005). There was also no indications of ceiling and floor effects that could have distorted statistical analyses (see Cramer and Howitt, 2005).

For the inhibition and shifting tasks, performance was worse in the executive blocks than in their respective control blocks, indicating clear absolute executive costs, which are rarely explicitly shown in most publications. Unlike our study, in which RCS scores were used, prior investigations either report accuracy/error or speed costs without a specific explanations for choice of metric, even though they seldom correlate, leading to statistical distortions that can hinder the comparability of results from different studies (Tamnes et al., 2010; Hedge et al., 2018).

Except for inhibition tasks, performance in all individual blocks of all the other tasks in our test battery improved with age with medium and large effect sizes, as expected due to improvement in executive functioning that has been reported for adolescents (Huizinga et al., 2006; Tamnes et al., 2010; Wu et al., 2011; Lee et al., 2013; Xu et al., 2013). The same was observed more specifically for executive components assessed with the shifting cost measures (small effect size), corroborating prior studies (Huizinga et al., 2006; Wu et al., 2011; Lee et al., 2013; Xu et al., 2013). Tamnes et al. (2010), however, showed no improvement in two shifting tasks between the ages 8 to 19 years, having attributed this to the low reliability of their measurements, which in their case only involved reaction times. It is possible that the use of RCS in our study favored the appearance of this effect, especially because the effect sizes were very small, which makes detection difficult.

The lack of improvement with age in prepotent inhibition costs in both Stroop tasks was not unexpected. This ability matures rapidly in preschool years (up to about age 10 years; see Wu et al., 2011), becomes stable for some years (Best and Miller, 2010; very similar between aged 10 and 15: Lee et al., 2013; between 7 and 12 for Xu et al., 2013) and then improves again some years later (Xu et al., 2013; Theodoraki et al., 2019) or after the maximum age of our sample (15 years: Huizinga et al., 2006; Tamnes et al., 2010; Poon, 2018). Differently, others have shown that inhibition is relatively stable from age 7 until early adulthood (see Carriedo et al., 2016). This contrast between effects of age across studies may indicate that the tasks used in different publications tap different types of inhibition (Friedman and Miyake, 2004), such as resistance to proactive interference (not assessed here), which seems to steadily increase from childhood to late adolescence or early adulthood (see Comalli et al., 1962; Carriedo et al., 2016). We did not assess this latter type of executive function as it is not included in Miyake et al.’s (2000) model, so we cannot confirm this hypothesis.

The improvement in performance with age in the updating tests (not originally corrected for composite speed) was also verified by Huizinga et al. (2006), Schleepen and Jonkman (2009), Tamnes et al. (2010), Lee et al. (2013), and Xu et al. (2013). The updating tasks used here and in the latter studies, however, did not have a control block, following studies of the unity and diversity of executive functions (e.g., Miyake et al., 2000; Friedman et al., 2008). Studies in this field do not address the reason for this lack of control, possibly because in these publications stimuli are presented for specific time intervals, unlike in our self-paced task. Fixing time limits to respond is not advisable when using children and adolescents of different ages because perceptual and psychomotor speed changes rapidly with age (Kail, 1991; Fry and Hale, 2000; Rigoli et al., 2012; Ludyga et al., 2019). Hence older individuals have an advantage that might not be executive in nature. Furthermore, it has been shown that when time of exposure is limited, testees miss responses, leading to different sets of results among participants, which can distort statistical findings (Schweizer et al., 2019). Adding a speed control task to updating measures, such as a zero-Back condition, is not straightforward. Some studies have shown that the developmental trajectory for zero-Back and 1-Back tasks are not the same as for 2- and 3-Back conditions (e.g., Schleepen and Jonkman, 2009) in terms of false alarms, reaction time and percentage of hits, so using the subtraction method with this type of control condition is not adequate. We tried to overcome this by controlling performance in the updating tasks with a composite measure of speed (mean RCS in the control blocks of the inhibition and shifting tasks, in which there was no specific executive requirement). This led to a decrease (Number Memory task) or disappearance (2-Back task) of effects of age on updating. Admittedly, RCS of the 2-Back task were slightly skewed beyond ideal metrics of normality, but this seems unlikely to be the only explanation for lack of age effects since results were similar to those of the other updating measure (Number Memory task). The contrast of (1) the medium to large age effects in all blocks of all tasks on the one hand and (2) the small or null age effect in inhibition and shifting costs and updating (corrected for composite speed) on the other, reinforces that a great portion of improvement in executive functioning in early adolescence can be explain by general improvement in factors such as naming, psychomotor and processing speed (e.g., see Fry and Hale, 2000; Sheppard and Vernon, 2008; Coyle et al., 2011; McAuley and White, 2011; Rose et al., 2011; see also Lee et al., 2013; Sullivan et al., 2016). This age effect may mirror improvement in intelligence, which is related to EF (see Friedman and Miyake, 2017), since it is positively associated with speed, as discussed by many authors (Sheppard and Vernon, 2008; Duan et al., 2010; Coyle et al., 2011; Rose et al., 2011; Lee et al., 2013).

Consistent evidence of sex effects were not found here, corroborating most studies (e.g., Huizinga et al., 2006; Tamnes et al., 2010; Wu et al., 2011; Xu et al., 2013; Grissom and Reyes, 2019). We only observed a female advantage (small effect size considering partial eta squared) in classifying stimuli by size in the Category Switch task which may relate to the female advantage in mental imagery (White et al., 1977). This must be recruited to compare the real size of objects/entities with a real soccer ball. Nonetheless, our main measure of interest in this task was the shifting cost, which showed lack of evidence for sex effects.

Accordingly, SES effects appeared only in some individual blocks, reached small effect sizes and were inconsistent, suggesting that our test battery was adequately adapted to minimize these effects. This occurred despite SES being known to be associated with negative impacts in brain development (Brody et al., 2017; Foulkes and Blakemore, 2018) and executive functioning (Haft and Hoeft, 2017; Zhang et al., 2019). It is unlikely that this could have resulted from low sensitivity of the SES score used here (ABEP) to cognitive abilities, because it has been found to be positively related to cognitive measures, including executive functions at various ages (e.g., Moraes et al., 2010; Piccolo et al., 2016), attesting its adequacy as a general measure of SES. Hence, it may be that part of the SES effects shown in the literature might stem from the use of tests that are not developed to be used in low SES individuals, unlike the present ones. Stated differently, tasks devised for WEIRD populations, when used in less privileged people, may lead to SES effects that are at least partly due to task requirements that are not executive in nature, but dependent on other cognitive abilities. Indeed, we found that lower SES individuals had more difficulty in classifying pictures as living/non-living entities in the Category Switch task, but not by size, colors or shapes, which were the other classifications in the shifting tasks. Non-living things are more difficult to name because they have more representations in the real world and lower proportion of interrelated properties than living entities, irrespective of concept familiarity, word frequency or visual complexity (Laws and Neve, 1999). This has been found to be harder to do by low SES individuals, possibly because of non-executive difficulties in access to semantic attributes (Barea and Mansur, 2007). We were unaware of this when we adapted this task. Future studies should take this into account and find alternative classification categories that are easier for low SES individuals (consider using animal vs. non-animal). It should also be considered that using various tasks with inadequate stimuli in low SES samples may inflate the chance that SES effects are observed (see Lawson et al., 2018).

Higher SES participants were also faster at naming incongruent emotions in the executive block of the Happy Sad Stroop, which is based on the fact that affective information in facial expressions is perceived involuntarily and may constrict the focus of attention (Balconi and Lucchiari, 2005), so naming the “opposite” emotion taxes executive inhibition. Childhood poverty has been found to be associated with altered brain activation to facial expressions in adulthood (Javanbakht et al., 2015) and difficulties in tasks that involve social cognition (see Foulkes and Blakemore, 2018). This indicated that it is possible that factors associated with low SES may make it harder to label some emotions. Lack of SES cost effects on this measure, however, does not support this hypothesis, suggesting that these SES effects may have been due to other unknown effects or reflected random differences. It should be mentioned that the Happy Sad Stroop may not be a good measure of inhibition for testees with social cognition difficulties such as autism spectrum disorders. Nonetheless, it can be used as an alternative to the Stroop Victoria, which has limited utility for people with no or low reading skills and those with dyschromatopsia (color blindness). SES affects on naming color patches in the Stroop Victoria (Block 1) could indicate lower automatized naming, but was more likely a spurious effect because no evidence of SES effects were found for naming shapes and sizes, or black/gray colors in the Color Shape task.

Importantly, we found lack of evidence that SES impacts any measure of inhibition and shifting costs or executive updating, which were the key measures of interest that our test battery was designed to assess, even though people with low access to cognitive stimulation, or low quality education, are particularly susceptible to executive impairment for a variety of reasons (Hackman and Farah, 2009; Hackman et al., 2015; Diamond, 2016). This was true even for the Stroop Victoria task, which only measures inhibition if testees have automatized reading to some degree, which is usually better in those with higher SES (Evans et al., 2010; Thomson, 2018). Notwithstanding, this task should obviously not be used for illiterate individuals and was shown to form a weaker latent factor with the other inhibition task in the CFA. It was included (see text footnote 2) in this test battery because we were unable to find another measure that was as sensitive to inhibition of prepotent responses while meeting our criteria (e.g., no copyright, application in blocks, affordable equipment and software). Overall, the use of tests that were developed to minimize the impact of SES showed promise in making scientific findings on executive functioning more representative of humankind (Rad et al., 2018; Fernández and Abe, 2018), whereas tasks devised in WEIRD countries are representative of a small and non-prototypical population (Henrich et al., 2010; Rad et al., 2018).

Finally, like many others that used Miyake et al.’s (2000) model as a basis, we were able to show EF unity and diversity in a three correlated factor model solution with appropriate psychometric properties, despite having used a young population from a developing nation including very low SES individuals. Similar findings to ours regarding latent factors for early adolescents were reported by Duan et al. (2010) in 11- to 12-year olds, Xu et al. (2013) with 7- to 15-year olds (although the best fits were found in the models of 13- to 15-year olds), Wu et al. (2011) for ages of 7 to 14 years, but with only one measure of updating, and Lee et al. (2013) in their older adolescents. It is noteworthy that only the latter studies used tasks that reflect the separable domains proposed by Miyake et al. (2000). Other investigations at this phase of life showed different factors structures (e.g., unitary model: Xu et al., 2013; two-factor models: St Clair-Thompson and Gathercole, 2006; van der Sluis et al., 2007; four factors: Hartung et al., 2020; see Karr et al., 2018 for a review). This could have been due to additions of other executive domains (e.g., Hartung et al., 2020), misunderstandings about domain descriptions (see Morra et al., 2018), such as: using maintenance of information in working memory (working memory capacity) as a proxy for updating (e.g., Lehto et al., 2003; Agostino et al., 2010; McAuley and White, 2011; Rose et al., 2011; Arán-Filippetti, 2013; Theodoraki et al., 2019), and set shifting or cognitive flexibility in place of shifting (e.g., Lehto et al., 2003; Latzman and Markon, 2010; Shing et al., 2010; Arán-Filippetti, 2013; Poon, 2018; Li et al., 2019; Theodoraki et al., 2019).

Regarding the Monte Carlo simulation used to assess the adequacy of the sample size for the CFA, we found that, in general, the number of tested individuals was acceptable following three criteria proposed by Muthén and Muthén (2002). The exception, regarding high deviation for one of these criteria, occurred for the inhibition factor, which seemed to be underpowered. This does not invalidate our CFA model for two main reasons. First, not all of the three criteria used here must be met for all parameters; instead, the extent of the deviation from the ideal metrics must be considered in general terms to describe sample size adequacy for a model of interest (Muthén and Muthén, 2002). Secondly, the fact that inhibition cost scores formed a weaker latent factor is not surprising as prior work has found that inhibition of prepotent responses matures after the maximum age limit of our sample, as mentioned above (see Huizinga et al., 2006; Tamnes et al., 2010; Xu et al., 2013; Poon, 2018; Theodoraki et al., 2019). This must be confirmed in the future in samples of adolescents and adults, which may enable other psychometric properties of the FREE test battery to be determined. Other factor structures were not explored because our aim was to verify if the test battery allowed the latent factors to be distinguishable in adolescents based on Miyake et al.’s (2000) model. We did not intend to propose other model configurations, nor determine the best factor structure for our sample as neither alternative would speak to the adequacy of the test battery itself, which was our intent in this study.

In sum, the test battery proposed here met all adequacy requirements as a potential tool for assessing the unity and diversity of EF in diverse populations, as long as the instructions and stimuli are adapted following our suggestions (e.g., use of instructions, words, pictures and numbers familiar to the population under investigation). The distribution of scores showed that our tests were not too difficult or easy and that our choice of metric (RCS) was adequate. Content-related evidence of validity (Sherman et al., 2011) was assured by selecting tests based on a theoretical model supported by literature reviews (e.g., Friedman and Miyake, 2017; Karr et al., 2018), and by defining the criteria for selecting tasks and stimuli, based on theory. Our findings of the expected demographic (no consistent sex effect) and developmental trajectories indicated criterion-related validity. Importantly, SES effects, when present, were small and inconsistent, indicating that the stimuli were easily processed by testees. Although we tested the tasks only in early adolescents, we believe that the FREE test battery may also be used for other ages (from age 9 years, the earlier age tested here) because: (a) the tasks, numbers of trial, etc., were selected from studies that assessed adults; and (b) EF diversity is observed as of adolescence. This must be explored in coming studies.

Regarding limitations, although our sample size matched that of the study that proposed the model we sought to replicate (Miyake et al., 2000), it could have been larger, included more age ranges, and more diverse populations in terms of cultures, etc. This would have enabled us to determine invariance (metric, scalar and residual invariance testing by age, sex and SES: see Putnik and Bornstein, 2016). Our primary objective, however, was to point out the characteristics of executive tasks that can influence performance in diverse populations, show that it is possible to design open access tests that can distinguish inhibition, shifting and updating at the latent factor level and that can be adapted and administered to both non-WEIRD and WEIRD populations. Another limitation is that the results of the GLM were not corrected for multiple testing, which can reduce false positives but at an expense of increasing false negatives. We did not do so because: (1) a method to do so in this particular type of study has not been established or consistently used in the literature; and (2) determining False Discovery Rates *a priori* would have been subjective seeing that very few published works have tested performance on the domains of executive functioning of interest in the same age range from non-WEIRD samples (Nyongesa et al., 2019). The latter reason also made it unreasonable to use approaches such as equivalence tests, in which the null hypothesis is defined as an effect that is large enough to be considered “interesting” based on results of prior studies. Here we were dealing with a theory that poses that each executive domain measures a different yet correlated ability. Our approach to confirm this was to look at the data in three different ways. Firstly, we used results of the GLM to look for consistent patterns of effects between the two measures of each domain. We then contrasted this pattern with the expected results in the literature and found that the findings corroborated prior studies. The suitability of the tasks selected as representative of each domain was confirmed by the CFA, as both tasks of each domain formed a latent factor, evidencing their shared variance and that the three latent factors ware also interrelated (convergence validity), indicating the diversity and unity of executive functions, respectively. Concurrent validity in comparison to other EF measures should be tested from this time. In this respect, we underline that all tasks used here were selected because they present prior evidence of being represented of the executive domains proposed by Miyake et al. (2000), so there is no reason to suppose that they should not index each of the EF facets. Differently, future studies should determine discriminatory validity by comparing performance in the proposed tasks with scores on tasks that tap different cognitive domains, which we did not evaluate.

The FREE test battery proposed here is a prototype and must be improved upon in future studies and assessed in samples of different ages, backgrounds, SES and cultures, which may use the tasks we proposed as long as: (1) participants can read and are familiar with numbers; and (2) instructions and stimuli are adapted to be easily understood and recognized locally by the great majority of the population of interest, especially those who had less access to adequate formal education to reduce any possible performance disadvantage compared to better schooled individuals. Additionally, the tasks can be modified and alternative scoring metrics other than RCS may be used (according to data distribution and preference of researchers). We also hope that new tasks per domain can be proposed so that the CFAs can include more than two tasks per domain. This is ideal, although our model with two observed measures per domain is identifiable because it is multifactorial (see Marsh et al., 1998; Bollen and Davis, 2009). This is especially true considering that the tasks we succeeded in adapting were quite similar within domains and because we failed to find two inhibition measures that did not rely on reading, an ability that is highly affected by SES and that formed a weaker latent factor. Details on tasks and task administration and correction are provided in Supplementary Material I (detailed task description, results of inter-rater reliability and details of the CFA), II (manual in English) and III (manual in Portuguese) and the tasks themselves may be downloaded and modified from https://osf.io/2bx8n/?view_only=c42ee8e677e94f85a618bb2640c12b5c (Zanini et al., 2020a). Following open science principles, the FREE tasks may be used with inexpensive equipment and are open access, thus facilitating replication. We emphasize, however, that these tasks were designed for research purposes rather than diagnosing neuropsychological disorders, for which other factors must be taken into account (see Brickman et al., 2006; Manly, 2008; Olson and Jacobson, 2015; Howieson, 2019).

## Data Availability Statement

The datasets presented in this study can be found at https://osf.io/2bx8n/?view_only=c42ee8e677e94f85a618bb2640c12b5c (Zanini et al., 2020a).

## Ethics Statement

The studies involving human participants were reviewed and approved by Comitê de Ética em Pesquisa - Universidade Federal de São Paulo (UNIFESP). Written informed consent to participate in this study was provided by the participants’ legal guardian/next of kin.

## Author Contributions

SP, GZ, MM, and HC-M designed and planned the study. GZ, SP, MM, and HC-M collected, analyzed, and interpreted the data. AN and AF critically reviewed the final manuscript. All authors approved the final version to be published. SP contributed to the public responsibility for the content of the article. All authors contributed to the article and approved the submitted version.

## Conflict of Interest

The authors declare that the research was conducted in the absence of any commercial or financial relationships that could be construed as a potential conflict of interest.

## Footnotes

1 Subsequent to the proposal of the three-correlated factor model, another configuration was shown to have a better explanatory power [called “nested factor multivariate executive function” by Friedman et al., 2008, “bifactor parametrization” by Friedman and Miyake, 2017 or “nested factor” (Karr et al., 2018)]. This alternate model includes (see Friedman and Miyake, 2017): (1) a general factor that predicts performance on all of this model’s variables (unity), which was considered by the authors as isomorphic (*sic*) with inhibition; and (2) shifting and updating orthogonal latent factors (diversity), which capture the remaining covariance between the performance in these domain-specific tasks when common EF variance is controlled for. Here, we have focused on the original three-correlated factor solution because a recent study (Eid et al., 2017) showed that models such as the latter (named symmetrical bifactor models) do not include covariance between the specific factors other than the general factor (in this case, shifting and updating), generating psychometric anomalies that may alter model interpretability. Therefore, because this model is not clearly specified in psychometric terms it must be tested with this added covariance to be confirmed as a good solution.

2 Inhibition tasks in studies of EF unity and diversity involved registering response times to key presses for each stimulus, unlike the present proposal. We piloted many inhibition measures that were monochromatic, did not involve words and could be carried out by measuring time taken to complete blocks with many stimuli each, including various versions of Animal Stroop tasks, Numeric Stroop tasks, Fruit Stroop, Day and Night Stroop, and others. None of these showed inhibition costs. Difficulty in finding adequate inhibition tasks that fulfilled our task criteria led us to include: (1) the Happy Sad Stroop task, which presented clear executive costs in pilot studies and in the literature (Lagattuta et al., 2011; Kramer et al., 2015) but was not used in prior studies that assessed the EF unity and diversity model; and (2) the Stroop Victoria task since it shows clear executive costs, despite its use of words and colors which we had aimed to avoid.

3 The tasks were devised to allow instructions and stimuli to be printed on sheets of paper or cards, but the effectiveness of this mode of presentation was not tested here.

4 http://www.abep.org.br

5 http://www.abep.org/Servicos/Download.aspx?id=11

## Supplementary Material

These supplementary files are also includedin the Open Scinece Framework at https://osf.io/2bx8n/?view_only=c42ee8e677e94f85a618bb2640c12b5c (Zanini et al., 2020a).
