# Supplementary material for: An Adaptable, Open-Access Test Battery to Study the Fractionation of Executive-Functions in Diverse Populations
Source: Front Psychol. 2021 Mar 30;12:627219. doi: 10.3389/fpsyg.2021.627219 (PMC8042159; doi:10.3389/fpsyg.2021.627219)
Supplement: Supplementary file 1 [file Data_Sheet_1.pdf]

Supplementary Material I

Find below a detailed description of tasks in the Free Research Executive Evaluation (FREE) test battery. The tasks themselves, in pdf and pptx versions are also available. Details on how to administer, take down and correct answers, as well as the answer sheets can be found in the administration and correction manual (in English: supplementary II; in Portuguese: supplementary III). The rationale behind the test battery is described in the main document "An adaptable, open-access test battery to study the fractionation of executive-functions in diverse populations".

1. Detailed task description of the tasks

Inhibition tasks

Stroop Color-Naming task, Victoria version [based on Strauss et al., 2006), adapted and modified from the adaptation for use in Brazil by Duncan (2006)]: This task measures executive inhibition of automatic responses (Miyake et al., 2000; Cothran et al., 2008; Diamond, 2013). The Victoria version, which is shorter than the original Stroop Color-Naming task, was selected because it shows less learning effects during the task (Spreen and Strauss, 1998). The task consists of three blocks but we only report data on blocks 1 (control block) and 3 (executive block) as in most works that studies the fractionation of executive functions (e.g. Miyake et al., 2000). Block 1 (color patches) includes 24 colored rectangles, measuring one centimeter in height and three centimeters in width each, distributed in six lines with four rectangles in each row on a single portrait-oriented page/slide. Each color is presented six times each, ordered pseudo randomly (www.randomizer.org/: avoiding more the two consecutive presentations of the same stimulus). The space between lines is of one cm and the space between the stimuli in each line is two cm. There is a top and bottom margin of 6.4 cm. The colors of the stimuli are pink, green, blue and black [instead of brown present in Duncan's (2006) version because this word in longer to pronounce in Portuguese than the other colors, unlike black]. Here, the following colors in hue, saturation and luminosity (HSL) values from PowerPoint were used: pink (hue: 241; saturation: 255; luminosity: 179), green (hue: 104; saturation: 255; luminosity: 88), blue (hue: 139; saturation: 255; luminosity: 120) and black (HSL color in PowerPoint: hue: 170; saturation: 0; luminosity: 0). These colors appear only once in each row. Testees are asked to name the colors patches from left to right, as fast as possible. Above the first row of stimuli there is a horizontal arrow pointing right to remind testees to name the colors in this order. In block 2, stimuli are positioned on similar screens but consist of common words that are not color names (in Portuguese "casa, hoje, tudo, nunca", which mean "house, today, everything, never") printed in lower case [as in the versions of Spreen and Strauss (1998), but unlike Duncan's (2006) capital letter presentation] Calibri font size 32 in the same colors as those in block 1. Testees are again asked to name the colors, and not to read the words (we do not describe data for this block here). In block 3 (incongruent; the executive block) testees must do the same, but the stimuli are color names "pink, green, blue and black" (in Portuguese: "rosa, verde, azul, preto") distributed on the page and printed in the same font as in block 2, but in incongruent colors (e.g., the word "pink" printed in "blue" ink).

Testees are asked to follow instructions, give their responses aloud (vocal response) and do the task as fast as possible avoiding mistakes. The examiner writes down the answers on answers sheets and records the time testees take to complete each block.

The scores were RCS of each block (correct responses in each one divided by the time in seconds taken to complete each block) and RCS inhibition cost: the RCS of block 3 minus the RCS of block 1.

Happy Sad Stroop [adapted from Lagattuta et al. (2011) and Kramer et al. (2015)]: This task measures executive inhibition of automatic responses. The target stimuli were obtained from the NIMSTIM facial expression database (Tottenham et al., 2009) and were chosen based on a pilot study with 78 young adults, who were asked to name the emotions expressed on each face (data not shown). We selected the images of actors (one male and one female) whose sad and happy faced were rated as such by over 90% of the volunteers, as in the work of Lagattuta et al. (2011), because it must be ensured that target emotions are not ambiguous. The chosen photographs were from models 7 (female 07F_HA_C, 07F_SA_C, 36M) and 34 (male 34M _HA_C and 34M_SA_C). The task includes three blocks, but only scores of blocks 2 and 3 will be reported. Block 1 was a "warm-up" block (not included in the original test nor assessed here) with black and white emoji measuring 3.5 cm in diameter with sad and happy expressions (10 each), arranged on screen in a 5 x 4 matrix totaling 20 stimuli. Above the stimuli there as a horizontal arrow pointed right to remind participants to name the emotions from left to right. In pilot studies we found that this block ensured saying the emotions was sufficiently practiced before the first target block was presented. Block 2 and 3 involve black and white photographs (4 x 3.67 cm) distributed on screen as in block 1, including the horizontal arrow. These blocks included the images of happy and sad faces of both actors, presented five times each, ordered pseudo randomly (www.randomizer.org/: avoiding more the two consecutive presentations of the same stimulus). In blocks 1 and 2 (control blocks), testees are asked to name the expressions on the stimuli, from left to right. In block 3 (incongruent or executive block), testees are asked to do the opposite: say "sad" for happy faces and "happy" for sad faces.

Testees are asked to follow instructions, give their responses aloud (vocal response) and do the task as fast as possible avoiding mistakes. The examiner writes down the answers on answers sheets and records the time testees take to complete each block of each task.

The scores were RCS of block 2 and 3 (correct responses in each one divided by the time in seconds taken to complete each block). RSC inhibition cost was the RCS of block 3 minus the RCS of block 2.

Shifting tasks

Color Shape task [based on Miyake et al. (2004) and Friedman et al. (2008)]: This task assesses executive shifting and was altered from the original version [in which the stimuli consisted of green or red colored rectangle centered onscreen within which there was either a circle or triangle (target shapes)]. In the original versions, just above the target stimuli there appeared either the letter C or S indicating what the participant should answer: C referred to classifying the "color" of the rectangle and S (cues) to classifying the "shape" included in the rectangle. During the adaptation of the task for the present purposes, the following adaptations were made: the external rectangle was not used as a stimulus to prevent testees from confusing its shape with that of the target stimuli themselves. Therefore, only the shapes that were inserted inside the rectangle were kept. We used circles and squares (instead of triangles), because the word triangle is longer to pronounce in Portuguese and because some children had difficulty naming it in pilot studies. The colors used in the original work, green and red, were also replaced for black and gray to avoid having to exclude testees with any difficulty in color discrimination. The letters C and S used as clues in the original work were replaced

with symbols to avoid possible difficulties in low or badly schooled individuals, especially as both these letters can have similar sounds when placed at the beginning of words in Portuguese. Thus the cue letter "C" used as a cue for the "Color" responses was replaced with a monochromatic rainbow symbol, and the letter "S", used as a cue for the "Shape" responses, was replaced with an abstract, non-namable black and white shape.

The task was built on PowerPoint slides in portrait orientation. Target stimuli are squares 3 cm in height and width and circles 3 cm in diameter presented individually, either in black (HSL color in Power Point: hue: 170; saturation: 0; luminosity: 0) or gray (HSL color in Power Point: hue: 170; saturation: 0; luminosity: 166), centered on the screen so that there is around 10 cm from the bottom and top, and 8 cm from the sides. One centimeter above each stimulus there are cues (rainbow or abstract shape) approximately 1.0 cm in height and 1.5 cm in width.

The task consists of three blocks (the first two are control blocks; the last, the executive block), all preceded by 4 practice trials using black and gray circles and squares. In block 1, participants are asked to name the shapes they see, all of which have the cue (abstract shape) above the stimuli. In block 2 they must name the color of the shapes, all of which have a rainbow shaped cue above them. In block 3 (shifting block) testees must classifying the stimuli (shape or color) according to the clue placed above the stimuli, which varies from slide to slide. Blocks 1 and 2 contained 20 target stimuli each, while block 3 included 40 stimuli. All sequences of stimuli/cues were randomized (www.randomizer.org), except that more than two consecutive presentations of the same stimuli were avoided.

Testees are asked to follow instructions, give their responses aloud (vocal response) and do the task as fast as possible avoiding mistakes. The examiner writes down the answers on answers sheets and records the time testees take to complete each block.

Scores are the RCS per block, determined as the number of correct classifications of stimuli divided by the time taken to complete each block, in seconds. Shifting cost was the RCS of block 3 minus the RCS of blocks 1 and 2 (sum of correct answers divided by the sum of times). Note that Reimers and Maylor (2005) showed that general switch costs (performance differences between shifting blocks and control blocks), as proposed here, is more sensitive to age effects than specific switch cost measures (difference between shifting and non-shifting trials when both are included in the same block).

Category Switch task [based on Friedman and Miyake (2004), who adapted the task of Mayr and Kliegl (2000); see also Friedman et al. (2006, 2008)]: This task assesses executive shifting and was altered in some respects from the original tasks. Switching costs are generalizable to stimuli such as words, numbers and pictures (Kray and Lindenberger, 2000), so we used pictures instead of words as stimuli to avoid biases possible due to differential reading skills.

Sixteen black and white static pictures that represent concrete nouns were used. These pictures were obtained from the work of Cycowicz et al. (1997). They were standardized for use in Brazil regarding familiarity and naming consistency and were easily recognized by young children (see Pompéia et al., 2001; for other free pictures see Bonin et al., 2020). Four of the images refer to large inanimate objects (bed, refrigerator, house and car); four to small inanimate objects (key, ring, fork, and whistle); four to large living things (elephant, horse, giraffe and lion); and four to small living entities (butterfly, ladybird, ant and frog). Thus, each picture can be classified in terms of two independent semantic categories or dimensions: size and living/nonliving entities. These pictures are presented individually on slides (sizes ranging from 7 to 9 cm in height and 4 to 8 cm in width) in portrait orientation.

The task involves three blocks (the first two are control blocks, the last, the executive block). In block 1, participants are asked to classify pictures into living (“alive”) or non-living (or “dead”, a response that was found to be completely intuitive for testees) entities. In block 2, pictures must be classified into representing things that are big or small in real life, having as a reference of size a real soccer ball. In block 3, testees must switch, that is, categorize the first picture as “alive of dead”, the second as “big or small”, the third as “alive or dead” and so forth. Unlike the original version of the task and the Color Shape task, described above, there are cues to guide classification of stimuli. Testees must keep this order in mind. The presence or absence of external lead to different shifting costs (see Koch, 2003; Li et al., 2019). We therefore believed we could obtain a more robust latent variable by using one shifting task with and another without cues.

All blocks are preceded by a practice trial that includes four pictures that were not used in the test blocks: gorilla, spider, cloths peg and train. In blocks 1 and 2, 20 pictures are shown; in block 3 there are 40 pictures. The randomization and number of stimuli per block were the same as those in the Color Shape task.

Testees are asked to follow instructions, give their responses aloud (vocal response) and do the task as fast as possible avoiding mistakes. The examiner writes down the answers on answers sheets and records the time testees take to complete each block.

Scores are the RCS per block. For block 1 and 2 the RCS was determined as the number of correct classifications of stimuli divided by the time taken to complete each block, in seconds, as done in the Color Shape task. For block 3, unlike the Color Shape task, the RCS was determined as the number of correct classifications of stimuli when the testees correctly shifting the categorization, divided by the time taken to complete this block. Shifting cost was the RCS of block 3 minus the RCS of blocks 1 and 2 (sum of correct answers divided by the sum of times in both blocks).

Updating tasks

Number Memory [adapted from Letter Memory task; Miyake et al. (2000); Friedman et al. (2006, 2008); St Clair-Thompson and Gathercole (2006)]: This tasks measures executive updating. The original task used letters as stimuli. To avoid difficulty in those who had inadequate or low schooling we chose instead to use numbers from 1 to 9 (as done by Xu et al., 2013, who named the task “Running memory”), considering that numbers are learned earlier and do not depend on schooling to the same extent as letters (Izard et al., 2009; Rasmussen and Bisanz, 2011; Fernández and Abe, 2018).

Digits are printed individually on the center of portrait oriented slides, in black Calibri font, size 54. Testees must recall the last three numbers presented as the task progresses, updating this information as each new number is seen on the next pages/slides. To ensure that participants continuously update the numbers held in working memory, they are asked to speak the numbers aloud (vocal response), updating the numbers to the last three (triads) (Miyake et al., 2000; Friedman et al., 2008), respecting serial position. For example, if the sequence of numbers is: "8-9-2-5-4", they must say "8 ... 89 .... 892 .... 925 .... 254”. In this example, only recall of the last two triads count as updating opportunities. In order to have a more reliable updating measure, the fixed time of presentation proposed in Miyake et al. (2000), Friedman et al.’s (2006, 2008) and Xu et al.’s (2013) studies was removed, as suggested by McMillan et al. (2007), so that the task could be self-paced. Testees are asked to say the word "blank" or something similar if they do not remember a certain number in each triad. This way, if they forget a given digit, its serial position can be established when they pass to the next updating opportunity.

The order of digits is randomized (www.randomizer.org/) with no repetitions of the same numbers in each sequence. The first practice trial included sequential number (12345…) so that the concept of updating can be easily understood. The next practice trial has 9 digits in mixed order. There are then three more practice trials with digits in random order: one sequences of each size with 5, 7 and 9 numbers which equals 2, 4 e 6 updating opportunities, respectively. On the instructions page there is also a visual outline of the task to make it easier to understand. The task itself consists of two blocks with three trials of sequences with 5, 7 and 9 digits randomly ordered (Friedman et al., 2006, 2008).

Unlike the common form of scoring the classic Letter Memory updating task, which only takes into account the correct number of letters recalled when each trial ends, we counted all successful updatings (recall of all three numbers in each triad in the same serial order as presented) in the whole task (maximum of 24). Consequently, testees cannot rely on a strategy of passive maintenance, or recency criterion to respond by waiting until the end of each trial to give a response (see Carriedo et al., 2016).

Testees are asked to follow instructions, give their responses aloud (vocal response) and do the task as fast as possible avoiding mistakes. The examiner writes down the answers on answers sheets and records the time testees take to complete each trial.

The updating RCS was calculated as the total number of triads recalled correctly in the right serial order for all trials in both test blocks (Friedman et al., 2006, 2008; St Clair-Thompson and Gathercole, 2006; McMillan et al., 2007) (maximum of 24 updatings) divided by the total sum of time in seconds taken to complete all trials in both blocks.

Spatial 2-Back task [adapted from Friedman et al. (2008) and Vuontela et al. (2003)]: This task measure executive updating. On each landscape oriented slide there are 10 outlines (in black) of squares measuring 1.6x1.6 cm in the same spatial locations on a white background. On each slide, one of these squares is totally black, giving the impression that it lit up (target squares) as the testees pass from slide to slides. The testees must observe the position of these black squares and say if the position of the black square they see onscreen is in the same or a different spatial location as the black square that “lit up” two slides back. In the original task the rate of presentation is fixed. Here, the task is self-paced, as suggested by McMillan et al. (2007); see also Lawlor-Savage and Goghari (2016). The order of the position of target squares was randomized (www.randomizer.org/) with the exception that the target square locations is not the same in two consecutive slides nor corresponds to the position of targets 3- or 1-back (Friedman et al., 2008).

The task is composed of two practice blocks (one with 12 and one with 24 screens, one quarter of which involve “same” answers). The test trials contain 3 blocks with 24 screens each, each of which containing six “same” answers. For each screen after the second one, answers can be "same” (position) or “different” (position). Each block therefore involves 22 updating opportunities because no answer is required for the first two screens in which target stimuli cannot be compared with the penultimate screens. To remind testees of this, the first two slides of each trial, including practice trials, are numbered in the upper left corner to indicate that no answer is required. If the participant does not remember where the target square was they can give responses (such as "blank") to indicate omission errors, counted as errors (Friedman et al., 2008; Ecker, 2014).

Testees are asked to follow instructions, give their responses aloud (vocal response) and do the task as fast as possible avoiding mistakes. The examiner writes down the answers on answers sheets and records the time testees take to complete each block.

The score is the RCS calculated by dividing the total number of correct updatings (one updating equals correct recall of the three numbers in a triad in the same serial position as presented) by the total sum of time in seconds taken to complete all test blocks.

2. **Regarding Potential Scale Reduction (PSR) and sample size for the Confirmatory Factor Analysis (CFA)**

Table 3S shows the PSR of the CFA. Only 8500 iterations for convergence of the model below 1.1 were necessary and, still, PSR remains close to 1 (see Asparouhov and Muthén, 2010).

To investigate the adequacy of the sample size (N=146) used in the Confirmatory Factor Analysis (CFA) described in Figure 4 in the main document we conducted, post hoc, a Monte Carlo simulation analysis based on the obtained estimates (e.g., items factor loadings and residual variances). The main parameters of interest were the factor loadings. The Monte Carlo simulation considered maximum likelihood estimator based on 10.000 replications and the following criteria, as described by Muthén and Muthén (2002), for the evaluation of the adequacy of the sample size: 1) bias of the parameters and their standard errors should ideally be close to or not exceed 10% for all parameter in the model (see Brown, 2015, page 390); 2) the proportion of replications for which the 95% confidence interval contains the true population parameter value (95% coverage) should be between 0.91 and 0.98; and 3) the power (% significance coefficient) for each parameter should be close to or superior to 0.80 (Cohen, 1988).

As it can be seen in Table 4S, the sample size used here was adequate in general terms. Bias of the parameters and their standard errors did not highly exceed 10%, the 95% coverage value was within 0.93 and 0.98 and the power for each parameter was close to or superior to 0.80, except that the power for the factor loadings underlying the inhibition latent factor loading were much lower than expected (0.54 and 0.41), given their low factor loadings (see Figure 4 in the main document), and that the bias of the parameters for the indicator Stroop Victoria slightly exceeded 10%. However, this does not invalidate our model as not all of the three criteria used here must be met for all parameters; instead, the extent of the deviation from the ideal metrics must be considered in general terms to describe sample size adequacy for a model of interest (Muthén and Muthén, 2002). As explained in the main document, the fact that inhibition cost scores formed a weaker latent factor is not surprising as prior work has found that inhibition of prepotent responses matures after 15 years of age, the maximum limit of our sample (see Xu et al., 2013; Huizinga et al., 2006; Tamnes et al., 2010; Poon, 2018; Theodoraki et al., 2019).

References

Asparouhov, T. & Muthén, B. (2010). Bayesian analysis of latent variable models using Mplus. Technical report. www.statmodel.com/download/BayesAdvantages18.pdf.

Bonin, P., Poulin-Charronnat, B., Lukowski Duplessy, H., Bard, P., Vinter, A., Ferrand, L., et al. (2020). IMABASE: A new set of 313 colourised line drawings standardised in French for name

agreement, image agreement, conceptual familiarity, age-of-acquisition, and imageability. Quarterly Journal of Experimental Psychology (in press). doi: 10.1177/1747021820932822

Brown, T. A. (2015). Confirmatory Factor Analysis for Applied Research, 2nd ed. (New York, NY: Guilford Publications).462p.

Carriedo, N., Corral, A., Montoro, P. R., Herrero, L., and Rucián, M. (2016). Development of the updating executive function: from 7-year-olds to young adults. Developmental Psychology. 52(4), 666. doi: 10.1037/dev0000091

Cothran, D. L., and Larsen, R. (2008). Comparison of inhibition in two timed reaction tasks:the color and emotion Stroop tasks. The Journal of Psychology 142(4), 373-385. doi: 10.3200/JRLP.142.4.373-385

Cycowicz, Y. M., Friedman, D., Rothstein, M., and Snodgrass, J. G. (1997). Picture naming by young children: norms for name agreement, familiarity, and visual complexity. Journal of Experimental Child Psychology 65(2), 171-237. doi:10.1006/jecp.1996.2356

Diamond, A. (2013). Executive functions. Annual Review of Psychology 64, 135–168. doi: 10.1146/annurev-psych-113011-143750

Duncan, M. T. (2006). Assessment of normative data of Stroop test performance in a group of elementary school students in Niterói. Jornal Brasileiro de Psiquiatria. 55, 42-48. doi: 10.1590/S0047-20852006000100006

Ecker, U. K., Oberauer, K., and Lewandowsky, S. (2014). Working memory updating involves item-specific removal. Journal of Memory and Language 74, 1-15. doi:10.1016/j.jml.2014.03.006

Fernández, A.L., and Abe, J., (2018). Bias in cross-cultural neuropsychological testing: problems and possible solutions. Culture and Brain. 6, 1–35. doi: 10.1007/s40167-017-0050-2

Friedman, N. P., & Miyake, A. (2004). The relations among inhibition and interference control functions: a latent-variable analysis. Journal of Experimental Psychology General 133(1), 101-135. doi: 10.1037/0096-3445.133.1.101

Friedman, N. P., Miyake, A., Corley, R. P., Young, S. E., Defries, J. C., and Hewitt, J. K. (2006). Not all executive functions are related to intelligence. Psychological Science 17(2), 172-179. doi: 10.1111/j.1467-9280.2006.01681.x

Friedman, N. P., Miyake, A., Young, S. E., Defries, J. C., Corley, R. P., and Hewitt, J. K. (2008). Individual differences in executive functions are almost entirely genetic in origin. Journal of Experimental Psychology General 137(2), 201. doi: 10.1037/0096-3445.137.2.201

Huizinga, M., Dolan, C. V., and van der Molen, M. W. (2006). Age-related change in executive function: Developmental trends and a latent variable analysis. Neuropsychologia 44(11), 2017-2036. doi: 10.1016/j.neuropsychologia.2006.01.010

Izard, V., Sann, C., Spelke, E. S., and Streri, A. (2009). Newborn infants perceive abstract numbers. Proceedings of the National Academy of Sciences 106:25, 10382-10385. doi: 10.1073/pnas.0812142106

Koch, I. (2003). The role of external cues for endogenous advance reconfiguration in task switching. Psychonomic Bulletin & Review 10(2), 488-492. doi: 10.3758/BF03196511

Kramer, H. J., Lagattuta, K. H., and Sayfan, L. (2015). Why is happy–sad more difficult? Focal emotional information impairs inhibitory control in children and adults. Emotion 15(1) 61. doi: 10.1037/emo0000023

Kray, J., and Lindenberger, U. (2000). Adult age differences in task switching. Psychology and Aging 15(1), 126-147. doi: 10.1037/0882-7974.15.1.126

Lagattuta, K. H., Sayfan, L., and Monsour, M. (2011). A new measure for assessing executive function across a wide age range: Children and adults find happy-sad more difficult than day-night. Developmental Science 14(3), 481-489. doi: 10.1111/j.1467-7687.2010.00994.x

Lawlor-Savage, L., and Goghari, V. M. (2016). Dual n-back working memory training in healthy adults: A randomized comparison to processing speed training. PloS one 11(4), e0151817. doi: 10.1371/journal.pone.0151817

Li, B., Li, X., Stoet, G.,and Lages, M. (2019). Exploring individual differences in task switching. Acta Psychologica 193, 80-95. doi: 10.1016/j.actpsy.2018.12.010

Mayr, U., and Kliegl, R. (2000). Task-set switching and long-term memory retrieval. Journal of Experimental Psychology: Learning, Memory, and Cognition. 26(5):1124-40. doi: 10.1037//0278-7393.26.5.1124

McMillan, K. M., Laird, A. R., Witt, S. T., and Meyerand, M. E. (2007). Self-paced working memory: validation of verbal variations of the n-back paradigm. Brain Research 1139, 133-142. doi: 10.1016/j.brainres.2006.12.058

Miyake, A., Friedman, N. P., Emerson, M. J., Witzki, A. H., Howerter, A., and Wager, T. D. (2000). The unity and diversity of executive functions and their contributions to complex "frontal lobe" tasks: A latent variable analysis. Cognitive Psychology 41, 49-100. doi:10.1006/cogp.1999.0734

Miyake, A., Emerson, M. J., Padilla, F., and Ahn, J. C. (2004). Inner speech as a retrieval aid for task goals: The effects of cue type and articulatory suppression in the random task cuing paradigm. Acta Psychologica 115(2-3), 123-142. doi: 10.1016/j.actpsy.2003.12.004

Muthén, L. K., and Muthén, B. (2002). How to use a Monte Carlo study to decide on sample size and determine power. Structural equation modeling, 9(4), 599-620. doi: 10.1207/S15328007SEM0904_8

Pompéia, S., Miranda, M. C., and Bueno, O. F. A. (2001). A set of 400 pictures standardized for Portuguese: norms for name agreement, familiarity and visual complexity for children and adults. Arquivos de Neuro-psiquiatria 59(2B), 330-337. doi:10.1590/S0004-282X2001000300004

Poon, K. (2018). Hot and cool executive functions in adolescence: development and contributions to important developmental outcomes. Frontiers in psychology 8, 2311. doi: 10.3389/fpsyg.2017.02311

Rasmussen, C., and Bisanz, J. (2011). The relation between mathematics and working memory in young children with fetal alcohol spectrum disorders. The Journal of Special Education 45(3), 184-191. doi: 10.1177/0022466909356110

Reimers, S., and Maylor, E. A. (2005). Task switching across the life span: effects of age on general and specific switch costs. Developmental Psychology 41(4), 661. doi: 10.1037/0012-1649.41.4.661

Spreen, O., and Strauss, E. (1998). “Executive Functions”, in A compendium of neurological tests: administration, norms, and commentary, eds. O. Spreen, and E. Strauss (New York, NY: Oxford University Press), 171-231.

St Clair-Thompson, H. L., and Gathercole, S. E. (2006). Executive functions and achievements in school: Shifting, updating, inhibition, and working memory. Quarterly Journal of Experimental Psychology 59(4), 745-759. doi: 10.1080/17470210500162854

Strauss, E., Sherman, E. M., and Spreen, O. (2006). “Executive Functions”, in A compendium of neuropsychological tests: administration, norms, and commentary, E. Strauss, E. M. Sherman, and O. Spreen (New York, NY: Oxford University Press,Third Edition) (401-545).

Tamnes, C. K., Ostby, Y., Walhovd, K. B., Westlye, L. T., Due-Tonnessen, P., and Fjell, A. M. (2010). Neuroanatomical correlates of executive functions in children and adolescents: a magnetic resonance imaging (MRI) study of cortical thickness. Neuropsychologia 48(9), 2496-2508. doi:10.1016/j.neuropsychologia.2010.04.024

Theodoraki, T. E., McGeown, S. P., Rhodes, S. M., and MacPherson, S. E. (2019). Developmental changes in executive functions during adolescence: A study of inhibition, shifting, and working memory. British Journal of Developmental Psychology 38, 74-89. doi: 10.1111/bjdp.12307

Tottenham, N., Tanaka, J. W., Leon, A. C., McCarry, T., Nurse, M., Hare, T. A., et al. (2009). The NimStim set of facial expressions: judgments from untrained research participants. Psychiatry Research 168(3), 242-249. doi: 10.1016/j.psychres.2008.05.006

Vuontela, V., Steenari, M. R., Carlson, S., Koivisto, J., Fjällberg, M., and Aronen, E. T. (2003). Audiospatial and visuospatial working memory in 6–13 year old school children. Learning & Memory 10(1), 74-81. doi:10.1101/lm.53503

Xu, F., Han, Y., Sabbagh, M. A., Wang, T., Ren, X., and Li, C. (2013). Developmental differences in the structure of executive function in middle childhood and adolescence. PloS one 8(10), e77770, 1-9. doi: 10.1371/journal.pone.0077770

Table 1S. Inter-rater reliability and 95% Confidence Intervals (CI) based on scoring of four examiners of raw scores of 10% of the sample (N=15) in all blocks per tasks according to each executive domain.

**Tasks/Blocks**	**Accuracy ICC(±95%CI)**	**Speed ICC(±95%CI)**
INHIBITION TASKS		
Stroop Victoria – Block 1 (control: name color patches)	1.000 (1.000-1.000)	0.999 (0.998- 1.000)
Stroop Victoria – Block 2 (executive: name ink of color names)	1.000 (1.000-1.000)	0.999 (0.998- 1.000)
Stroop Happy Sad – Block 1 (control: name emotion)	1.000 (1.000-1.000)	0.982 (0.960- 0.993)
Stroop Happy Sad – Block 2 (executive: name opposite emotion)	0.996 (0.990-0.998)	0.942 (0.872- 0.978)
SHIFTING TASKS		
Color Shape – Block 1 (control: classify by shape)	1.000 (1.000-1.000)	0.934 (0.852- 0.976)
Color Shape – Block 2 (control: classify by color)	1.000 (1.000- 1000)	0.948 (0.882- 0.981)
Color Shape – Block 3 (executive: switch classification)	1.000 (1.000-1.000)	0.973 (0.938- 0.990)
Category switch – Block 1 (control: classify as living/non-living)	1.000 (1.000-1.000)	1.000 (0.999- 1.000)
Category switch – Block 2 (control: classify as big/small)	1.000 (1.000-1.000)	1.000 (1.000- 1.000)
Category switch – Block 3 (executive: switch classification)	1.000 (1.000-1.000)	0.986 (0.969- 0.995)
UPDATING TASKS		
Number Memory – (Total score)	0.999 (0.998-1.000)	1.000 (1.000-1.000)
2-Back – (Total score)	1.000 (0.999-1.000)	0.997 (0.992-0.999)

Table 2S. Pearson correlations (r) of Rate Correct Scores per task block and measures of verbal (Vocabulary) and non-verbal (Block Design) intelligence.

**Measures**	**1**	**2**	**3**	**4**	**5**	**6**	**7**	**8**	**9**	**10**	**11**	**12**	**13**	**14**	**15**	**16**	**17**
**Inhibition**																	
1. Stroop Victoria – Block 1 (control: name color patches)	-																
2. Stroop Victoria – Block 2 (executive: name ink of color names)	*0.56	-															
3. Stroop Victoria – Inhibition cost	*-0.64	*0.28	-														
4. Stroop Happy Sad – Block 1 (control: name emotion)	*0.61	*0.49	*-0.26	-													
5. Stroop Happy Sad – Block 2 (executive: name opposite emotion)	*0.44	*0.48	-0.07	* 0.60	-												
6. Stroop Happy Sad – Inhibition cost	*-0.30	-0.12	*0.24	*-0.62	*0.26	-											
**Shifting**																	
7. Color Shape – Block 1 (control: classify by shape)	*0.54	*0.36	*-0.29	*0.48	*0.32	*-0.27	-										
8. Color Shape – Block 2 (control: classify by color)	*0.55	*0.44	*-0.23	*0.54	*0.42	*-0.23	*0.81	-									
9. Color Shape – Block 3 (executive: switch classification)	*0.57	*0.61	-0.10	*0.58	*0.50	*-0.22	*0.55	*0.61	-								
10. Color Shape – Shifting cost	*-0.34	-0.14	*0.27	*-0.29	*-0.17	*0.19	*-0.84	*-0.79	-0.10	-							
11. Category switch – Block 1 (control: classify as living/non-living)	*0.41	*0.42	-0.08	*0.45	*0.37	*-0.18	*0.58	*0.61	*0.60	*-0.38	-						
12. Category switch – Block 2 (control: classify as big/small)	*0.42	*0.45	-0.07	*0.41	*0.44	-0.06	*0.56	*0.53	*0.55	*-0.34	*0.68	-					
13. Category switch – Block 3 (executive: switch classification)	*0.51	*0.50	-0.12	*0.43	*0.50	-0.03	*0.35	*0.37	*0.62	-0.07	*0.48	*0.57	-				
14. Category switch – Shifting cost	-0.10	-0.13	-0.00	*-0.19	-0.11	0.12	*-0.44	*-0.43	*-0.22	*0.41	*-0.68	*-0.61	*0.16	-			
**Updating**																	
15. Number Memory (Total score)	*0.35	*0.48	0.04	*0.43	*0.41	-0.11	*0.30	*0.33	*0.48	-0.10	*0.37	*0.44	*0.57	-0.05	-		
16. 2-Back (Total score)	*0.40	*0.28	*-0.20	*0.21	*0.25	-0.00	*0.49	*0.38	*0.48	*-0.26	*0.38	*0.46	*0.48	-0.14	*0.30	-	
**Intelligence measures**																	
17. Vocabulary (raw score)	*0.31	*0.39	0.00	*0.26	*0.27	-0.05	0.14	*0.25	*0.41	0.02	*0.22	*0.36	*0.39	-0.06	*0.40	*0.19	-
18. Block Design (raw score)	*0.37	*0.45	-0.01	*0.30	*0.35	-0.02	*0.21	*0.21	*0.39	-0.02	*0.24	*0.40	*0.47	-0.02	*0.45	*0.32	*0.46

Note. N: 146; *$p < 0.05$

Table 3S: Potential Scale Reduction (PSR) per iteration regarding the Confirmatory Factor Analysis used to replicate the three correlated factor model of Miyake et al. (2000) with the present sample (N=146).

Iteration	Potencial Scale reduction
100	3,605
200	1,503
300	1,960
400	2,448
500	1,967
600	2,172
700	1,683
800	1,562
900	1,754
1000	2,126
1100	2,088
1200	2,784
1300	3,612
1400	4,081
1500	4,204
1600	3,671
1700	3,736
1800	3,738
1900	3,730
2000	3,519
2100	2,548
2200	2,177
2300	2,019
2400	1,738
2500	1,588
2600	1,582
2700	1,603
2800	1,590
2900	1,512
3000	1,435
3100	1,378
3200	1,362
3300	1,322
3400	1,283
3500	1,267
3600	1,293
3700	1,325
3800	1,376
3900	1,436
4000	1,505
4100	1,573
4200	1,666
4300	1,772
4400	1,838
4500	1,919
4600	1,980
4700	2,024
4800	1,931
4900	1,816
5000	1,705
5100	1,692
5200	1,735
5300	1,664
5400	1,682
5500	1,684

5600	1,764
5700	1,843
5800	1,928
5900	2,022
6000	2,037
6100	1,871
6200	1,744
6300	1,615
6400	1,522
6500	1,447
6600	1,402
6700	1,356
6800	1,306
6900	1,271
7000	1,246
7100	1,223
7200	1,196
7300	1,198
7400	1,189
7500	1,183
7600	1,180
7700	1,172
7800	1,172
7900	1,174
8000	1,164
8100	1,158
8200	1,126
8300	1,109
8400	1,094
8500	1,083

Table 4S: Monte Carlo simulation results for the Confirmatory Factor Analysis used to confirm the model by Miyake et al. (2000).

Indicators	% parameter bias	95% coverage	% sig. coeff.
Shifting			
Color Shape	-8.291	0.943	0.914
Category Switch	0.563	0.970	0.887
Inhibiton			
Stroop Victoria	-11.473	0.987	0.542
Stroop Happy Sad	-6.269	0.980	0.471
Updating			
Number memory	-1.600	0.977	0.721
2-Back	-9.900	0.938	0.784

N.B. population = 95% cover = the proportion of replications for which the 95% confidence interval contains the true population parameter value; %sig. coeff.= proportion of replications in which the parameter is significantly different from zero at the .05 alpha level (power).
