# Supplementary material for: An Adaptable, Open-Access Test Battery to Study the Fractionation of Executive-Functions in Diverse Populations
Source: Front Psychol. 2021 Mar 30;12:627219. doi: 10.3389/fpsyg.2021.627219 (PMC8042159; doi:10.3389/fpsyg.2021.627219)
Supplement: Supplementary file 2 [file Data_Sheet_2.pdf]

frontiers

+Supplementary Material II

Administration and correction Manual of the Free Research Executive Evaluation (FREE) test battery (English version)

This is an administration and correction manual, with answer sheets, for an open access test battery for research purposes that can be used to assess the fractionation (or unity and diversity) of executive functions. The manual describes how to administer and correct two tasks of each of the executive inhibition, switching and updating domains.

For the rationale underlying the selection of these tasks, stimuli and response mode please refer to the main document "**An adaptable, open-access test battery to study the fractionation of executive-functions in diverse populations**" to which this manual is attached. The tasks themselves are in pptx or pdf format in English and Portuguese.

Users from countries or cultures which are non-English and non-Brazilian Portuguese speaking must adapt the instructions and stimuli for their own context following the suggestions in the detailed description of the tasks in the main document and the Supplementary Material I.

This material is registered under an Attributions ShareAlike Creative Commons License (CC BY-SA) which means anyone can use and modify its content as long as the authors are credited. In more detail:

- Attribution (by): Requires users of the tasks proposed here to give the authors of the original manual credit (in other words, cite this work). This does not mean we endorse the use in other works or publications.

- ShareAlike (sa): Allows other to copy, distribute, display, perform, and modify the original tasks, correction modes, etc., as long as they distribute any modified work on the same terms (CC BY-SA). If the material is altered, these changes must be clearly indicated. If users want to distribute modified works under other terms, they must get our permission first.

CC BY SA

Considerations for test usage

Researchers interested in using this test battery must consider the following after having read the rationale for choice of tasks and understood their characteristics, task instructions, administration and correction procedures:

1. The instructions and stimuli must be adapted for each population under study taking into account how easily they are identified and, if possible, the length (number of syllables, phonemes) of vocal responses. If norms for picture naming, for instance, are not available, pilot studies must be conducted to make sure the populations under study easily identify them and understand how they are to be used (e.g. understand what is a living versus non-living entity; be from a culture in which happy is automatically regarded as "opposite" to sad; know the real size of the represented stimuli; have automatized labels for the colors that are used). The same must be done regarding the clarity of instructions.

2. The tasks described here were not developed for diagnosis of cognitive difficulties. If the objective is to characterize a clinical group, a control group must be included with similar characteristics to those of the population under investigation.

3. If testees' performance are to be assessed at two different times, other version of each task can be built by alternating the order of the trials in each block of the task and balancing test versions among participants and groups. Performance in one of the two tasks proposed for one executive domains should not be compared directly with performance on the other task at another testing time because performance will vary according to the task, even though both measure the same construct. Also consider learning effects, which diminish the use of executive functions when people practice executive tasks.

4. If various tasks are used in different participants, consider varying the order of presentation between participants to minimize effects of fatigue.

5. Here we propose the use of Rate Correct Scores (RCS) - the number of correct answers divided by the time taken to finish the task blocks. This type of metric has the advantage that it takes into account speed-accuracy trade-offs. However, number of errors, correct answers and/or time taken to complete tasks, trials or blocks can be used, although these have psychometric disadvantages. Likewise, we used absolute executive costs (performance in executive blocks minus that in control blocks or vice versa), but relative costs (performance in executive blocks divided by performance in control blocks or vice versa) can be used instead.

6. The test battery was developed to allow the obtention of latent variables from the scores of the two tasks of each domain. Notwithstanding, individual scores in each task can also be used.

STROOP VICTORIA - TEST ADMINISTRATION AND SCORING

GENERAL INSTRUCTIONS

Which executive domain is assessed by this task: This task assesses inhibition of prepotent/automatic responses; in this particular case, the ability to inhibit the automatic tendency to name written words and, instead, name colors.

What the testees are required to do: In this task the testees are asked to name aloud the ink color of stimuli presented on a single page, from left to right. The tasks must be done as quickly as possible avoiding mistakes. Instructions can be read by the testee or be read to them. The testees can be allowed to use their fingers to follow each line while performing the task, as this helps some individuals not to lose track of the stimuli. Self-corrections are allowed as long as they occur before the answer to the next stimulus.

What the task entails: This task includes three blocks. In this task, testees can see what the target stimuli look like during instructions, but there are no practice trials. In all blocks, testees must name the colors they see (and not read words aloud when words are present). In block 1, color patches are present in rectangles. In block 2, the stimuli are words that are not color names printed in different colored ink. In block 3, the stimuli are words that are color names printed in ink colors that are incongruent with the written words (e.g. the word "pink" written in blue ink).

Answers are always vocal. The task is self-paced and the testees passes from instructions to the tasks itself by swiping on the screen, pressing a button on the mouse or a key in the computer, or by turning pages, depending on the apparatus or mode of application used in a particular experiment. Testees are, however, not allowed to return to screens/pages that have been already seen. The objective is to complete each block as quickly as possible with the highest possible accuracy.

Which testee characteristics preclude the use of this task to assess executive functions: This task may not assess executive functioning in test takers who are visually impaired or whose vision is not corrected, who have known/diagnosed language or speech disorders or such like that the examiner deems can interfere with performance, nor in testees who have difficulty in discriminating colors and who cannot read with relative fluency.

What the examiner does during the task:

1. Recording speed: The examiner must mark how long the testee takes to complete each block in seconds, from the appearance of the first stimulus until the answer to the last stimulus. There is a page that precedes the beginning of each block that serves to help establish when the task will begin. As soon as the testee names the color of the last stimulus, the stopwatch must be stopped. First, the answer to the last stimulus must be written down. Only then the examiner must look at the stopwatch and mark the time in seconds in the light gray cells on the answer sheet that correspond to each block.

2. Recording accuracy: On the answer sheet the examiner should use tick marks (✓) to indicate the correct answers; **"X"** to indicate errors or skipped stimuli; and **"?"** for ambiguous answers, or in cases in which the examiner fails to take note of the answers. These markings should be made on each color name on the answer sheet. If testees self-correct answers, the examiner must mark the last given answer for each stimulus made prior to the response to the next stimulus. Self-corrections that occur after should not be considered.

What is crucial for adequate test application:

• The task must not be administered before the examiner practices taking down answers following all the instructions in this manual until this becomes fairly automatic.

• The examiner must be attentive to any possible naming difficulties and make sure that testees can see the stimuli and that they can read with relative fluency. This can be checked by asking testees to read part of the instructions. Testees must also report not having difficulties with color discriminations.

TEST ADMINISTRATION DETAILS

• The examiner must sit next to the testee so he/she can also see the screens or pages.

• The examiner must ask the testee for permission to audio record the session because it is difficult to keep track of answers. This allows the examiner to hear the answers again if they have difficulty marking responses on the answer sheet.

• The examiner should ask the testee to read the instructions or, if the testees prefer, instructions can be read to them. The examiner must make sure the testee understood the instructions before commencing the task. When in doubt, the instructions should be repeated. The testee must be prepared to start naming colors as quickly as possible before passing to the screen that includes all stimuli.

• The examiner should start the stopwatch with the non-dominant hand as soon as the first stimulus is visible and turn it off immediately after the last answer was given in each block. Before looking at the stopwatch the examiner must write down the answer to the last stimulus. Only then they should look at the stopwatch and write down the time it took the testee to complete each block (in seconds) on the answer sheet.

• Testees must be allowed to rest between blocks. The examiner should use common sense to determine how long the testee can rest between blocks.

• The testee must complete all blocks in full. There are no interruption criteria for this task.

HOW TO TIME HOW LONG IT TAKES TESTEES TO COMPLETE EACH BLOCK

• The examiner must mark how long the testee takes to complete each block in seconds, from the appearance of the first stimulus until the answer to the last stimulus. There is a page that precedes the beginning of each block that serves to help establish when the task will begin. As soon as the testee classifies the last stimulus, the stopwatch must be stopped. First, the answer to the last stimulus must be written down. Only then should the examiner look at the stopwatch and mark the time in seconds in the answer sheet in the cell that correspond to each condition.

HOW TO MARK SCORES

1. Upon completion of the task, the examiner must count and write down the total number of correct answers in the corresponding cells for each block, in light gray on the right of the answer sheet, remembering that each correct answer is equivalent to one point. The light gray number “(24)” in the cells indicates the maximum possible number of correct responses.

2. The examiner can then calculate the absolute inhibition costs (time taken to complete block 3 minus time taken to complete block 1 and or 2). The same should be done for correct answers. Relative inhibition costs can be calculated by using division instead of subtraction (i.e. performance in block 3/block 1). The results must be written down in the last cell at the bottom of the answer sheet (in dark gray). An alternative scoring system is Rate Correct Score (RCS), or the total number of correct answers per block (or inhibition cost of correct answers) divided by the total time taken to complete the block (or inhibition cost in time).

Be attentive to any unexpected events

• If there are many sequential errors it is likely that the testee skipped a stimulus and/or that the examiner failed to notice one or more of the testees ’answers or self-corrections. The audio recording should be checked.

• Very low scores should only be interpreted as executive difficulties when the examiner believes there are no other perceptual or cognitive deficits which could explain the results (check testee characteristics that preclude the use of this task). In these cases, a referral to the professional that can confirm a possible diagnosis should be provided. If testees are minors, their teacher and/or guardians should be contacted when there are any clinical or cognitive suspicions that can justify testees ’unusual performance. Alternatively, it may be that the testee is unwilling to do the task or to follow instructions. Use common sense to determine whether scores actually reflect the testees ’executive abilities.

• Use the space at the bottom of the sheet to record incidents that are considered unusual or unexpected. At times, it is possible only in retrospect to make sense of things that take place during cognitive testing.

MATERIAL: 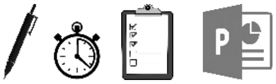 or 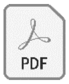

STROOP VICTORIA – ANSWER SHEET

Block 1: Rectangles				Time (s)	Correct (no.)
green	pink	blue	black		
pink	black	green	blue		
blue	green	pink	black		
black	pink	blue	green		
pink	blue	green	black		
black	green	blue	pink		(24)

block 2: words that are not color names					
green	pink	blue	black		
pink	black	green	blue		
blue	green	pink	black		
black	pink	blue	green		
pink	blue	green	black		
black	green	blue	pink		(24)

block 3: words that are color names					
green	pink	blue	black		
pink	black	green	blue		
blue	green	pink	black		
black	pink	blue	green		
pink	blue	green	black		
black	green	blue	pink		(24)

	Time (Block 3-1) (s)	Correct (Block 3-1) no.
**Inhibition cost scores**		

Notes:

STROOP VICTORIA – ANSWER SHEET WITH EXAMPLE OF CORRECTIONS

Block 1: Rectangles

				Time (s)	Correct (no.)
green ✓	pink ✓	blue ✓	black ✓	24	24 (24)
pink ✓	black ✓	green ✓	blue ✓		
blue ✓	green ✓	pink ✓	black ✓		
black ✓	pink ✓	blue ✓	green ✓		
pink ✓	blue ✓	green ✓	black ✓		
black ✓	green ✓	blue ✓	pink ✓		

block 2: words that are not color names

				Time (s)	Correct (no.)
green ✓	pink ✓	blue ✓	black ✓	28	23 (24)
pink ✓	black ✓	green ✓	blue ✓		
blue ✓	green ✓	pink ✗	black ✓		
black ✓	pink ✓	blue ✓	green ✓		
pink ✓	blue ✓	green ✓	black ✓		
black ✓	green ✓	blue ✓	pink ✓		

block 3: words that are color names

				Time (s)	Correct (no.)
green ✓	pink ✓	blue ✓	black ✓	32	21.91 (24)
pink ✓	black ✓	green ✓	blue ✗		
blue ✗	green ✓	pink ✓	black ✓		
black ✓	pink ✓	blue ✓	green ✓		
pink ✓	blue ✓	green ✓	black ✓		
black ✓	green ?	blue ✓	pink ✓		

	Time (Block 3-1) (s)	Correct (Block 3-1) no.
Inhibition cost scores	8	-2.09

Notes:

Details:

- ✓ Correct answers.
- ✗ Inhibition error (e.g. saying “blue” when the answer is “green”) or when the testee skips a stimulus.
- ? Missing data due to ambiguous responses, failure to write response, incomprehensible notes, etc. We suggest that, if these cases do not exceed 10% of the stimuli in each block, the rule of three* be used to estimate the total number of correct responses. When loss of data exceeds 10%, examiners must decide whether to use responses or not.
- Costs illustrated here are absolute costs considering blocks 3 and 1.

* Rule of three (example for this case of block 3):

21 (correct answers) - 23 (total number of items answered - item marked with “?” Is not considered)

x (hits) - 24 (total number items)

x = 21.91 (use two digits after the decimal point, rounding decimals to the nearest hundredths)

HAPPY SAD STROOP TASK - TEST ADMINISTRATION AND SCORING

GENERAL INSTRUCTIONS

Which executive domain is assessed by this task: This task assesses the ability to inhibit automatic/prepotent responses; in this particular case, the automatic tendency to name facial expressions and, instead, name another (“opposite”) emotion.

What the testees are required to do: In this task the testees are asked to name aloud emotions of facial stimuli, from left to right. The tasks must be done as quickly as possible avoiding mistakes. Instructions can be read by the testee or be read to them. The testees can be allowed to use their fingers to follow each line while performing the task, as this helps some individuals not to lose track of the stimuli. Self-corrections are allowed as long as they occur before the answer to the next stimulus.

What the task entails: This task includes three blocks. In this task, testees can see what the target stimuli look like during instructions, but there are no training trials. In block 1, emojis with happy and sad faces are presented. In block 2, the stimuli are the same facial expressions but on black and white photographs. In both these cases, testees are asked to name the emotion of each stimulus. Stimuli in block 3 are the same as those in block 2 but, in this case, testees are required to name the opposite emotions (e.g. say “sad” when they see a happy face.

Answers are always vocal. The task is self-paced and the testees passes from instructions to the tasks itself by swiping on the screen, pressing a button on the mouse or a key in the computer, or by turning pages, depending on the apparatus or mode of application used in a particular experiment. Testees are, however, not allowed to return to screens/pages that have been already seen. The objective is to complete each block as quickly as possible with the highest possible accuracy.

Which testee characteristics preclude the use of this task to assess executive functions: This task may not assess executive functioning in test takers who are visually impaired or whose vision is not corrected, who have known/diagnosed language or speech disorders or such like that the examiner deems can interfere with performance, nor in those who have difficulty in identifying emotions (such as individuals on the autism spectrum).

What the examiner does during the task:

1. Recording speed: The examiner must mark how long the testee takes to complete each block in seconds, from the appearance of the first stimulus until the answer to the last stimulus. There is a page that precedes the beginning of each block that serves to help establish when the task will begin. As soon as the testee names the emotion (or opposite emotion) of the last stimulus, the stopwatch must be stopped. First, the answer to the last stimulus must be written down. Only then the examiner must look at the stopwatch and mark the time in seconds in the light gray cells on the answer sheet that correspond to each block.

2. Recording accuracy: On the answer sheet the examiner should use tick marks (✓) to indicate the correct answers, **"X"** to indicate errors or skipped stimuli, and **"?"** for ambiguous answers, or in cases in which the examiner fails to take note of the answers. These markings should be made on or above each emotion name on the answer sheet. If testees self-correct answers, the examiner must mark the last given answer for each stimulus made prior to the response to the next stimulus. Self-corrections that occur after should not be considered.

What is crucial for adequate test application:

• The task must not be administered before the examiner practices taking down answers following all the instructions in this manual until this becomes fairly automatic.

• The examiner must make sure that testees can see the stimuli, and be attentive to any possible naming, perceptual or emotional identification difficulties.

TEST ADMINISTRATION DETAILS

• The examiner must sit next to the testee so he/she can also see the screens or pages.

• The examiner must ask the testee for permission to audio record the session because it is difficult to keep track of answers. This allows the examiner to hear the answers again if they have difficulty marking responses on the answer sheet (presented next).

• The examiner should ask the testee to read the instructions or, if the testees prefer, instructions can be read to them. The examiner must make sure the testee understood the instructions before commencing the task. When in doubt, the instructions should be repeated. The testee must be prepared to start naming emotions as quickly as possible before passing to the screen that includes all stimuli.

• The examiner should start the stopwatch with the non-dominant hand as soon as the first stimulus is visible and turn it off immediately after the last answer, was given in each block. Before looking at the stopwatch the examiner must write down the answer to the last stimulus. Only then they should look at the stopwatch and write down the time it took the testee to complete each block (in seconds) on the answer sheet.

• Testees must be allowed to rest between blocks. The examiner should use common sense to determine how long the testee can rest between blocks.

• The testee must complete all blocks in full. There are no interruption criteria for this task.

HOW TO TIME HOW LONG IT TAKES TESTEES TO COMPLETE EACH BLOCK

• The examiner must mark how long the testee takes to complete each block in seconds, from the appearance of the first stimulus until the answer to the last stimulus. There is a page that precedes the beginning of each block that serves to help establish when the task will begin. As soon as the testee classifies the last stimulus, the stopwatch must be stopped. First, the answer to the last stimulus must be written down. Only then should the examiner look at the stopwatch and mark the time in seconds in the answer sheet in the cell that correspond to each condition.

HOW TO MARK SCORES

1. Upon completion of the task, the examiner must count and write the total number of correct answers in the corresponding cells for each block (in light gray on the right) on the answer sheet, remembering that each correct answer is equivalent to one point. The light gray number “(20)” in the cells indicates the maximum possible number of correct responses.

2. The examiner can then calculate the absolute inhibition costs (time taken to complete block 3 minus time taken to complete block 1 and or 2; do the same for correct answers). Relative inhibition costs can be calcite by using division instead of subtraction (i.e. performance in block 3/block 1). The results must be written down in the last cell at the bottom of the answer sheet (in dark gray). An alternative scoring system is Rate Correct Score, or the total number of correct answers per block (or inhibition cost of correct answers) divided by the total time taken to complete the block (or inhibition cost in time).

Be attentive to any unexpected events

• If there are many sequential errors it is likely that the testee skipped a stimulus and/or that the examiner failed to notice one or more of the testees ‘answers or self-corrections. The audio recording should be checked.

• Very low scores should only be interpreted as executive difficulties when the examiner believes there are no other perceptual or cognitive deficits which could explain the results (check testee characteristics that preclude the use of this task). In these cases, a referral to the professional that can confirm a possible diagnosis should be provided. If testees are minors, their teacher and/or guardians should be contacted when there are any clinical or cognitive suspicions that can justify testees ’unusual performance. Alternatively, it may be that the testee is unwilling to do the task or to follow instructions. Use common sense to determine whether scores actually reflect the testees ‘executive abilities.

• Use the space at the bottom of the sheet to record incidents that are considered unusual or unexpected. At times, it is possible only in retrospect to make sense of things that take place during cognitive testing.

MATERIAL: 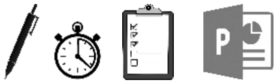 or 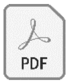

STROOP HAPPY SAD – ANSWER SHEET

Block 1: Emojis					Time (s)	Correct (no.)
sad	happy	sad	happy	sad		
happy	sad	sad	happy	happy		
sad	happy	happy	sad	happy		
sad	happy	sad	happy	sad		(20)

Block 2: Emotion on faces				(w: woman; m: man)	Time (s)	Correct (no.)
sad (w)	happy (m)	happy (w)	sad (m)	sad (w)		
happy (w)	sad (m)	sad (w)	happy (w)	happy (m)		
sad (m)	sad (w)	happy (m)	sad (m)	happy (w)		
happy (m)	happy (w)	sad (w)	happy (m)	sad (m)		(20)

Block 3: Opposite emotions on faces				(w: woman; m: man)	Time (s)	Correct (no.)
happy (m)	sad (m)	happy (w)	sad (m)	sad (w)		
sad (w)	happy (w)	sad (m)	happy (m)	happy (w)		
sad (m)	sad (w)	happy (m)	happy (w)	sad (m)		
happy (m)	happy (w)	sad (w)	happy (m)	sad (w)		(20)

	Time (3-2) (s)	Correct (3-2) no.
**Inhibition cost**		

Notes:

STROOP HAPPY SAD – ANSWER SHEET WITH EXAMPLE OF CORRECTIONS

Block 1: Emojis					Time (s)	Correct (no.)
sad ✓	happy ✓	sad ✓	happy ✓	sad ✓	17	20
happy ✓	sad ✓	sad ✓	happy ✓	happy ✓		
sad ✓	happy ✓	happy ✓	sad ✓	happy ✓		
sad ✓	happy ✓	sad ✓	happy ✓	sad ✓		(20)

Block 2: Emotion on faces				(w: woman; m: man)	Time (s)	Correct (no.)
sad (w) ✓	happy (m) ✓	happy (w) ✓	sad (m) ✓	sad (w) ✓	19	19
happy (w) ✓	sad (m) ✓	sad (w) ✓	happy (w) ✓	happy (m) ✓		
sad (m) ✗	sad (w) ✓	happy (m) ✓	sad (m) ✓	happy (w) ✓		
happy (m) ✓	happy (w) ✓	sad (w) ✓	happy (m) ✓	sad (m) ✓		(20)

Block 3: Opposite emotions on faces				(w: woman; m: man)	Time (s)	Correct (no.)
happy (m) ✓	sad (m) ✓	happy (w) ✓	sad (m) ✓	sad (w) ✓	25	17.89
sad (w) ?	happy (w) ✓	sad (m) ✗	happy (m) ✓	happy (w) ✓		
sad (m) ✓	sad (w) ✓	happy (m) ✓	happy (w) ✗	sad (m) ✓		
happy (m) ✓	happy (w) ✓	sad (w) ✓	happy (m) ✓	sad (w) ✓		(20)

	Time (3-2) (s)	Correct (3-2) no.
Inhibition cost	6	-1.11

Notes:

Details:

- ✓ Correct answers.
- ✗ Inhibition error (for example, in block 1 and 2, saying "happy" for sad face) or when the testee skips a stimulus.
- ? Missing data due to ambiguous responses, failure to write response, incomprehensible notes, etc. We suggest that, if these cases do not exceed 10% of the stimuli in each block, the rule of three* be used to estimate the total number of correct responses. When loss of data exceeds 10%, examiners must decide whether to use responses or not. If this occurs in block 3, executive costs cannot be calculated (they must be considered missing values).
- Costs illustrated here are absolute costs considering blocks 3 and 1.

* Rule of three (example for this case of block 3):
17 (correct answers) - 19 (total number of items answered - item marked with "?" Is not considered)
x (hits) - 20 (total number of items)
x = 17.89 (use two digits after the decimal point, rounding decimals to the nearest hundredths)

COLOR-SHAPE TASK - TEST ADMINISTRATION AND SCORING

GENERAL INSTRUCTIONS

Which executive domain is assessed by this task: This task assesses the ability to shift (or switch) between two tasks; in this particular case, switching the classification of images (stimuli) according to two categories: by color and by shape) following clues.

What the testees are required to do: The testees are required to classify picture stimuli aloud by color, by shape and to alternate between these category classifications following clues that are placed above each stimulus. The tasks must be done as quickly as possible avoiding mistakes. Self-corrections are allowed as long as they occur before the answer to the next stimulus.

What the task entails: This task includes three blocks and is preceded by practice trials. Above all stimuli there are clues that guide classifications (an abstract outline in black or a monochromatic rainbow). In block 1 testees must classify each stimulus by shape (clue: abstract outline in black), in block 2, by color (clue: monochromatic rainbow); in block 3 testees must shift between color and shape classifications following clues, which vary from trial to trial.

Answers are always vocal. The task is self-paced and the testees passes from instructions to the tasks itself and from stimulus to stimulus by swiping on the screen, pressing a button on the mouse or a key in the computer, or by manually turning pages, depending on the apparatus or mode of application used in a particular experiment. Testees are not allowed to return to screens/pages that have been already seen. The objective is to complete each block as quickly as possible with the highest possible accuracy.

Which testee characteristics preclude the use of this task to assess executive functions: This task may not assess executive functioning in test takers who are visually impaired or whose vision is not corrected, who have known/diagnosed language or speech disorders or such like that the examiner deems can interfere with performance, nor in those who are not familiar with names of the colors (black, grey) and shapes (circles and squares).

What the examiner does during the task:

1. Recording speed: The examiner must mark how long the testee takes to complete each block in seconds, from the appearance of the first stimulus until the answer to the last stimulus. There is a page that precedes the beginning of each block that serves to help establish when the task will begin. As soon as the testee classifies the last stimulus, the stopwatch must be stopped. First, the answer to the last stimulus must be written down. Only then the examiner must look at the stopwatch and mark the time in seconds in the light gray cells on the answer sheet that correspond to each block.

2. Recording accuracy: To help the examiner keep track, all screens are numbered and the screen number is displayed on the answer sheet at the far left of the cells, followed by the possible category and classification of stimuli: “CIR” for circles, “SQ” for squares, “BL” for black or “GR” for gray. In block 3, however, next to the stimulus number there are two sets of letters corresponding to the possible categories and classifications (e.g. “SQ GR” for the square that is gray). The examiner should work from the top (left) down on the answer sheet as the task progresses. On this sheet they should use tick marks (✓) to indicate the correct answers, **"X"** to indicate errors or skipped stimuli, and **"?"** for ambiguous answers, or in cases in which the examiner fails to take note of the answers. If testees self-correct answers, the examiner must mark the last given answer for each stimulus made prior to the response to the next stimulus. Self-corrections that occur after should not be considered.

In block 3, when applying the task, disregard bold markings on the letter. Only pay attention to the answer regardless of the clue. Thus, for a stimulus that is a square which is gray (“SQ” “**GR**”), if the testee says “gray” he/she receives a (✓) (correct classification mark) which must be placed on or over the GR. If the answer is "square", a tick mark (✓) should be placed on or above SQ. The answer “circle” should be noted as an **"X"** on or over SQ and the answer “black” as an **"X"** on or over GR. For this example, GR is marked in bold

because it is the correct classification of the stimulus following its clue (rainbow). However, the ability to shift by following the clue is only considered in another scoring metric (correct shiftings; see below).

What is crucial for adequate test application:

• The task must not be administered before the examiner practices taking down answers following all the instructions in this manual until this becomes fairly automatic.

• The examiner must make sure that testees can see the stimuli, can identify the stimuli's colors and shapes and be attentive to any possible naming and perceptual difficulties.

TEST ADMINISTRATION DETAILS

• The examiner must sit next to the testee so he/she can also see the screens or pages.

• The examiner must ask the testee for permission to audio record the session because it is difficult to keep track of answers. This allows the examiner to hear the answers again if they have difficulty marking responses on the answer sheet (presented next).

• The examiner should ask the testee to read the instructions or, if the testees prefer, instructions can be read to them. The examiner must make sure the testee understood the instructions during the practice trials. When in doubt, the practice trials must be repeated until the testee has understood the instructions. Prepare testees to start classifying stimuli as quickly as possible before they pass to the screen that includes the first stimulus.

• Answers that the examiner regards as similar to those required should be counted as valid (e.g. ball instead of circle). In other words, it must not be insisted that testees' use a specific term to refer to one type of stimuli as long as the given answer is understandable.

• The examiner should start the stopwatch with the non-dominant hand as soon as the first stimulus is visible and turn it off immediately after the last answer was given in each block. Before looking at the stopwatch the examiner must write down the answer to the last stimulus. Only then they should look at the stopwatch and write down the time it took the testee to complete each block (in seconds) on the answer sheet.

• Testees must be allowed to rest between blocks. The examiner should use common sense to determine how long the testee can rest between blocks.

• The testee must complete all blocks in full. There are no interruption criteria for this task.

HOW TO TIME HOW LONG IT TAKES TESTEES TO COMPLETE EACH BLOCK

• The examiner must mark how long the testee takes to complete each block in seconds, from the appearance of the first stimulus until the answer to the last stimulus. There is a page that precedes the beginning of each block that serves to help establish when the task will begin. As soon as the testee classifies the last stimulus, the stopwatch must be stopped. First, the answer to the last stimulus must be written down. Only then should the examiner look at the stopwatch and mark the time in seconds in the answer sheet in the cell that correspond to each condition.

HOW TO MARK SCORES

-Block 1 and 2:

1. For blocks 1 and 2, upon completion of the task, the examiner should count the tick marks (✓: correct answers) and write the total number in the corresponding light gray cells below each block. Each correct answer equals one point. Do not consider practice trials. The number [for example "(20)"] in light gray in these cells shows the maximum possible number of answers.

- Block 3:

1. For block 3, upon completion of the task, the examiner must first count the tick marks (✔ : correct answers) regardless of the clue (indicated by bold letters). This will result in the number of correct classifications, which should be noted in the appropriate cell on the answer sheet (light gray cells below block 3). Do not consider practice trials.

<u>- Shifting metrics</u>

1. Calculate the absolute shifting cost measures: the time spent in the shifting block (block 3) minus the sum of the time to complete blocks 1 and 2; write this number in the corresponding dark gray cells at the bottom of the answer sheet. Do the same for correct classifications. Relative shifting cost can also be calculated [e.g. the time spent in the shifting block (block 3) divided by the sum of the time to complete blocks 1 and 2]. An alternative scoring system is Rate Correct Score, or the total number of correct answers per block (or shifting cost of correct answers) divided by the total time taken to complete the block (or shifting cost in time).

2. For block 3 only, another scoring metric should be used that considers whether the testee shifted categories (shape or color) following the cues. This can be done by looking at whether the examiner followed the bold letters, regardless of ✔ or **X**. answers. To facilitate scoring by this criterion, upon completion of the task, circle the shifting errors (when the answers do not match the bold letters, regardless of whether the classification is correct or not). Then count the total correct shifts in block 3 (how many times the examiner followed the clue) and add the result to the last dark gray cell on the answer sheet.

Be attentive to any unexpected events

• If there are many sequential errors it is likely that the testee skipped a stimulus and/or that the examiner failed to notice one or more of the testees' answers or self-corrections. The audio recording should be checked.

• Very low scores should only be interpreted as executive difficulties when the examiner believes there are no other perceptual or cognitive deficits which could explain the results (check testee characteristics that preclude the use of this task). In these cases, a referral to the professional that can confirm a possible diagnosis should be provided. If testees are minors, their teacher and/or guardians should be contacted when there are any clinical or cognitive suspicions that can justify testees 'unusual performance. Alternatively, it may be that the testee is unwilling to do the task or to follow instructions. Use common sense to determine whether scores actually reflect the testees' executive abilities.

• Use the space at the bottom of the sheet to record incidents that are considered unusual or unexpected. At times, it is possible only in retrospect to make sense of things that take place during cognitive testing.

MATERIAL: 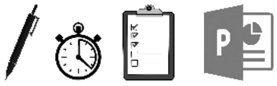 or 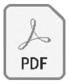

COLOR SHAPE – ANSWER SHEET

PRACTICE (CIR-circle; SQ-square; BL-black; GR-gray)				
1. Shape		2. Color		3. Switching
CIR		BL		**SQ** GR
SQ		GR		CIR **BL**
CIR		BL		SQ **GR**
SQ		GR		**CIR** BL

TEST BLOCKS						
**Block 1 Shape**		**Block 2 Color**		**Block 3 Switching with cue**		
01. **SQ**		21. **GR**		41. CIR **BL**	61. CIR **GR**
02. **CIR**		22. **BL**		42. **SQ** GR	62. **SQ** BL
03. **CIR**		23. **BL**		43. **CIR** BL	63. SQ **BL**
04. **SQ**		24. **GR**		44. SQ **BL**	64. **SQ** GR
05. **SQ**		25. **GR**		45. **CIR** GR	65. **CIR** BL
06. **CIR**		26. **BL**		46. **SQ** BL	66. SQ **BL**
07. **SQ**		27. **GR**		47. SQ **GR**	67. SQ **GR**
08. **SQ**		28. **BL**		48. **CIR** BL	68. **CIR** BL
09. **CIR**		29. **GR**		49. CIR **GR**	69. CIR **GR**
10. **CIR**		30. **GR**		50. **SQ** BL	70. **CIR** BL
11. **SQ**		31. **BL**		51. CIR **BL**	71. CIR **GR**
12. **SQ**		32. **BL**		52. SQ **GR**	72. **SQ** GR
13. **CIR**		33. **GR**		53. **CIR** GR	73. SQ **BL**
14. **SQ**		34. **BL**		54. CIR **BL**	74. CIR **GR**
15. **CIR**		35. **BL**		55. **SQ** GR	75. **SQ** BL
16. **CIR**		36. **GR**		56. CIR **BL**	76. **SQ** GR
17. **SQ**		37. **BL**		57. SQ **BL**	77. CIR **GR**
18. **CIR**		38. **GR**		58. **CIR** GR	78. CIR **BL**
19. **SQ**		39. **BL**		59. SQ **GR**	79. **CIR** GR
20. **CIR**		40. **GR**		60. **SQ** BL	80. **CIR** BL

**Scores Block 1**		**Scores Block 2**		**Scores Block 3**	**Total scores**
Time (s):		Time (s):		Time (s):	Time cost [bocks 3-(1+2)]:
Correct no.: (20)		Correct no.: (20)		Correct no.: (40)	No. cost [blocks 3-(1+2)]:
					No. correct switches: (40)

Notes:

COLOR SHAPE – ANSWER SHEET WITH EXAMPLES OF CORRECTIONS

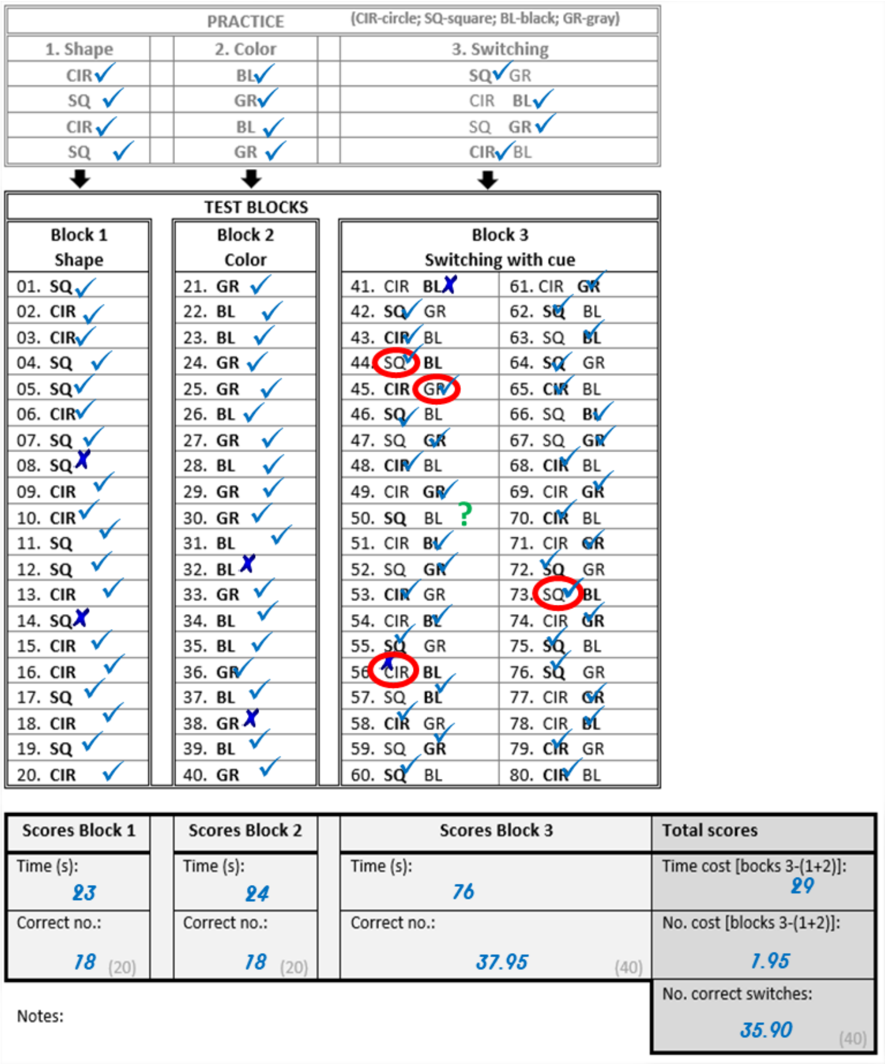

PRACTICE (CIR-circle; SQ-square; BL-black; GR-gray)

1. Shape	2. Color	3. Switching
CIR	BL	SQ GR
SQ	GR	CIR BL
CIR	BL	SQ GR
SQ	GR	CIR BL

TEST BLOCKS

Block 1 Shape	Block 2 Color	Block 3 Switching with cue	
01. SQ	21. GR	41. CIR BL	61. CIR GR
02. CIR	22. BL	42. SQ GR	62. SQ BL
03. CIR	23. BL	43. CIR BL	63. SQ BL
04. SQ	24. GR	44. SQ BL	64. SQ GR
05. SQ	25. GR	45. CIR GR	65. CIR BL
06. CIR	26. BL	46. SQ BL	66. SQ BL
07. SQ	27. GR	47. SQ GR	67. SQ GR
08. SQ	28. BL	48. CIR BL	68. CIR BL
09. CIR	29. GR	49. CIR GR	69. CIR GR
10. CIR	30. GR	50. SQ BL ?	70. CIR BL
11. SQ	31. BL	51. CIR BL	71. CIR GR
12. SQ	32. BL	52. SQ GR	72. SQ GR
13. CIR	33. GR	53. CIR GR	73. SQ BL
14. SQ	34. BL	54. CIR BL	74. CIR GR
15. CIR	35. BL	55. SQ GR	75. SQ BL
16. CIR	36. GR	56. CIR BL	76. SQ GR
17. SQ	37. BL	57. SQ BL	77. CIR GR
18. CIR	38. GR	58. CIR GR	78. CIR BL
19. SQ	39. BL	59. SQ GR	79. CIR GR
20. CIR	40. GR	60. SQ BL	80. CIR BL

Scores Block 1	Scores Block 2	Scores Block 3	Total scores
Time (s): 23	Time (s): 24	Time (s): 76	Time cost [bocks 3-(1+2)]: 29
Correct no.: 18 (20)	Correct no.: 18 (20)	Correct no.: 37.95 (40)	No. cost [blocks 3-(1+2)]: 1.95
Notes:			No. correct switches: 35.90 (40)

Details:

- ✓ Correct classification.
- ✗ Classification error (e.g., says "circle" when the monochromatic rainbow appears) or skipped item.
- ○ Shifting error (made same category classification more than once in a row).
- ? Missing data due to ambiguous responses, or failure to write down answer. If these cases do not exceed 10% of the stimuli in each block, apply the rule of three* to calculate correct no. If missing data exceeds 10%, the examiner must decide how to proceed.

* Rule of three (example for this case):

37 (correct) – 39 (total no. items responded to)

x (correct) – 40 (total no. items)

x = 37.95 (use two digits after the decimal point, rounding decimals to the nearest hundredths)

CATEGORY SWITCHING TASK- TEST ADMINISTRATION AND SCORING

GENERAL INSTRUCTIONS

Which executive domain is assessed by this task: This task assesses the ability to shift (or switch) between two tasks; in this particular case, switching the classification of images (stimuli) according to two categories: as living/non-living entities ("alive" or "dead") and by size while keeping the order of classifications in mind (no clues available).

What the testees are required to do: The testees are required to classify picture stimuli aloud as being either living or non-living ("dead") entities, classify them by size and to sequentially alternate between these category classifications. The tasks must be done as quickly as possible avoiding mistakes. Self-corrections are allowed as long as they occur before the answer to the next stimulus.

What the task entails: This task includes three blocks and is preceded by practice trials. Block 1 requires classification of stimuli as living and non-living ("dead") entities. Block 2 involves classifying stimuli as bigger or smaller than a real soccer ball. In block 3 testees are asked to sequentially alternate between classifications in both these types of category, beginning with "alive or dead". They must keep the order in mind, as there are no external clues regarding which categorization to perform for each stimulus.

Answers are always vocal. The task is self-paced and the testees passes from instructions to the tasks itself by swiping on the screen, pressing a button on the mouse or a key in the computer, or by turning pages, depending on the apparatus or mode of application used in a particular experiment. Testees are, however, not allowed to return to screens/pages that have been already seen. The objective is to complete each block as quickly as possible with the highest possible accuracy.

Which testee characteristics preclude the use of this task to assess executive functions: This task may not assess executive functioning in test takers who are visually impaired or whose vision is not corrected, who have known/diagnosed language or speech disorders or such like that the examiner deems can interfere with performance, who are not familiar with the stimuli and/or do not know the size of what they represent in real life, nor in those who do not understand what it means to be a living or non-living entity or do not know the size of a real soccer ball.

What the examiner does during the task:

1. Recording speed: The examiner must mark how long the testee takes to complete each block in seconds, from the appearance of the first stimulus until the answer to the last stimulus. There is a page that precedes the beginning of each block that serves to help establish when the task will begin. As soon as the testee classifies the last stimulus, the stopwatch must be stopped. First, the answer to the last stimulus must be written down. Only then the examiner must look at the stopwatch and mark the time in seconds in the light gray cells on the answer sheet that correspond to each block.

2. Recording accuracy: To help the examiner keep track, all screens are numbered and the screen number is displayed on the answer sheet at the far left of the cells, followed by the name of the stimulus and their possible category and classification of stimuli: "D" for dead, "A" for live, "B" for bigger, and "S" for smaller. In block 3, however, next to the name of the portrayed objects, there are two sets of letters (for example, "AS" for a being that is alive which is smaller than a soccer ball, such as a picture of a spider).

On the answer sheet, the examiner should work from top left down as the task progresses. They should use tick marks (✓) to indicate the correct answers, **"X"** to indicate errors or skipped stimuli, and **"?"** for ambiguous answers, or in cases in which the examiner fails to take note of the answers. These markings should be made on or above the letters D, A, B and S in all blocks. If testees self-correct answers, the examiner must mark the last given answer for each stimulus made prior to the response to the next stimulus. Self-corrections that occur after should not be considered.

In block 3, while taking down testees' answers, the examiners must disregard bold markings on the letter that indicate the classification under both categories of each stimulus. They must only pay attention to the classification. Thus, if the examiner classifies a spider (AS) as alive, a tick mark (correct classification mark)

must be placed over the letter A. If the answer is “small”, the tick mark must be written about S. If the testee says “dead”, an X should be placed over the A and if the answer is “big”, the X goes on the S. For this example, A is marked in bold because it is the correct classification for that trial <u>if the testee makes no shifting errors</u>. However, the ability to shift categorizations sequentially is only considered in another scoring metric (correct number of shifts and correct classifications when the testee shifts category in that particular trial; see below).

What is crucial for adequate test application:

• The task must not be administered before the examiner practices taking down answers following all the instructions in this manual until this becomes fairly automatic.

• The examiner must make sure that testees can see the stimuli, know the real size of what they represent, can classify them as living and non-living entities and be attentive to any possible naming and perceptual difficulties.

TEST ADMINISTRATION DETAILS

• The examiner must sit next to the testee so he/she can also see the screens or pages.

• The examiner must ask the testee for permission to audio record the session because it is difficult to keep track of answers. This allows the examiner to hear the answers again if they have difficulty marking responses on the answer sheet (presented next).

• The examiner should ask the testee to read the instructions or, if the testees prefer, instructions can be read to them. The examiner must make sure the testee understood the instructions during the practice trials. When in doubt, the practice trials must be repeated until the testee has understood the instructions. Prepare testees to start classifying stimuli as quickly as possible before they pass to the screen that includes the first stimulus.

• Answers that the examiner regards as similar to those required should be counted as valid (e.g. tiny or little are equivalent to “smaller”; larger to bigger; live or living to alive and non-living or not alive to “dead”). In other words, it must not be insisted that testees’ use a specific term to refer to one type of stimuli as long as the given answer is understandable.

• The examiner should start the stopwatch with the non-dominant hand as soon as the first stimulus is visible and turn it off immediately after the last answer was given in each block. Before looking at the stopwatch the examiner must write down the answer to the last stimulus. Only then they should look at the stopwatch and write down the time it took the testee to complete each block (in seconds) on the answer sheet.

• Testees must be allowed to rest between blocks. The examiner should use common sense to determine how long the testee can rest between blocks.

• The testee must complete all blocks in full. There are no interruption criteria for this task.

HOW TO TIME HOW LONG IT TAKES TESTEES TO COMPLETE EACH BLOCK

• The examiner must mark how long the testee takes to complete each block in seconds, from the appearance of the first stimulus until the answer to the last stimulus. There is a page that precedes the beginning of each block that serves to help establish when the task will begin. As soon as the testee classifies the last stimulus, the stopwatch must be stopped. First, the answer to the last stimulus must be written down. Only then should the examiner look at the stopwatch and mark the time in seconds in the answer sheet in the cell that correspond to each condition.

HOW TO MARK SCORES

<u>-Block 1 and 2:</u>

1. For blocks 1 and 2, upon completion of the task, the examiner should count the tick marks (✓ : correct answers) and write the total number on the answer sheet: in the corresponding light gray cells below each block.

Each correct answer equals one point. Do not consider practice trials. The number [e.g. "(20)"] in light gray in these cells shows the maximum possible number of correct answers.

- Block 3:

1. For block 3, upon completion of the task, the examiner must first count the tick marks (✓ : correct answers) regardless of the bold lettering. This will result in the number of correct classifications, which should be noted in the appropriate cell on the answer sheet (light gray cells below block 3). Do not consider practice trials.

- Shifting metrics

1. Calculate the absolute shifting cost measures: the time spent in the shifting block (block 3) minus the sum of the time to complete blocks 1 and 2; write this number in the corresponding dark gray cells at the bottom of the answer sheet. Do the same for correct classifications. Relative shifting cost can also be calculated [e.g. the time spent in the shifting block (block 3) divided by the sum of the time to complete blocks 1 and 2]. An alternative scoring system is Rate Correct Score, or the total number of correct answers per block (or shifting cost of correct answers) divided by the total time taken to complete the block (or shifting cost in time).

2. For block 3 only, another scoring metric should be used that considers whether the testee shifted categorizations sequential, regardless of right or wrong classifications (e.g. said alive for the car stimulus). To facilitate scoring by this criterion, upon completion of the task, shifting errors can be circled on the answer sheet. These errors occur when there are two or more sequential stimuli classifications using the same category (for example, classifying "ring" and "lion" in the alive/dead category in a row). This type of shifting error can be found when the top-to-bottom zigzag pattern is broken, indicated by bold letters. After circling the shifting errors in block 3, count the non-circled responses irrespective if they were correctly classified and add the result to the one but last dark gray cell of the answer sheet. If the testee does not start with "dead or alive", this also counts as a shifting error so the total number of correct shifts is 40 (indicated in light grey in the cell). If there are ambiguous answers or there were items that were skipped, the next classification should be of a different category than the previous answer that was given, maintaining the shifting pattern.

3. The last metric is the number of correct classifications when shifting took place. To count these, highlight the non-circled shifts in block 3 and then count those that received a tick mark (✓). Write down this number in the last dark gray cell on the answer sheet.

Be attentive to any unexpected events

• If there are many sequential errors it is likely that the testee skipped a stimulus and/or that the examiner failed to notice one or more of the testees' answers or self-corrections. The audio recording should be checked.

• Very low scores should only be interpreted as executive difficulties when the examiner believes there are no other perceptual or cognitive deficits which could explain the results (check testee characteristics that preclude the use of this task). In these cases, a referral to the professional that can confirm a possible diagnosis should be provided. If testees are minors, their teacher and/or guardians should be contacted when there are any clinical or cognitive suspicions that can justify testees 'unusual performance. Alternatively, it may be that the testee is unwilling to do the task or to follow instructions. Use common sense to determine whether scores actually reflect the testees' executive abilities.

• Use the space at the bottom of the sheet to record incidents that are considered unusual or unexpected. At times, it is possible only in retrospect to make sense of things that take place during cognitive testing.

MATERIAL: 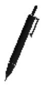 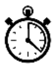 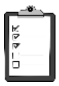 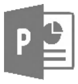 or 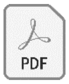

CATEGORY SWITCH – ANSWER SHEET

PRACTICE (A-alive; D-dead; S-small; B-big)								
**1. Alive/Dead**			**2. Big/Small**			**3. Switching**		
Gorilla	**A**		Cup	**S**		Spider	**A**	S
Cup	**D**		Train	**B**		Cup	D	**S**
Train	**D**		Spider	**S**		Gorilla	**A**	B
Spider	**A**		Gorilla	**B**		Train	D	**B**

TEST BLOCKS									
**Block 1 Alive/Dead**		**Block 2 Big/Small**		**Block 3 Switching (begin with alive/dead)**					
01.Ant	**A**	21.Car	**B**	41.Ant	**A**	S	61.Horse	**A**	B
02.Car	**D**	22.Frog	**S**	42.Fork	D	**S**	62.House	D	**B**
03.Horse	**A**	23.Bed	**B**	43.Car	**D**	B	63.Ladybug	**A**	S
04.Giraffe	**A**	24.Ladybug	**S**	44.Whistle	D	**S**	64.Bed	D	**B**
05.House	**D**	25.Ring	**S**	45.Elephant	**A**	B	65.Elephant	**A**	B
06.Whistle	**D**	26.Fork	**S**	46.Key	D	**S**	66.Fork	D	**S**
07.Fork	**D**	27.Giraffe	**B**	47.Ladybug	**A**	S	67.Key	**D**	S
08.Lion	**A**	28.Whistle	**S**	48.Frog	A	**S**	68.Butterfly	A	**S**
09.Butterfly	**A**	29.Lion	**B**	49.Bed	**D**	B	69.Lion	**A**	B
10.Frog	**A**	30.Butterfly	**S**	50.Lion	A	**B**	70.Fork	D	**S**
11.Bed	**D**	31.Elephant	**B**	51.Elephant	**A**	B	71.Car	**D**	B
12.Ladybug	**A**	32.House	**B**	52.House	D	**B**	72.Ring	D	**S**
13.Key	**D**	33.Ant	**S**	53.Horse	**A**	B	73.Ladybug	**A**	S
14.Ring	**D**	34.Key	**S**	54.Butterfly	A	**S**	74.Fridge	D	**B**
15.Fridge	**D**	35.Bed	**B**	55.Giraffe	**A**	B	75.Key	**D**	S
16.Elephant	**A**	36.Horse	**B**	56.Fridge	D	**B**	76.Frog	A	**S**
17.Ant	**A**	37.Butterfly	**S**	57.Frog	**A**	S	77.Giraffe	**A**	B
18.Car	**D**	38.Giraffe	**B**	58.Ant	A	**S**	78.Whistle	D	**S**
19.Whistle	**D**	39.Fridge	**B**	59.Fridge	**D**	B	79.House	**D**	B
20.Lion	**A**	40.Ring	**S**	60.Ring	D	**S**	80.Horse	A	**B**

**Scores Block 1**	**Scores Block 2**	**Scores Block 3**		**Total scores**
Time (s):	Time (s):	Time (s):		Time cost [3-(1+2)]:
Correct classifications (no.): (20)	Correct classifications (no.): (20)	Correct classifications (no.): (40)		No. classification cost [3-(1+2)]: (40)
			Total no. correct classifications in correct switches: (40)	Total no. correct switches (irrespective of classifications): (40)

Notes:

CATEGORY SWITCH – ANSWER SHEET WITH EXAMPLES OF CORRECTIONS

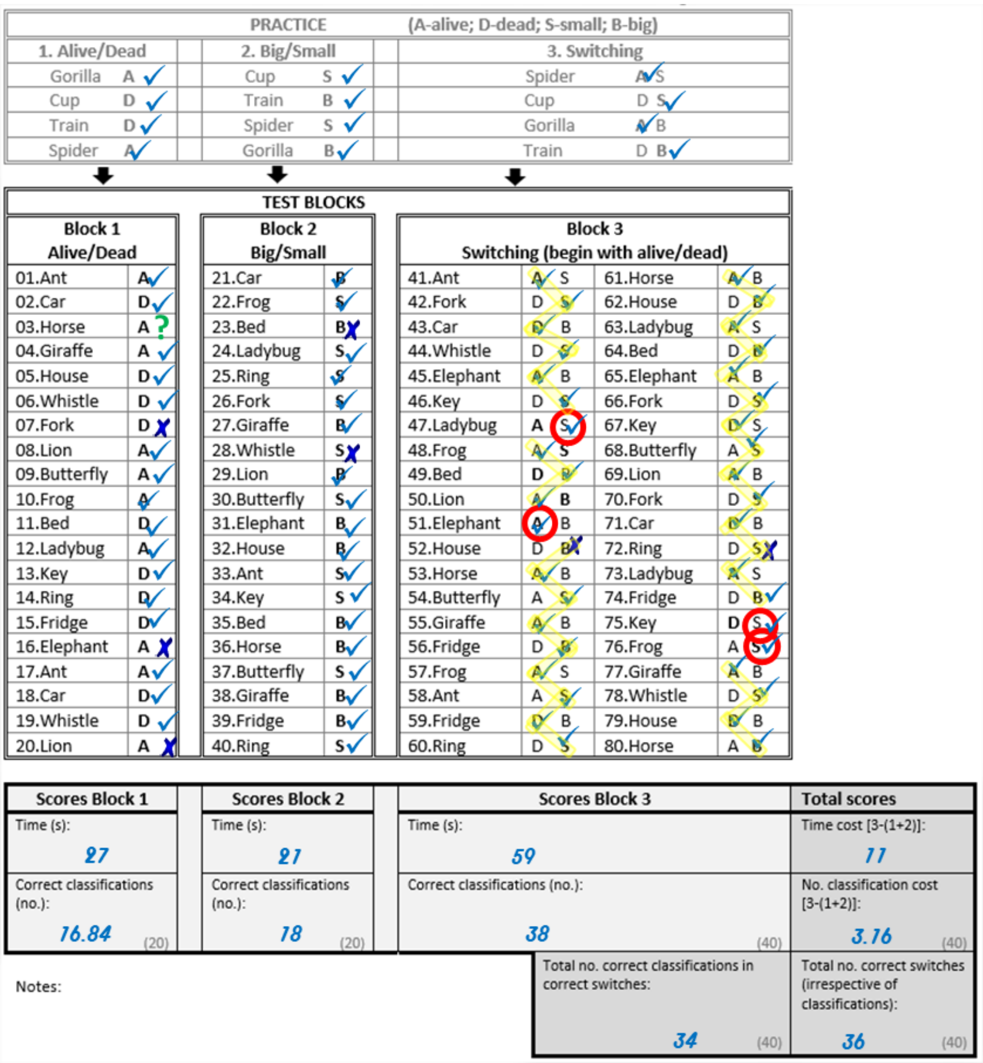

PRACTICE		(A-alive; D-dead; S-small; B-big)
1. Alive/Dead	2. Big/Small	3. Switching
Gorilla A	Cup S	Spider A S
Cup D	Train B	Cup D S
Train D	Spider S	Gorilla A B
Spider A	Gorilla B	Train D B

TEST BLOCKS

Block 1 Alive/Dead		Block 2 Big/Small		Block 3 Switching (begin with alive/dead)			
01.Ant	A	21.Car	B	41.Ant	A S	61.Horse	A B
02.Car	D	22.Frog	S	42.Fork	D S	62.House	D B
03.Horse	A ?	23.Bed	B	43.Car	D B	63.Ladybug	A S
04.Giraffe	A	24.Ladybug	S	44.Whistle	D S	64.Bed	D B
05.House	D	25.Ring	S	45.Elephant	A B	65.Elephant	A B
06.Whistle	D	26.Fork	S	46.Key	D S	66.Fork	D S
07.Fork	D	27.Giraffe	B	47.Ladybug	A S	67.Key	D S
08.Lion	A	28.Whistle	S	48.Frog	A S	68.Butterfly	A S
09.Butterfly	A	29.Lion	B	49.Bed	D B	69.Lion	A B
10.Frog	A	30.Butterfly	S	50.Lion	A B	70.Fork	D S
11.Bed	D	31.Elephant	B	51.Elephant	A B	71.Car	D B
12.Ladybug	A	32.House	B	52.House	D B	72.Ring	D S
13.Key	D	33.Ant	S	53.Horse	A B	73.Ladybug	A S
14.Ring	D	34.Key	S	54.Butterfly	A S	74.Fridge	D B
15.Fridge	D	35.Bed	B	55.Giraffe	A B	75.Key	D S
16.Elephant	A	36.Horse	B	56.Fridge	D B	76.Frog	A S
17.Ant	A	37.Butterfly	S	57.Frog	A S	77.Giraffe	A B
18.Car	D	38.Giraffe	B	58.Ant	A S	78.Whistle	D S
19.Whistle	D	39.Fridge	B	59.Fridge	D B	79.House	D B
20.Lion	A	40.Ring	S	60.Ring	D S	80.Horse	A B

Scores Block 1	Scores Block 2	Scores Block 3		Total scores
Time (s): 27	Time (s): 21	Time (s): 59		Time cost [3-(1+2)]: 11
Correct classifications (no.): 16.84 (20)	Correct classifications (no.): 18 (20)	Correct classifications (no.): 38 (40)		No. classification cost [3-(1+2)]: 3.16 (40)
Notes:			Total no. correct classifications in correct switches: 34 (40)	Total no. correct switches (irrespective of classifications): 36 (40)

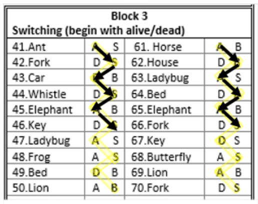

Block 3 Switching (begin with alive/dead)			
41.Ant	A S	61. Horse	A B
42.Fork	D S	62.House	D B
43.Car	D B	63.Ladybug	A S
44.Whistle	D S	64.Bed	D B
45.Elephant	A B	65.Elephant	A B
46.Key	D S	66.Fork	D S
47.Ladybug	A S	67.Key	D S
48.Frog	A S	68.Butterfly	A S
49.Bed	D B	69.Lion	A B
50.Lion	A B	70.Fork	D S

Details:

- ✓ Correct classification.
- ✗ Classification error (e.g. saying "alive" for the drawing that represents the refrigerator).
- ○ Shifting error (following the same type of categorization twice in a row).
- ? Missing data due to ambiguous responses, failure to write response, incomprehensible notes, etc. We suggest that, if these cases do not exceed 10% of the stimuli in each block, the rule of three* be used to estimate the total number of correct responses. When loss of data exceeds 10%, examiners must decide whether to use responses or not.

* Rule of three (example for this case):
16 (correct answers) - 19 (total number of items answered)
x (hits) - 20 (total number of items)
x = 16.84 (use two digits after the decimal point, rounding decimals to the nearest hundredths)

MEMORY NUMBER TASK - INSTRUCTIONS FOR APPLICATION AND CORRECTION

GENERAL INSTRUCTIONS

Which executive domain is assessed by this task: This task assesses the ability to continuously update information in working memory; in this particular case, by keeping sequences of digits in mind.

What the testees are required to do: The testee sees a sequence of single digits and must strive to continuously recall the last three digits that were shown on screen, updating this information as each new digit is presented. The tasks must be done as quickly as possible avoiding mistakes. Self-corrections are allowed as long as they occur before the answer to the next stimulus.

What the task entails: This task includes two test blocks with three trials each and is preceded by practice trials. The testee sees single digits on each screen. From the third screen on, the testee must say aloud the last three numbers (trio) that were presented in the same sequential order as presented, including the number they see on screen, having to update this trio sequentially until the list (trial) of digits ends. When the trials end there appears "???" on screen. The testee must them repeat the last three numbers that were said before, respecting serial order. Remembering these numbers indicates the memory for trios, with no updating requirement, which can be useful to separate retention in working memory from updating this information.

Answers are always vocal. The task is self-paced and the testees passes from instructions to the tasks itself by swiping on the screen, pressing a button on the mouse or a key in the computer, or by turning pages, depending on the apparatus or mode of application used in a particular experiment. Testees are, however, not allowed to return to screens/pages that have been already seen. The objective is to complete each trial as quickly as possible with the highest possible accuracy.

Which testee characteristics preclude the use of this task to assess executive functions: This task may not assess executive functioning in test takers who are visually impaired or whose vision is not corrected, who are not very familiar with Hindu-Arabic numerals, nor in those who have known/diagnosed language or speech disorders, serial order or visuospatial organization and perception difficulties that the examiner deems can interfere with performance.

What the examiner does during the task:

1. Recording speed: The examiner must mark how long the testee takes to complete each trial in seconds, from the appearance of the first stimulus until the answer to the last trio (just before the screen with "???"). The time taken to repeat the last trio need not be timed. Prior to the beginning of each trial there appears a page that prepares the examiner to start the stopwatch. As soon as the testee updates the last trio, the stopwatch must be stopped. First, write down the answer to the last stimulus and the digits that were repeated to check for memory following the "???". Only then the examiner must look at the stopwatch and mark the time in seconds in the light gray cells on the answer sheet that correspond to each trial.

2. Recording accuracy: To help the examiner keep track, all trios are present on the answer sheet. Each updating is indicated as a group of 3 digits (trio) separated by a space from the next trio. The first light gray numbers at the beginning of each sequence on the answer sheet should be said aloud by the examiner as well, but these are not considered as updating opportunities.

On the answer sheet, the examiner should use tick marks (✓) over the trio that was updated correctly (all three numbers must be said in the same order as indicated on the answer sheet). Write **"B"** (blank answer) above any digit that are skipped when the testee indicated that they do not remember that particular digit (consider any wording used that makes this clear). When the subject says less than three numbers (for example: "1 2") and does not indicate in which position he / she has forgotten the remaining number of that trio, consider these as the last two numbers of the trio and mark **"B"** over the first one. When the testee says a wrong digit, write **"X"** over the digit they should have been said and then write the wrong digit above the **"X"**. (This wrong digit will then be considered in the next updating opportunity; see below). Use **"?"**" over digits in the trio to indicate ambiguous answers or in cases in which the examiner failed to understand the answer. If testees

self-correct answers, the examiner must mark the last given answer for each trio made prior to the response to the next trio. Self-corrections that occur after should not be considered.

For the screen/pages including the sign “???”, write down the answer (repetition of the last trio) in the same serial order that was produced by the testee on the three spaces on the answer sheet (“_ _ _”).

What is crucial for adequate test application:

• The task must not be administered before the examiner practices taking down answers following all the instructions in this manual until this becomes fairly automatic.

• The examiner must make sure that testees can see the stimuli, are familiar with Hindu-Arabic numerals and be attentive to any possible naming, serial order or visuospatial organization and perception difficulties.

TEST ADMINISTRATION DETAILS

• The examiner must sit next to the testee so he/she can also see the screens or pages.

• The examiner must ask the testee for permission to audio record the session because it is difficult to keep track of answers. This allows the examiner to hear the answers again if they have difficulty marking responses on the answer sheet (presented next).

• The examiner should ask the testee to read the instructions or, if the testees prefer, instructions can be read to them. The examiner must make sure the testee understood the instructions during the practice trials. When in doubt, the practice trials must be repeated until the testee has understood the instructions. Prepare testees to start naming numbers as quickly as possible before they pass to the screen that includes the first stimulus.

• The examiner should start the stopwatch with the non-dominant hand as soon as the first stimulus is visible and turn it off immediately after the last answer was given in each trial. Before looking at the stopwatch the examiner must write down the answer to the last stimulus. Only then they should look at the stopwatch and write down the time it took the testee to complete each block (in seconds) on the answer sheet.

• Testees must be allowed to rest between blocks. The examiner should use common sense to determine how long the testee can rest between blocks.

• The testee must complete all blocks in full. There are no interruption criteria for this task.

HOW TO SCORE HOW LONG IT TAKES TESTEES TO COMPLETE EACH TRIAL

• The examiner must mark how long the testee takes to complete each trial in seconds, from the appearance of the first stimulus until the answer to the last trio to be updated, when the stopwatch must be stopped. There is a page that precedes the beginning of each block that serves to help establish when the task will begin. The testee will then repeat the last trio and they must be written down in the spaces _ _ _ in the answer sheet. Only then the examiner must look at the stopwatch and mark the time in seconds in the light gray cells on the answer sheet that correspond to each block.

HOW TO MARK SCORES

1. After the task ends, the examiner must count the number of tick marks that indicate correctly updated trios respecting serial order within each trio. Each correctly updated trio corresponds to one point. The total number of correctly updated trios in each trial must be written down in the light gray column to the right of each list of trios. This must be done considering the following (items a to d):

a. Vocally repeating the digits printed in light gray at the beginning of each trial are not counted as updates (updates are considered only from the 4th digit in each list). Likewise, do not consider performance in the practice trials.

b. Inversion of the serial positions of numbers within a trio or saying only one or two of the three digits of a trio correctly, even if in the right serial positions, do not count as a correct updating. In these cases the testee gets zero points for that particular trio.

c. If the testee makes a mistake and includes a digit that is not in the trio, the next trio is considered correct if the updating reflects the prior error. For example, if the trio was 123 and the examiner says 132, this counts as an updating error. However, if 4 is the next number to appear and the testee updates the trio as 324, this is a correct response (correct updating) because the testee made the updating based on what was maintained in their working memory, even though a mistake was made in the preceding trio. Consider the same for blank responses. This is why digits that are skipped or indicated by testees as “blank” (B), and the incorrectly recalled digit, must be written above the correct number in each trio on the answer sheet.

d. The light gray numbers [for example “(2)”] in the lower right corner of the cells correspond to the maximum number of updates (or possible digits to be repeated from the last trio) in that cell.

2. In the last column to the right, write down the total number of digits in the last trio in each trial that was retrieved from memory (maximum of 3), respecting serial order. For example, if the trio was 123 and the testee says 321, their score is 1 (only the digit 2 was in the correct serial position).

3. Add time taken and accuracy (correctly updated and recalled trios) in all test trials (do not consider practice trials) and write the results in the corresponding dark gray cells at the bottom of the answer sheet.

Be attentive to any unexpected events

• If there are many sequential errors it is likely that the testee skipped a stimulus and/or that the examiner failed to notice one or more of the testees’ answers or self-corrections. The audio recording should be checked.

• Very low scores should only be interpreted as executive difficulties when the examiner believes there are no other perceptual or cognitive deficits which could explain the results (check testee characteristics that preclude the use of this task). In these cases, a referral to the professional that can confirm a possible diagnosis should be provided. If testees are minors, their teacher and/or guardians should be contacted when there are any clinical or cognitive suspicions that can justify testees ’unusual performance. Alternatively, it may be that the testee is unwilling to do the task or to follow instructions. Use common sense to determine whether scores actually reflect the testees’ executive abilities.

• Use the space at the bottom of the sheet to record incidents that are considered unusual or unexpected. At times, it is possible only in retrospect to make sense of things that take place during cognitive testing.

MATERIAL: 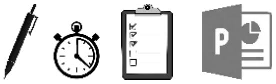 or 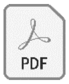

NUMBER MEMORY – ANSWER SHEET

LIST (each trio corresponds to one updating)<br><br>PRACTICE TRIALS	Time(s)	No. correct updatings (max. 2,4 or 6)	No. correct in last 3 nos. (max.3)
**Practice trial 1**			
1 12 123 234 345 456 **567** __ __ __		(4)	
5 52 526 264 641 417 178 789 **893** __ __ __		(6)	
**Practice trial 2**			
3 31 312 124 248 487 **876** __ __ __		(4)	
8 89 893 935 **354** __ __ __		(2)	
3 36 367 674 742 429 291 915 **158** __ __ __		(6)	

TEST BLOCKS

**Test block 1**			
6 65 653 531 **315** __ __ __		(2)	
7 76 762 625 259 598 985 857 **574** __ __ __		(6)	
3 31 314 143 438 386 **869** __ __ __		(4)	

**Test block 2**			
2 27 275 752 524 247 **473** __ __ __		(4)	
5 52 526 264 641 417 173 736 **361** __ __ __		(6)	
8 81 817 173 **736** __ __ __		(2)	

**Total**			
		(24)	(18)

Notes:

NUMBER MEMORY – ANSWER SHEET WITH EXAMPLES OF CORRECTIONS

LIST (each trio corresponds to one updating)	Time(s)	No. correct updatings (max. 2,4 or 6)	No. correct in last 3 nos. (max.3)
**PRACTICE TRIALS**			
**Practice trial 1**			
1 12 123 234 345 456 **567** 5 6 7	16	4 (4)	3
5 52 526 264 641 417 178 789 **893** 8 9 3	40	6 (6)	3
**Practice trial 2**			
3 31 312 124 248 487 **876** 8 7 6 (7)	25	3 (4)	3
8 89 893 935 **354** 9 2 7	16	2 (2)	0
3 36 367 674 742 429 291 915 **158** B 5 8	44	6 (6)	2
**TEST BLOCKS**			
**Test block 1**			
6 65 653 531 **315** 3 1 5	18	2 (2)	3
7 76 762 625 259 598 985 857 **574** 2 8 4 (28)	38	5 (6)	3
3 31 314 143 438 386 **869** 8 6 9 (?)	24	3.64 (4)	3
**Test block 2**			
2 27 275 752 524 247 **473** 4 5 3 (8 8)	24	3 (4)	2
5 52 526 264 641 417 173 736 **361** 3 6 1	38	6 (6)	3
8 81 817 173 **736** 7 3 6 (BB 9)	16	0 (2)	3
Total	158	19.64 (24)	17 (18)

Notes:

Details:

- ✓ Correct updating of an entire trio (light gray numbers at the beginning of each list get no points).
- ✗ Update error (for example, saying a number that is not part of the trio) or ignoring one or more numbers, see examples in the following table.
- *B* (Blank) when testees claims that they do not remember a specific digit in a trio (or report less than three digits for one trio). When the testee reports two of the three digits for a trio and does not indicate where the missing number is in the sequence, the B mark must be placed in the first digit of the trio. Count as updating error.

- **?** Missing data due to ambiguous responses, or failure to write response or incomprehensible notes. We suggest that, if these cases do not exceed 10% of the stimuli in each block, the rule of three* be used to estimate the total number of correct responses. First* use a rule of three considering the total of 12 possible updatings in each block. Then use another rule of three ** to determine the proportion of correct updatings in the particular trial in which **"?"** answers occurred. If the lost data exceeds 10%, the examiner must decide how to proceed.

* Rule of three (example considering block 1, with one **"?"** answer):
10 (hits) - 11 (total number of updates excluding lost data)
x (hits) - 12 (total number of updates)
x = 10.91 (use two digits after the decimal point, rounding decimals to the nearest hundredths)

** Then calculate the equivalence in the trial where there was a **"?"** answer:
10.91 (correct) - 12 (total number)
x (correct) - 4 (updates on trials with **?**)
x = 3.64 (use two digits after the decimal point, rounding decimals to the nearest hundredths)

Examples of how to scores errors

- The testee does not get the trio right (e.g., instead of saying "124" he/she says "724"). Important: the trio that was said must be considered in the next updating attempt. In other words, the testee must update the next trio considering the last 3 numbers he/she remembered last.

- The testee could not remember the last three numbers (instead of saying "354" he/she said "927"). Consider this a total failure of memory, or zero points in the last column.

- The testee misremembers the first and second number from the last trio, so does not gain a point from this updating. Next, the testee must repeat the last trio from memory, so for this repetition what counts is the trio he/she said previously (284) and not the original trio (574). Because in the repetition of the last three numbers the testee says 284, repeats the whole trio correctly, he/ she gets 3 point for "memory".

- The testee mistakens one digit (e.g., instead of saying "524" he/she says "584"), but the following trio is updated correctly, maintaining the number said previously, even if mistakenly: consider an error for the first trio and correct answer for the second.

- The testee does not remember the first two digits (1 and 7) of the first trio and indicates this by saying something in the line of "blank". They also misremembers the first number of the next trio (instead of "7" he/she says "9"). Consider these two updating errors.

TWO-BACK TASK– TEST ADMINISTRATION AND SCORING

GENERAL INSTRUCTIONS

Which executive domain is assessed by this task: This task assesses the ability to update content in working memory; in this particular case, by keeping track of the spatial location of stimuli.

What the testees are required to do: In this task, there are test blocks preceded by practice trials. The testee must keep track of the spatial location of black squares that change position from screen to screen and answer whether the location on screen is the same or different as that of the black square two screens back. The tasks must be done as quickly as possible avoiding mistakes. Self-corrections are allowed as long as they occur before the answer to the next stimulus.

What the task entails: This task includes three blocks and is preceded by various practice trials. Testees must say aloud whether the black square they see on screen is in the same position (verbal answer that is equivalent to "=") or in a different position (verbal answer that is equivalent to "≠") than the black square that appeared two pages back. Note that no response is required on the first two pages / screens in all blocks (NRN = no response is required).

Answers are always vocal. The task is self-paced and the testees passes from instructions to the tasks itself by swiping on the screen, pressing a button on the mouse or a key in the computer, or by turning pages, depending on the apparatus or mode of application used in a particular experiment. Testees are, however, not allowed to return to screens/pages that have been already seen. The objective is to complete each trial as quickly as possible with the highest possible accuracy.

Which testee characteristics preclude the use of this task to assess executive functions: This task may not assess executive functioning in test takers who are visually impaired or whose vision is not corrected, nor in those who have known/diagnoses language or speech disorders, serial order or visuospatial organization and perception difficulties that the examiner deems can interfere with performance.

What the examiner does during the task:

1. Recording speed: The examiner must mark how long the testee takes to complete each block in seconds, from the appearance of the first stimulus until the answer to the last stimulus. There is a page that precedes the beginning of each block that serves to help establish when the task will begin. As soon as the testee answers "same" or "different" to the last stimulus, the stopwatch must be stopped. First, the answer to the last stimulus must be written down. Only then the examiner must look at the stopwatch and mark the time in seconds in the light gray cells on the answer sheet that correspond to each block.

2. Recording accuracy: The screens are numbered to help the examiner keep track of answers (these numbers appear in the lower right corner of the pages). On the answer sheet the examiner should use tick marks (✓) to indicate the correct answers, **"X"** to indicate errors or skipped stimuli, and **"?"** for ambiguous answers, or in cases in which the examiner fails to take note of the answers. **"B"** (blank answer indicative; consider any wording used that makes this clear) should be used when the testee claims to have forgotten the location of two squares back (count as an error). These markings should be made on or above the = or ≠ symbols that indicate the possible answers on the answer sheet. If testees self-correct answers, the examiner must mark the last given answer for each stimulus made prior to the response to the next stimulus. Self-corrections that occur after should not be considered.

What is crucial for adequate test application:

• The task must not be administered before the examiner practices taking down answers following all the instructions in this manual until this becomes fairly automatic.

• The examiner must make sure that testees can see the stimuli, and be attentive to any possible naming, serial order or visuospatial organization and perception difficulties.

TEST ADMINISTRATION DETAILS

• The examiner must sit next to the testee so he/she can also see the screens or pages.

• The examiner must ask the testee for permission to audio record the session because it is difficult to keep track of answers. This allows the examiner to hear the answers again if they have difficulty marking responses on the answer sheet (presented next).

• The examiner should ask the testee to read the instructions or, if the testees prefer, instructions can be read to them. The examiner must make sure the testee understood the instructions during the practice trials. When in doubt, the practice trials must be repeated until the testee has understood the instructions. Prepare testees to pay attention to the spatial location of the target square before they pass to the first test screen.

• The examiner should start the stopwatch with the non-dominant hand as soon as the first stimulus is visible and turn it off immediately after the last answer was given in each trial. Before looking at the stopwatch the examiner must write down the answer to the last stimulus. Only then they should look at the stopwatch and write down the time it took the testee to complete each trial (in seconds) on the answer sheet.

• Testees must be allowed to rest between blocks. The examiner should use common sense to determine how long the testee can rest between blocks.

• The testee must complete all blocks in full. There are no interruption criteria for this task.

HOW TO TIME HOW LONG IT TAKES TESTEES TO COMPLETE EACH BLOCK

• The examiner must mark how long the testee takes to complete each block in seconds, from the appearance of the first stimulus until the answer to the last stimulus. There is a page that precedes the beginning of each block that serves to help establish when the task will begin. As soon as the testee classifies the last stimulus, the stopwatch must be stopped. First, the answer to the last stimulus must be written down. Only then should the examiner look at the stopwatch and mark the time in seconds in the answer sheet in the cell that correspond to each condition.

HOW TO MARK SCORES

1. Upon completion of the task, the examiner must write down the total number of correct answers in the cells to the right of each block, remembering that each correct answer equals one point. The light gray number [e.g. “(22)”] in the cells indicate the maximum possible number of correct answers that can be placed in that cell.

2. The experimenter must add the time and correct answers in all three blocks (exclude training scores) and write the result in the appropriate cell in dark gray at the bottom of the answer sheet.

Be attentive to any unexpected events

• If there are many sequential errors it is likely that the testee skipped a stimulus and/or that the examiner failed to notice one or more of the testees’ answers or self-corrections. The audio recording should be checked.

• Very low scores should only be interpreted as executive difficulties when the examiner believes there are no other perceptual or cognitive deficits which could explain the results (check testee characteristics that preclude the use of this task). In these cases, a referral to the professional that can confirm a possible diagnosis should be provided. If testees are minors, their teacher and/or guardians should be contacted when there are any clinical or cognitive suspicions that can justify testees ’unusual performance. Alternatively, it may be that the testee is unwilling to do the task or to follow instructions. Use common sense to determine whether scores actually reflect the testees’ executive abilities.

• Use the space at the bottom of the sheet to record incidents that are considered unusual or unexpected. At times, it is possible only in retrospect to make sense of things that take place during cognitive testing.

MATERIAL: 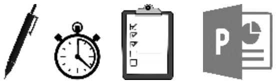 or 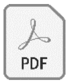

TWO-BACK – ANSWER SHEET

PRACTICE

Practice trial 1												Time (s)	Correct (no.)
1) NRN	2) NRN	3) =	4) ≠	5) ≠	6) ≠	7) =	8) ≠	9) ≠	10) ≠	11) ≠	12) =		
**Practice trial 2**													
13) NRN	14) NRN	15) ≠	16) =	17) ≠	18) ≠	19) ≠	20) =	21) ≠	22) ≠	23) =	24) ≠		
25) ≠	26) ≠	27) =	28) ≠	29) ≠	30) =	31) ≠	32) ≠	33) ≠	34) =	35) ≠	36) ≠		(22)

TEST BLOCKS

Block 1													
1) NRN	2) NRN	3) ≠	4) ≠	5) =	6) ≠	7) ≠	8) =	9) ≠	10) =	11) ≠	12) ≠		
13) ≠	14) =	15) ≠	16) ≠	17) ≠	18) ≠	19) =	20) ≠	21) ≠	22) ≠	23) =	24) ≠		(22)

Block 2													
25) NRN	26) NRN	27) =	28) =	29) ≠	30) ≠	31) ≠	32) ≠	33) ≠	34) ≠	35) =	36) ≠		
37) ≠	38) ≠	39) =	40) ≠	41) ≠	42) ≠	43) ≠	44) =	45) ≠	46) ≠	47) ≠	48) =		(22)

Block 3													
49) NRN	50) NRN	51) ≠	52) =	53) ≠	54) ≠	55) =	56) ≠	57) ≠	58) ≠	59) ≠	60) ≠		
61) =	62) ≠	63) ≠	64) =	65) ≠	66) ≠	67) ≠	68) =	69) ≠	70) ≠	71) =	72) ≠		(22)

	Time (s)	Correct (no.)
**Total**		(66)

NRN=no response necessary
Notes:

TWO-BACK – ANSWER SHEET WITH EXAMPLES OF CORRECTIONS

PRACTICE

Practice trial 1													
1) NRN	2) NRN	3) = ✓	4) ≠ ✓	5) ≠ ✓	6) ≠ ✓	7) = ✓	8) ≠ ✓	9) ≠ ✓	10) ≠ ✓	11) ≠ ✓	12) = ✓		
**Practice trial 2**												**Time (s)**	**Correct (no.)**
13) NRN	14) NRN	15) ≠ ✓	16) = ✗	17) ≠ ✓	18) ≠ ✓	19) ≠ ✓	20) = ✓	21) ≠ ✓	22) ≠ ✓	23) = ✓	24) ≠ ✓	80	21
25) ≠ ✓	26) ≠ ✓	27) = ✓	28) ≠ ✓	29) ≠ ✓	30) = ✓	31) ≠ ✓	32) ≠ ✓	33) ≠ ✓	34) = ✓	35) ≠ ✓	36) ≠ ✓		(22)

TEST BLOCKS

Block 1													
1) NRN	2) NRN	3) ≠ ✓	4) ≠ ✓	5) = ✓	6) ≠ ✓	7) ≠ ✓	8) = ✗	9) ≠ ✓	10) = ✓	11) ≠ ✓	12) ≠ ✓	71	19
13) ≠ ✓	14) = ✓	15) ≠ ✓	16) ≠ ✗	17) ≠ ✓	18) ≠ ✓	19) = ✓	20) ≠ ✓	21) ≠ ✓	22) ≠ ✓	23) = ✗	24) ≠ ✓		(22)

Block 2													
25) NRN	26) NRN	27) = ✓	28) = ✓	29) ≠ ✓	30) ≠ ✓	31) ≠ B	32) ≠ ✓	33) ≠ ✓	34) ≠ ✓	35) = ✗	36) ≠ ✓	69	19
37) ≠ ✓	38) ≠ ✓	39) = ✗	40) ≠ ✓	41) ≠ ✓	42) ≠ ✓	43) ≠ ✓	44) = ✓	45) ≠ ✓	46) ≠ ✓	47) ≠ ✓	48) = ✓		(22)

Block 3													
49) NRN	50) NRN	51) ≠ ✓	52) = ✗	53) ≠ ✓	54) ≠ ✓	55) = ?	56) ≠ ✓	57) ≠ ✓	58) ≠ ✓	59) ≠ ✓	60) ≠ ✓	68	19.90
61) = B	62) ≠ ✓	63) ≠ ✓	64) = ✓	65) ≠ ✓	66) ≠ ✓	67) ≠ ✓	68) = ✓	69) ≠ ✓	70) ≠ ✓	71) = ✓	72) ≠ ✓		(22)

	Time (s)	Correct (no.)
Total	208	57.90 (66)

NRN=no response necessary
Notes:

Details:

- ✓ Correct answer.
- ✗ Update error (for example, says "same" when correct is "different") or item ignored.
- *B* (Blank) when participant says they do not remember the location of two squares behind. Count as error.
- ? Missing data due to ambiguous responses, failure to write response, or incomprehensible notes. If these cases do not exceed 10% of the stimuli in each block, apply the three * rule to calculate the correct number and consider the time as normal (need not be adjusted). If the lost data exceeds 10%, the examiner must decide how to proceed.

* Rule of three (example for this case):
19 (correct answers) - 21 (total number of answered + blank items)
x (hits) - 22 (total number of items)
x = 19.90 (use two digits after the decimal point, rounding decimals to the nearest hundredth)
