# Supplementary material for: An Adaptable, Open-Access Test Battery to Study the Fractionation of Executive-Functions in Diverse Populations
Source: Front Psychol. 2021 Mar 30;12:627219. doi: 10.3389/fpsyg.2021.627219 (PMC8042159; doi:10.3389/fpsyg.2021.627219)
Supplement: Supplementary file 3 [file Data_Sheet_3.pdf]

frontiers

Supplementary Material III

Administration and correction Manual of the Free Research Executive Evaluation (FREE) test battery (Portuguese version)

Manual de administração e correção da bateria de testes Free Research Executive Evaluation (FREE) (versão em português do Brasil)

Este é um manual de aplicação e correção, com folhas de respostas, de uma bateria de testes de acesso aberto para fins de pesquisa que pode ser usada para avaliar o fracionamento (ou a unidade e diversidade) das funções executivas. O manual descreve como aplicar e corrigir duas tarefas de cada um dos domínios de inibição, alternância e atualização executiva.

Para as bases teóricas subjacentes à seleção dessas tarefas, estímulos e modo de resposta, consulte o documento principal **"Uma bateria de teste adaptável de acesso livre para estudar o fracionamento das Funções Executivas em diversas populações"** ao qual este manual está anexado. As tarefas em si estão em formato pptx e pdf, em Inglês e Português.

Usuários de países ou culturas nas quais o Inglês ou Português do Brasil não são o idioma oficial devem adaptar as instruções e estímulos para seu próprio contexto, seguindo as sugestões na descrição detalhada das tarefas, no documento principal e Material Suplementar I.

Este material está registrado sob uma Licença Creative Commons Atribuições (CC BY-SA), o que significa que qualquer pessoa pode usar e modificar seu conteúdo desde que o presente trabalho seja mencionado. Em mais detalhes:

- Atribuição (by): requer que os usuários das tarefas propostas aqui concedam crédito aos autores do manual original (em outras palavras, cite este trabalho). Isso não significa que apoiamos o uso em outros trabalhos ou publicações.

- Compartilhar (sa): permite que o usuário copie, distribua, exiba, execute e modifique as tarefas originais, modos de correção, etc., desde que os trabalhos sejam distribuídos nos mesmos termos (CC BY-SA). Caso haja modificações, essas devem ser indicadas claramente. Se os usuários quiserem distribuir obras modificadas sob outros termos, eles(as) deverão obter a permissão dos autores do presente trabalho.

Considerações para uso dos testea

Pesquisadores interessados em usar esta bateria de testes devem considerar os seguintes pontos, depois de ler a justificativa para a escolha de tarefas e entender suas características, instruções, procedimentos de administração e correção:

1. As instruções e estímulos devem ser adaptados para cada população estudada, levando em consideração a facilidade com que esses são identificados e, se possível, a extensão (número de sílabas, fonemas) das respostas vocais. Se as normas para nomeação de figuras, por exemplo, não estiverem disponíveis, estudos-piloto devem ser conduzidos para garantir que as populações em estudo as identifiquem facilmente e entendam como devem ser usadas (por exemplo, entendam o que é uma algo vivo versus não-vivo; ser de uma cultura em que “feliz” é automaticamente considerado “oposto” ao triste; conhecer o tamanho real dos estímulos representados). O mesmo deve ser feito com relação à clareza das instruções.

2. As tarefas descritas aqui não foram desenvolvidas para o diagnóstico de dificuldades cognitivas. Se o objetivo é caracterizar a cognição de um grupo clínico, um grupo controle deve ser incluído com características semelhantes à da população alvo sob investigação.

3. Se o desempenho dos participantes for avaliado em dois momentos diferentes, outra versão de cada tarefa pode ser criada alternando a ordem dos ensaios em cada bloco da tarefa e balanceando as versões dos testes entre participantes e grupos. O desempenho em duas tarefas propostas para avaliar um mesmo domínio executivo não deve ser comparado diretamente porque ele variará de acordo com a tarefa, mesmo que ambas avaliem o mesmo constructo. Considere também os efeitos de aprendizado que diminuem a detecção de uso de funcções executivas quando as pessoas praticam tarefas executivas.

4. Se várias das tarefas propostas forem usadas em diferentes participantes, considere variar a ordem de apresentação entre os participantes para minimizar efeitos de fadiga.

5. Propusemos aqui escores na forma de Rate Correct Scores-RCS (Taxa de Respostas Corretas) - o número de respostas corretas dividido pelo tempo necessário para concluir as tarefas/blocos. Esse tipo de métrica tem a vantagem de levar em consideração compensações de precisão em função da velocidade de resposta (*speed-accuracy trade-off*). No entanto, o número de erros ou de respostas corretas e/ou o tempo necessário para concluir tarefas, ensaios ou blocos podem ser utilizados, embora estes escores apresentem desvantagens psicométricas. Propomos, ainda, o uso de escores de custos executivos absolutos (desempenho em blocos executivos *subtraído* do desempenho em blocos de controle ou vice-versa), mas custos relativos (desempenho em blocos executivos *divididos* por desempenho em blocos de controle ou vice-versa) podem ser usados.

6. A bateria de testes foi elaborada para permitir a obtenção de variáveis latentes de cada domínio de funções executivas a partir dos escores das duas tarefas de cada domínio. Todavia, isso não inviabiliza o uso de escores individuais em cada teste.

TAREFA STROOP VICTORIA – ADMINISTRAÇÃO E PONTUAÇÃO

INSTRUÇÕES GERAIS

Qual domínio executivo é avaliado por esta tarefa: Esta tarefa avalia a inibição de respostas preponderantes/automáticas; nesse caso em particular, a capacidade de inibir a tendência automática de ler palavras escritas e, em vez disso, nomear cores.

O que é requerido que os examinandos façam: Nesta tarefa, os examinandos são solicitados a nomear em voz alta a cor da tinta dos estímulos apresentados em uma única página, da esquerda para a direita. As tarefas devem ser realizadas o mais rápido possível, evitando erros. As instruções podem ser lidas pelo examinando ou lidas a ele(a). Os examinandos podem usar seus dedos para seguir cada linha durante a execução da tarefa, pois isso ajuda algumas pessoas a não perderem a ordem durante a nomeação. Autocorreções são permitidas desde que ocorram antes da resposta referente ao próximo estímulo.

O que a tarefa envolve: Essa tarefa inclui três blocos. Nessa tarefa, os examinandos podem ver como são os estímulos alvo durante as instruções, mas não há treinos.Em todos os blocos os examinandos devem nomear as cores que veem (e não ler os estímulos que são palavras nos blocos em que palavras estão presentes). No bloco 1, os estímulos são retângulos coloridos. No bloco 2, os estímulos são palavras (que não são nomes de cores) impressas em tinta colorida. No bloco 3, os estímulos são palavras que são nomes de cores, impressas em cores de tinta incongruentes com as palavras escritas (por exemplo, a palavra "rosa" escrita em tinta azul).

As respostas são sempre vocais. A velocidade de realização da tarefa é determinada pelos próprios examinandos, poisles(as) passam das instruções para a tarefa deslizando o dedo na tela (em casos de telas sensíveis ao toque), pressionando um botão do mouse ou uma tecla no computador ou virando páginas, dependendo do equipamento ou modo de aplicação usado em cada experimento. Os examinandos não devem, no entanto, retornar aos slides/páginas que já foram vistos. O objetivo é concluir cada bloco o mais rápido possível evitando erros.

Quais características do examinando impedem o uso desta tarefa para avaliar as funções executivas: Esta tarefa não deve ser realizada para avaliar o funcionamento executivo de pessoas com deficiência visual ou cuja visão não esteja corrigida, que tenham diagnóstico de distúrbios de linguagem, dificuldade em discriminar cores, não sabem ler com relativa fluência ou outras dificuldades que o aplicador julgar que podem interferir no desempenho.

O que o aplicador faz durante a tarefa:

1. Marcação da velocidade de realização da tarefa: O aplicador deve marcar quanto tempo o examinando leva para concluir cada bloco em segundos, desde o aparecimento do primeiro estímulo até a resposta referente ao último estímulo. Há uma página que precede o início de cada bloco que serve para ajudar a estabelecer quando a tarefa começará. Assim que o examinando nomear a cor do último estímulo, o cronômetro deverá ser parado. A resposta ao último estímulo deve ser anotada em primeiro lugar. Somente então o aplicador deve olhar para o cronômetro e marcar o tempo em segundos nas células cinza claro na folha de respostas que correspondem a cada bloco.

2. Marcação da acurácia das respostas: Na folha de respostas, o aplicador deve usar marcas de tique (✓) para indicar as respostas corretas; **"X"** para indicar erros ou estímulos sem resposta; e **"?"** para respostas ambíguas, ou nos casos em que o aplicador não conseguir anotar as respostas. Essas marcações devem ser feitas sobre o nome de cada cor na folha de respostas. Se o examinando se autocorrigir antes da resposta ao estímulo seguinte, o aplicador deve marcar a última resposta fornecida. As autocorreções que ocorrem depois não devem ser consideradas.

O que é crucial para a aplicação adequada do teste:

• A tarefa não deve ser administrada antes que o aplicador treine bem a marcação das respostas seguindo todas as instruções deste manual, até que isso se torne automático.

• O aplicador deve estar atento a possíveis dificuldades de nomeação e garantir que os examinandos conseguem ver os estímulos e sabem ler com relativa fluência. Para verificar isso, podem pedir que os examinandos leiam parte das instruções. Os examinandos também devem relatar não ter dificuldades com discriminações de cores. Respostas muito vagarosas podem indicar isso.

DETALHES DA ADMINISTRAÇÃO DO TESTE

• O aplicador deve sentar-se ao lado do examinando para que ele(a) também possa ver os estímulos.

• O aplicador deve pedir permissão ao examinando para gravar a sessão em áudio, pois é difícil acompanhar as respostas. Isso permite que o aplicador ouça as respostas novamente caso tenha dificuldade em marcá-las na folha de respostas.

• O aplicador deve pedir ao examinando que leia as instruções ou, se este(a) preferir, as instruções podem ser lidas para ele(a). O aplicador deve garantir que o examinando tenha entendido as instruções antes de iniciar a tarefa. Em caso de dúvida, as instruções devem ser repetidas. O examinando deve estar preparado para começar a nomear cores o mais rápido possível antes de ir para a página que inclui todos os estímulos.

• O aplicador deve acionar o cronômetro com a mão não dominante assim que o primeiro estímulo estiver visível e parar imediatamente após a resposta referente ao último estímulo em cada bloco. Antes de olhar para o cronômetro o aplicador deve anotar a resposta referente ao último estímulo. Só então ele(a)s deve olhar para o cronômetro e anotar o tempo que o examinando levou para concluir cada bloco (em segundos) na folha de respostas.

• Os examinandos podem descansar entre os blocos. O aplicador deve usar o bom senso para determinar por quanto tempo o examinando pode fazê-lo.

• O examinando deve completar todos os blocos na íntegra. Não há critérios de interrupção para esta tarefa.

COMO CRONOMETRAR QUANTO TEMPO O EXAMINANDO LEVA PARA COMPLETAR CADA BLOCO

• O aplicador deve marcar quanto tempo o examinando leva para concluir cada bloco em segundos, desde o aparecimento do primeiro estímulo até a resposta referente ao último estímulo. Há uma página que precede o início de cada bloco que serve para ajudar a estabelecer quando a tarefa começará. Assim que o examinando classificar o último estímulo a cronometragem deve ser parada. A resposta ao último estímulo deve ser anotada em primeiro lugar. Somente então o aplicador deve olhar para o cronômetro e marcar o tempo, em segundos, nas células específicas que correspondem a cada bloco.

COMO MARCAR PONTOS

1. Após a conclusão da tarefa o aplicador deve contar e anotar o número total de respostas corretas nas células correspondentes para cada bloco, em cinza claro à direita da folha de respostas, lembrando que cada resposta correta é equivalente a um ponto. O número cinza claro “(24)” nas células indica o número máximo possível de respostas corretas.

2. O aplicador pode então calcular os custos absolutos da inibição (tempo necessário para concluir o bloco 3 menos o tempo necessário para concluir o bloco 1 e/ou 2). O mesmo deve ser feito para as respostas corretas. Os custos de inibição relativa podem ser calculados usando divisão em vez de subtração (isto é, desempenho no bloco 3 / bloco 1). Os resultados devem ser anotados na última célula na parte inferior da folha de respostas (em cinza escuro). Um sistema de pontuação alternativo é o Rate Correct Score (RCS), ou o número total de respostas

corretas por bloco (ou custo de inibição das respostas corretas) dividido pelo tempo total necessário para concluir o bloco (ou custo de inibição no tempo).

Esteja atento a quaisquer eventos inesperados

• Se houver muitos erros sequenciais é provável que o examinando tenha omitido (“pulado”) um estímulo e/ou que o aplicador não tenha anotado uma ou mais das respostas ou autocorreções dos examinandos. A gravação de áudio deve ser verificada.

• Pontuações muito baixas só devem ser interpretadas como dificuldades executivas quando o aplicador acreditar que não há outros déficits perceptivos ou cognitivos que possam explicar os resultados (verifique as características dos examinandos que impedem o uso desta tarefa). Nesses casos, os examinandos devem ser encaminhados ao tipo de profissional que possa confirmar um possível diagnóstico. Se o examinando for menor de idade o professor e/ou responsáveis devem ser contatados quando houver suspeitas clínicas ou cognitivas que possam justificar o desempenho incomum dos examinandos. Alternativamente, pode ser que o examinando não esteja disposto a executar a tarefa ou seguir as instruções. Use o bom senso para determinar se as pontuações realmente refletem habilidades executivas.

• Use o espaço na parte inferior da folha para registrar incidentes considerados incomuns ou inesperados. Às vezes, apenas em retrospecto é possível entender a importância de eventos que ocorreram durante a avaliação cognitiva.

MATERIAL: 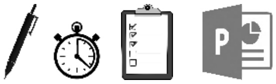 ou 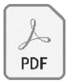

STROOP VICTORIA – FOLHA DE RESPOSTA

Bloco 1: Retângulos				Tempo (s)	Acertos (nº)
verde	rosa	azul	preto		
rosa	preto	verde	azul		
azul	verde	rosa	preto		
preto	rosa	azul	verde		
rosa	azul	verde	preto		
preto	verde	azul	rosa		(24)

Bloco 2: Palavras que não são nomes de cores					
verde	rosa	azul	preto		
rosa	preto	verde	azul		
azul	verde	rosa	preto		
preto	rosa	azul	verde		
rosa	azul	verde	preto		
preto	verde	azul	rosa		(24)

Bloco 3: Palavras que são nomes de cores					
verde	rosa	azul	preto		
rosa	preto	verde	azul		
azul	verde	rosa	preto		
preto	rosa	azul	verde		
rosa	azul	verde	preto		
preto	verde	azul	rosa		(24)

	Tempo (s) (Bloco 3-1)	Acertos nº (Bloco 3-1)
**Escore custo de Inibição**		

Observações:

STROOP VICTORIA – FOLHA DE RESPOSTA COM EXEMPLO DE CORREÇÕES

Bloco 1: Retângulos				Tempo (s)	Acertos (nº)
verde ✓	rosa ✓	azul ✓	preto ✓	24	24
rosa ✓	preto ✓	verde ✓	azul ✓		
azul ✓	verde ✓	rosa ✓	preto ✓		
preto ✓	rosa ✓	azul ✓	verde ✓		
rosa ✓	azul ✓	verde ✓	preto ✓		
preto ✓	verde ✓	azul ✓	rosa ✓		(24)

Bloco 2: Palavras que não são nomes de cores				Tempo (s)	Acertos (nº)
verde ✓	rosa ✓	azul ✓	preto ✓	28	23
rosa ✓	preto ✓	verde ✓	azul ✓		
azul ✓	verde ✓	rosa ✗	preto ✓		
preto ✓	rosa ✓	azul ✓	verde ✓		
rosa ✓	azul ✓	verde ✓	preto ✓		
preto ✓	verde ✓	azul ✓	rosa ✓		(24)

Bloco 3: Palavras que são nomes de cores				Tempo (s)	Acertos (nº)
verde ✓	rosa ✓	azul ✓	preto ✓	32	21.91
rosa ✓	preto ✓	verde ✓	azul ✗		
azul ✗	verde ✓	rosa ✓	preto ✓		
preto ✓	rosa ✓	azul ✓	verde ✓		
rosa ✓	azul ✓	verde ✓	preto ✓		
preto ✓	verde ?	azul ✓	rosa ✓		(24)

	Tempo (s) (Bloco 3-1)	Acertos nº (Bloco 3-1)
Escore custo de Inibição	8	-2.09

Observações:

Detalhes:

- ✓ Respostas corretas.
- ✗ Erro de inibição (por exemplo, dizer “azul" quando deveria dizer "verde") ou quando o examinando “pula” o estímulo.
- ? Dados perdidos devido a respostas ambíguas, falha para escrever a resposta, anotações incompreensíveis, etc. Sugerimos que, se esses casos não excederem 10% dos estímulos em cada bloco, a regra de três* seja usada para estimar o número total de respostas corretas. Quando a perda de dados exceder 10%, os aplicadores devem decidir se vão usar as respostas ou não. Se isso ocorrer no bloco 3 os custos executivos não poderão ser calculados (devem ser considerados valores perdidos).
- O custo aqui está exemplificado como custo absoluto do block 3 em relação ao bloco 1.

* Regra de três (exemplo para esse caso do bloco 3):

21 (respostas corretas) – 23 (nº total itens respondidos - o item marcado com ? não é considerado)

x (acertos) – 24 (nº total itens)

x= 21.91 (use dígitos após o ponto decimal, arredondando os decimais para os centésimos mais próximos)

TAREFA STROOP FELIZ TRISTE – ADMINISTRAÇÃO E PONTUAÇÃO

INSTRUÇÕES GERAIS

Qual domínio executivo é avaliado por esta tarefa: Esta tarefa avalia a capacidade de inibir respostas preponderantes/automáticas; nesse caso em particular, a tendência automática de nomear expressões faciais e, em vez disso, nomear outra emoção ("oposta").

O que é requerido que os examinandos façam: Nesta tarefa, os examinandos são solicitados a nomear as emoções dos estímulos faciais, da esquerda para a direita. As tarefas devem ser realizadas o mais rápido possível, evitando erros. As instruções podem ser lidas pelo examinando ou lidas para ele(a). Os examinandos podem usar seus dedos para seguir cada linha durante a execução da tarefa, pois isso ajuda algumas pessoas a não perderem a ordem durante a nomeação. Autocorreções são permitidas desde que ocorram antes da resposta referente ao próximo estímulo.

O que a tarefa envolve: Esta tarefa inclui três blocos. Nessa tarefa, os examinandos podem ver como são os estímulos alvo durante as instruções, mas não há ensaios para treino. No bloco 1, são apresentados emojis que expressam felicidade e tristeza. No bloco 2, os estímulos são as mesmas expressões faciais, mas em fotografias em preto e branco. Em ambos os casos, os examinandos são solicitados a nomear a emoção de cada estímulo. Os estímulos no bloco 3 são iguais aos do bloco 2, mas, neste caso, os examinandos devem nomear as emoções "opostas" (por exemplo, dizer "triste" quando virem um "rosto feliz").

As respostas são sempre vocais. A velocidade de realização da tarefa é determinada pelos próprios examinandos. Eles(as) passam das instruções para as tarefas, e entre tarefas, deslizando o dedo na tela (em casos de telas sensíveis ao toque), pressionando um botão do mouse ou uma tecla no computador ou virando páginas, dependendo do equipamento ou modo de aplicação usado em cada experimento. Os examinandos não devem, no entanto, retornar aos slides/páginas que já foram vistos. O objetivo é concluir cada bloco o mais rápido possível evitando erros.

Quais características do examinando impede o uso desta tarefa para avaliar as funções executivas:

Esta tarefa não deve ser realizada para avaliar o funcionamento executivo de pessoas com deficiência visual ou cuja visão não esteja corrigida, que tenham diagnóstico de distúrbios de linguagem, dificuldade em identificar emoções (como indivíduos no espectro do autismo) e outras condições que o aplicador julgar poderem interferir no desempenho.

O que o aplicador faz durante a tarefa:

1. Marcação da velocidade de realização da tarefa: O aplicador deve marcar quanto tempo o examinando leva para concluir cada bloco em segundos, desde o aparecimento do primeiro estímulo até a resposta referente ao último estímulo. Há uma página que precede o início de cada bloco que serve para ajudar a estabelecer quando a tarefa começará. Assim que o examinando nomear a emoção (ou emoção oposta) do último estímulo, o cronômetro deverá ser parado. A resposta ao último estímulo deve ser anotada, primeiro. Somente então o aplicador deve olhar para o cronômetro e marcar o tempo, em segundos, nas células cinza claro na folha de respostas que correspondem a cada bloco.

2. Marcação da acurácia das respostas: Na folha de respostas, o aplicador deve usar marcas de tique (✓) para indicar as respostas corretas, **"X"** para indicar erros ou estímulos sem resposta e **"?"** para respostas ambíguas ou nos casos em que o aplicador não conseguir anotar as respostas. Essas marcações devem ser feitas sobre ou ao lado de cada nome de emoção na folha de respostas. Se o examinando se autocorrigir, o aplicador deve marcar a última resposta dada. As autocorreções que ocorrem depois não devem ser consideradas.

O que é crucial para a aplicação adequada do teste:

• A tarefa não deve ser administrada antes que o aplicador pratique a marcação das respostas seguindo todas as instruções deste manual até que isso se torne automático.

• O aplicador deve garantir que os examinandos conseguem ver os estímulos e estar atento a possíveis dificuldades de nomeação, percepção e de identificação de expressões de emoção facial.

DETALHES DA ADMINISTRAÇÃO DO TESTE

• O aplicador deve sentar-se ao lado do examinando para que ele(a) também possa ver os estímulos.

• O aplicador deve pedir permissão ao examinando para gravar a sessão em áudio, pois é difícil acompanhar as respostas. Isso permite que o aplicador ouça as respostas novamente caso tenha dificuldade em marcá-las na folha de resposta.

• O aplicador deve pedir ao examinando que leia as instruções ou, se este(a) preferir, as instruções podem ser lidas para ele(a). O aplicador deve garantir que o examinando tenha entendido as instruções antes de iniciar a tarefa. Em caso de dúvida, as instruções devem ser repetidas. O examinando deve estar preparado para começar a nomear emoções o mais rápido possível antes de passar para a página que inclui todos os estímulos.

• O aplicador deve acionar o cronômetro com a mão não dominante assim que o primeiro estímulo estiver visível e pará-lo imediatamente após a resposta referente ao último estímulo em cada bloco. Antes de olhar para o cronômetro, o aplicador deve anotar a resposta referente ao último estímulo. Só então eles(as) devem olhar para o cronômetro e anotar o tempo que o examinando levou para concluir cada bloco (em segundos) na folha de respostas.

• Os examinandos podem descansar entre os blocos. O aplicador deve usar o bom senso para determinar por quanto tempo o examinando pode fazê-lo.

• O examinando deve completar todos os blocos na íntegra. Não há critérios de interrupção para esta tarefa.

COMO CRONOMETRAR QUANTO TEMPO O EXAMINANDO LEVA PARA COMPLETAR CADA BLOCO

• O aplicador deve marcar quanto tempo o examinando leva para concluir cada bloco em segundos, desde o aparecimento do primeiro estímulo até a resposta referente ao último estímulo. Há uma página que precede o início de cada bloco que serve para ajudar a estabelecer quando a tarefa começará. Assim que o examinando classificar o último estímulo, a cronometragem deve ser parada. A resposta ao último estímulo deve ser anotada, primeiro. Somente então o aplicador deve olhar para o cronômetro e marcar o tempo, em segundos, nas células específicas que correspondem a cada bloco.

COMO MARCAR PONTOS

1. Após a conclusão da tarefa, o aplicador deve contar e escrever o número total de respostas corretas nas células correspondentes para cada bloco, em cinza claro à direita na folha de respostas, lembrando que cada resposta correta é equivalente a um ponto. O número cinza claro “(20)” nas células indica o número máximo possível de respostas corretas.

2. O aplicador pode então calcular os custos absolutos da inibição (tempo necessário para concluir o bloco 3 subtraído do tempo necessário para concluir o bloco 1 e/ou 2, ou vice-versa). Faça o mesmo para obter o custo de acurácia. Os custos de inibição relativa podem ser calculados usando divisão em vez de subtração (isto é, desempenho no bloco 3 / bloco 1). Os resultados devem ser anotados na última célula, na parte inferior da folha de respostas (em cinza escuro). Um sistema de pontuação alternativo é o Rate Correct Score, ou o número total de respostas corretas por bloco (ou custo de inibição das respostas corretas) dividido pelo tempo total necessário para concluir o bloco (ou custo de inibição no tempo).

Esteja atento a quaisquer eventos inesperados

• Se houver muitos erros sequenciais, é provável que o examinando tenha omitido ("pulado") um estímulo e/ou que o aplicador não tenha anotado uma ou mais respostas ou autocorreções dos examinandos. A gravação de áudio deve ser verificada.

• Pontuações muito baixas só devem ser interpretadas como dificuldades executivas quando o aplicador acreditar que não há outros déficits perceptivos ou cognitivos que possam explicar os resultados (verifique as características dos examinandos que impedem o uso desta tarefa). Nesses casos, os examinandos devem ser encaminhados ao tipo de profissional que possa confirmar um possível diagnóstico. Se o examinando for menor de idade, o professor e/ou responsáveis devem ser contatados quando houver suspeitas clínicas ou cognitivas que possam justificar o desempenho incomum dos examinandos. Alternativamente, pode ser que o examinando não esteja disposto a executar a tarefa ou seguir as instruções. Use o bom senso para determinar se as pontuações realmente refletem habilidades executivas.

• Use o espaço na parte inferior da folha para registrar incidentes considerados incomuns ou inesperados. Às vezes, apenas em retrospecto é possível entender a importância de eventos que ocorreram durante a avaliação cognitiva.

MATERIAL: 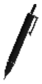 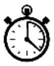 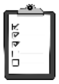 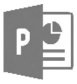 ou 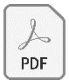

FELIZ TRISTE – FOLHA DE RESPOSTA

**Bloco 1: Emojis**					**Tempo (s)**	**Acertos (nº)**
triste	feliz	triste	feliz	triste		
feliz	triste	triste	feliz	feliz		
triste	feliz	feliz	triste	feliz		
triste	feliz	triste	feliz	triste		(20)

**Bloco 2: Emoções em faces**				**(m: mulher; h: homem)**		
triste (m)	feliz (h)	feliz (m)	triste (h)	triste (m)		
feliz (m)	triste (h)	triste (m)	feliz (m)	feliz (h)		
triste (h)	triste (m)	feliz (h)	triste (h)	feliz (m)		
feliz (h)	feliz (m)	triste (m)	feliz (h)	triste (h)		(20)

**Bloco 3: Emoções opostas em faces**				**(m: mulher; h: homem)**		
feliz (h)	triste (h)	feliz (m)	triste (h)	triste (m)		
triste (m)	feliz (m)	triste (h)	feliz (h)	feliz (m)		
triste (h)	triste (m)	feliz (h)	feliz (m)	triste (h)		
feliz (h)	feliz (m)	triste (m)	feliz (h)	triste (m)		(20)

	**Tempo (s) (3-2)**	**Acertos nº (3-2)**
**Custo de inibição**		

Observações:

FELIZ TRISTE – FOLHA DE RESPOSTA COM EXEMPLOS DE CORREÇÕES

Bloco 1: Emojis					Tempo (s)	Acertos (nº)
triste ✓	feliz ✓	triste ✓	feliz ✓	triste ✓	17	20 (20)
feliz ✓	triste ✓	triste ✓	feliz ✓	feliz ✓		
triste ✓	feliz ✓	feliz ✓	triste ✓	feliz ✓		
triste ✓	feliz ✓	triste ✓	feliz ✓	triste ✓		

Bloco 2: Emoções em faces				(m: mulher; h: homem)	Tempo (s)	Acertos (nº)
triste (m) ✓	feliz (h) ✓	feliz (m) ✓	triste (h) ✓	triste (m) ✓	19	19 (20)
feliz (m) ✓	triste (h) ✓	triste (m) ✓	feliz (m) ✓	feliz (h) ✓		
triste (h) ✗	triste (m) ✓	feliz (h) ✓	triste (h) ✓	feliz (m) ✓		
feliz (h) ✓	feliz (m) ✓	triste (m) ✓	feliz (h) ✓	triste (h) ✓		

Bloco 3: Emoções opostas em faces				(m: mulher; h: homem)	Tempo (s)	Acertos (nº)
feliz (h) ✓	triste (h) ✓	feliz (m) ✓	triste (h) ✓	triste (m) ✓	25	17.89 (20)
triste (m) ?	feliz (m) ✓	triste (h) ✗	feliz (h) ✓	feliz (m) ✓		
triste (h) ✓	triste (m) ✓	feliz (h) ✓	feliz (m) ✗	triste (h) ✓		
feliz (h) ✓	feliz (m) ✓	triste (m) ✓	feliz (h) ✓	triste (m) ✓		

	Tempo (s) (3-2)	Acertos nº (3-2)
Custo de inibição	6	-1.11

Observações:

Detalhes:

- ✓ Respostas corretas.
- ✗ Erro de inibição (por exemplo, nos blocos 1 e 2, dizendo "feliz" para a face triste) ou quando o examinando pular um estímulo.
- ? Dados perdidos devido a respostas ambíguas, falha para escrever a resposta, anotações incompreensíveis, etc. Sugerimos que, se esses casos não excederem 10% dos estímulos em cada bloco, a regra de três * seja usada para estimar o número total de respostas corretas. Quando a perda de dados exceder 10%, os aplicadores devem decidir se vão usar as respostas ou não. Se isso ocorrer no bloco 3, os custos executivos não poderão ser calculados (devem ser considerados valores perdidos).

O exemplo de custo aqui é do bloco 3 menos o bloco 1.

- O custo aqui está exemplificado como custo absoluto do bloco 3 em relação ao bloco 1.

* Regra de três (exemplo para este caso do bloco 3):

17 (respostas corretas) - 19 (número total de itens respondidos - item marcado com ? não é considerado)

x (acertos) - 20 (número total de itens)

x = 17.89 (use dois dígitos após o ponto decimal, arredondando os decimais para os centésimos mais próximos)

TAREFA COR E FORMA – ADMINISTRAÇÃO E PONTUAÇÃO

INSTRUÇÕES GERAIS

Qual domínio executivo é avaliado por esta tarefa: Essa tarefa avalia a capacidade de alternar entre duas tarefas; neste caso em particular, alternar a classificação das figuras (estímulos) de acordo com duas categorias: cor e por forma, seguindo pistas.

O que é requerido que os examinandos façam: Os examinandos devem classificar os estímulos em voz alta por cor, por forma e alternar entre essas classificações de categoria, seguindo pistas colocadas acima de cada estímulo. As tarefas devem ser realizadas o mais rápido possível, evitando erros. Autocorreções são permitidas desde que ocorram antes da resposta referente ao próximo estímulo.

O que a tarefa envolve: Essa tarefa inclui três blocos precedidos por ensaios para treino. Acima de todos os estímulos, há pistas que orientam as classificações (um esboço abstrato em preto ou um arco-íris monocromático). No bloco 1, os examinandos devem classificar cada estímulo por forma (nesse caso a pista é o contorno abstrato em preto); no bloco 2, devem classificar os estímulos por cor (pista: arco-íris monocromático); no bloco 3, os examinandos devem alternar entre as classificações por cor e forma de acordo com as pistas apresentadas sobre os estímulos, que variam de ensaio a ensaio.

As respostas são sempre vocais. A velocidade de realização da tarefa é determinada pelos próprios examinandos. Eles(as) passam das instruções para as tarefas, e entre páginas/slides e tarefas, deslizando o dedo na tela (em casos de telas sensíveis ao toque), pressionando um botão do mouse ou uma tecla no computador ou virando páginas, dependendo do equipamento ou modo de aplicação usado em cada experimento. Os examinandos não devem, no entanto, retornar aos slides/páginas que já foram vistos. O objetivo é concluir cada bloco o mais rápido possível evitando erros.

Quais características do examinando impede o uso desta tarefa para avaliar as funções executivas:

Esta tarefa não deve ser realizada para avaliar o funcionamento executivo de pessoas com deficiência visual ou cuja visão não esteja corrigida, que tenham diagnóstico de distúrbios de linguagem, que não sabe, o nome das cores (preto, cinza) ,formas geométricas (círculos, quadrados) e/ou outras dificuldades que o aplicador julgar poderem interferir no desempenho.

O que o aplicador faz durante a tarefa:

1. Marcação da velocidade de realização da tarefa: O aplicador deve marcar quanto tempo o examinando leva para concluir cada bloco em segundos, desde o aparecimento do primeiro estímulo até a resposta até o último estímulo. Há uma página que precede o início de cada bloco que serve para ajudar a estabelecer quando a tarefa começará. Assim que o examinando classifica o último estímulo, o cronômetro deve ser parado. A resposta ao último estímulo deve ser anotada, primeiro. Somente então o aplicador deve olhar para o cronômetro e marcar o tempo, em segundos, nas células cinza claro na folha de respostas que correspondem a cada bloco.

2. Marcação da acurácia das respostas: Para ajudar o aplicador a acompanhar as respostas, todas as páginas/slides são numeradas e o número é exibido na folha de respostas na extremidade esquerda das células, seguido pela categoria e classificação possíveis dos estímulos: "CR" para círculos, "QD" para quadrados, "PR" para preto ou "CZ" para cinza. No bloco 3, no entanto, próximo ao número do estímulo há dois conjuntos de letras correspondentes às possíveis categorias e classificações (por exemplo, "QD CZ" para o quadrado cinza). Na folha de resposta, o aplicador deve trabalhar de cima para baixo (começando à esquerda) à medida que a tarefa avança. Nesta folha, eles(as) devem usar marcas de tique (✓) para indicar as respostas corretas, **"X"** para indicar erros ou estímulos sem resposta e **"?"** para respostas ambíguas, ou nos casos em que o aplicador não consegue anotar as respostas. Se o examinando se autocorrigir antes de dar a resposta ao estímulo seguinte, o aplicador deve marcar a última resposta fornecida. As autocorreções que ocorrem depois não devem ser consideradas.

No bloco 3, ao aplicar a tarefa, desconsidere as marcações em negrito na letra. Preste atenção apenas à resposta, independentemente da pista. Assim, para um estímulo quadrado cinza ("QD" "**CZ**"), se o examinando disser “cinza”, ele(a) recebe uma marca de **"✓"** (marca de classificação correta) que deve ser colocada acima ou sobre o CZ. Se a resposta for "quadrado", uma marca de **"✓"** deve ser colocada acima ou sobre QD. A

resposta "círculo" deve ser anotada como um **"X"** acima ou sobre o CR e a resposta "preta" como um **"X"** acima ou sobre PR. Neste exemplo, o CZ é marcado em negrito porque é a classificação correta do estímulo seguindo sua pista (arco-íris). No entanto, a capacidade de alternar seguindo a pista é considerada apenas em outra métrica de pontuação (alternâncias corretas; veja abaixo).

O que é crucial para a aplicação adequada do teste:

• A tarefa não deve ser administrada antes que o aplicador pratique a marcação das respostas seguindo todas as instruções deste manual até que isso se torne automático.

• O aplicador deve garantir que os examinandos conseguem ver os estímulos, identificar as cores e formas dos estímulos e estar atento a possíveis dificuldades de nomeação e percepção.

DETALHES DA ADMINISTRAÇÃO DO TESTE

• O aplicador deve sentar-se ao lado do examinando para que ele(a) também possa ver os estímulos.

• O aplicador deve pedir permissão ao examinando para gravar a sessão em áudio, pois é difícil acompanhar as respostas. Isso permite que o aplicador ouça as respostas novamente se tiver dificuldade em marcar respostas na folha de respostas.

• O aplicador deve pedir ao examinando que leia as instruções ou, se este(a) preferir, as instruções podem ser lidas para ele(a). O aplicador deve se certificar de que o examinando entendeu as instruções durante os ensaios para treino. Em caso de dúvida, os ensaios para treinos devem ser repetidos até que o examinando tenha entendido as instruções. Prepare o examinando, no treino, para começar a classificar os estímulos o mais rápido possível antes que eles(as) passem para a página que contém o primeiro estímulo do teste.

• As respostas que o aplicador considera semelhantes às exigidas devem ser contadas como válidas (por exemplo, bola em vez de círculo). Em outras palavras, não se deve insistir que os examinandos usem um termo específico para se referir a um tipo de estímulo, desde que a resposta fornecida seja compreensível.

• O aplicador deve acionar o cronômetro com a mão não dominante assim que o primeiro estímulo estiver visível e pará-lo imediatamente após a resposta referente ao último estímulo em cada bloco. Antes de olhar para o cronômetro, o aplicador deve anotar a resposta referente ao último estímulo. Só então ele(a) deve olhar para o cronômetro e anotar o tempo que o examinando levou para concluir cada bloco (em segundos) na folha de respostas.

• Os examinandos podem descansar entre os blocos. O aplicador deve usar o bom senso para determinar por quanto tempo o examinando pode fazê-lo.

• O examinando deve completar todos os blocos na íntegra. Não há critérios de interrupção para esta tarefa.

COMO CRONOMETRAR QUANTO TEMPO O EXAMINANDO LEVA PARA COMPLETAR CADA BLOCO

• O aplicador deve marcar quanto tempo o examinando leva para concluir cada bloco em segundos, desde o aparecimento do primeiro estímulo até a resposta referente ao último estímulo. Há uma página que precede o início de cada bloco que serve para ajudar a estabelecer quando a tarefa começará. Assim que o examinando classificar o último estímulo, o cronômetro deve ser parado. A resposta ao último estímulo deve ser anotada, primeiro. Somente então o aplicador deve olhar para o cronômetro e marcar o tempo, em segundos, nas células específicas que correspondem a cada bloco.

COMO MARCAR PONTOS

-<u>Blocos 1 e 2:</u>

1. Para os blocos 1 e 2, após a conclusão da tarefa, o aplicador deve contar as marcas de ✓ (respostas corretas) e escrever o número total nas células cinza claro correspondentes e abaixo de cada bloco. Cada resposta correta é igual a um ponto. Não considere ensaios para treino. O número [por exemplo "(20)"] em cinza claro nessas células mostra o número máximo possível de respostas.

- Bloco 3:

1. No bloco 3, após a conclusão da tarefa, o aplicador deve primeiro contar as marcas de ✓ (respostas corretas), independentemente da pista (indicada por letras em negrito). Isso resultará no número de classificações corretas, que devem ser anotadas na célula apropriada na folha de respostas (células cinza claro abaixo do bloco 3). Não considere ensaios para treino.

-Métricas de alternância

1. Calcular as medidas absolutas do custo de alternância: o tempo gasto no bloco de alternância (bloco 3) menos a soma do tempo de blocos completos 1 e 2; escreva esse número nas células cinza escuro correspondentes na parte inferior da folha de respostas. Faça o mesmo para classificações corretas. O custo de alternância relativo também pode ser calculado [por exemplo o tempo gasto no bloco de alternância (bloco 3) dividido pela soma do tempo para completar os blocos 1 e 2]. Um sistema de pontuação alternativo é o Rate Correct Score, ou o número total de respostas corretas por bloco (ou custo de alternância das respostas corretas) dividido pelo tempo total necessário para concluir o bloco (ou custo de alternância no tempo).

2. Somente para o bloco 3, outra métrica de pontuação deve ser usada para considerar se o examinando mudou de categoria (forma ou cor) seguindo as pistas. Isso pode ser feito observando se examinando seguiu as letras em negrito, independentemente das respostas **"✓"** ou **"X"**. Para facilitar a pontuação por esse critério, após a conclusão da tarefa, circule os erros de alternância (quando as respostas não coincidirem com as letras em negrito, independentemente de a classificação estar correta ou não). Em seguida, conte as alternâncias corretas totais no bloco 3 (quantas vezes o examinando seguiu a pista) e adicione o resultado na última célula cinza escuro na folha de respostas.

Esteja atento a quaisquer eventos inesperados

• Se houver muitos erros sequenciais, é provável que o examinando tenha omitido ("pulado") um estímulo e/ou que o aplicador não tenha anotado uma ou algumas respostas ou autocorreções dos examinandos. A gravação de áudio deve ser verificada.

• Pontuações muito baixas só devem ser interpretadas como dificuldades executivas quando o aplicador acreditar que não há outros déficits perceptivos ou cognitivos que possam explicar os resultados (verifique as características dos examinandos que impedem o uso desta tarefa). Nesses casos, os examinandos devem ser encaminhados ao tipo de profissional que possa confirmar um possível diagnóstico. Se o examinando for menor de idade, o professor e/ou responsáveis devem ser contatados quando houver suspeitas clínicas ou cognitivas que possam justificar o desempenho incomum dos examinandos. Alternativamente, pode ser que o examinando não esteja disposto a executar a tarefa ou seguir as instruções. Use o bom senso para determinar se as pontuações realmente refletem habilidades executivas.

• Use o espaço na parte inferior da folha para registrar incidentes considerados incomuns ou inesperados. Às vezes, apenas em retrospecto é possível entender a importância de eventos que ocorreram durante a avaliação cognitiva.

MATERIAL: 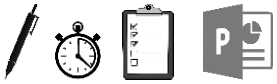 ou 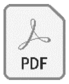

COR E FORMA – FOLHA DE RESPOSTA

TREINO (CIR-círculo; QD-quadrado; PR-preto; CZ-cinza)				
1. Forma		2. Cor		3. Alternância
CIR		PR		**QD** CZ
QD		CZ		CIR **PR**
CIR		PR		QD **CZ**
QD		CZ		**CIR** PR

BLOCO TESTE					
Bloco 1<br>Forma		Bloco 2<br>Cor		Bloco 3<br>Alternância com pista	
01. **QD**		21. **CZ**		41. CIR **PR**	61. CIR **CZ**
02. **CIR**		22. **PR**		42. **QD** CZ	62. **QD** PR
03. **CIR**		23. **PR**		43. **CIR** PR	63. QD **PR**
04. **QD**		24. **CZ**		44. QD **PR**	64. **QD** CZ
05. **QD**		25. **CZ**		45. **CIR** CZ	65. **CIR** PR
06. **CIR**		26. **PR**		46. **QD** PR	66. QD **PR**
07. **QD**		27. **CZ**		47. QD **CZ**	67. QD **CZ**
08. **QD**		28. **PR**		48. **CIR** PR	68. **CIR** PR
09. **CIR**		29. **CZ**		49. CIR **CZ**	69. CIR **CZ**
10. **CIR**		30. **CZ**		50. **QD** PR	70. **CIR** PR
11. **QD**		31. **PR**		51. CIR **PR**	71. CIR **CZ**
12. **QD**		32. **PR**		52. QD **CZ**	72. **QD** CZ
13. **CIR**		33. **CZ**		53. **CIR** CZ	73. QD **PR**
14. **QD**		34. **PR**		54. CIR **PR**	74. CIR **CZ**
15. **CIR**		35. **PR**		55. **QD** CZ	75. **QD** PR
16. **CIR**		36. **CZ**		56. CIR **PR**	76. **QD** CZ
17. **QD**		37. **PR**		57. QD **PR**	77. CIR **CZ**
18. **CIR**		38. **CZ**		58. **CIR** CZ	78. CIR **PR**
19. **QD**		39. **PR**		59. QD **CZ**	79. **CIR** CZ
20. **CIR**		40. **CZ**		60. **QD** PR	80. **CIR** PR

Escore Bloco 1		Escore Bloco 2		Escore Bloco 3	Total escores
Tempo (s):		Tempo (s):		Tempo (s):	Tempo custo [Bloco 3-(1+2)]:
Acertos nº.: (20)		Acertos nº.: (20)		Acertos nº.: (40)	Nº. custo [Bloco 3-(1+2)]:
					Nº. acertos Alternância: (40)

Observações:

COR E FORMA – FOLHA DE RESPOSTA COM EXEMPLOS DE CORREÇÃO

TREINO		(CIR-círculo; QD-quadrado; PR-preto; CZ-cinza)
1. Forma	2. Cor	3. Alternância
CIR ✓	PR ✓	QD ✓ CZ
QD ✓	CZ ✓	CIR PR ✓
CIR ✓	PR ✓	QD CZ ✓
QD ✓	CZ ✓	CIR ✓ PR

BLOCO TESTE			
Bloco 1<br>Forma	Bloco 2<br>Cor	Bloco 3<br>Alternância com pista	
01. QD ✓	21. CZ ✓	41. CIR PR ✗	61. CIR CZ ✓
02. CIR ✓	22. PR ✓	42. QD ✓ CZ	62. QD ✓ PR
03. CIR ✓	23. PR ✓	43. CIR ✓ PR	63. QD PR ✓
04. QD ✓	24. CZ ✓	44. (QD) PR ✓	64. QD ✓ CZ
05. QD ✓	25. CZ ✓	45. CIR (CZ ✓)	65. CIR ✓ PR
06. CIR ✓	26. PR ✓	46. QD ✓ PR	66. QD PR ✓
07. QD ✓	27. CZ ✓	47. QD CZ ✓	67. QD CZ ✓
08. QD ✗	28. PR ✓	48. CIR ✓ PR	68. CIR ✓ PR
09. CIR ✓	29. CZ ✓	49. CIR CZ ✓	69. CIR CZ ✓
10. CIR ✓	30. CZ ✓	50. QD PR ?	70. CIR ✓ PR
11. QD ✓	31. PR ✓	51. CIR PR ✓	71. CIR CZ ✓
12. QD ✓	32. PR ✗	52. QD CZ ✓	72. QD ✓ CZ
13. CIR ✓	33. CZ ✓	53. CIR ✓ CZ	73. (QD ✓) PR
14. QD ✗	34. PR ✓	54. CIR PR ✓	74. CIR CZ ✓
15. CIR ✓	35. PR ✓	55. QD ✓ CZ	75. QD ✓ PR
16. CIR ✓	36. CZ ✓	56. (CIR ✗) PR ✓	76. QD ✓ CZ
17. QD ✓	37. PR ✓	57. QD PR ✓	77. CIR CZ ✓
18. CIR ✓	38. CZ ✗	58. CIR ✓ CZ	78. CIR PR ✓
19. QD ✓	39. PR ✓	59. QD CZ ✓	79. CIR ✓ CZ
20. CIR ✓	40. CZ ✓	60. QD ✓ PR	80. CIR ✓ PR

Escore Bloco 1	Escore Bloco 2	Escore Bloco 3	Total escores
Tempo (s): 23	Tempo (s): 24	Tempo (s): 76	Tempo custo [Bloco 3-(1+2)]: 29
Acertos nº.: 18 (20)	Acertos nº.: 18 (20)	Acertos nº.: 37.95 (40)	Nº. custo [Bloco 3-(1+2)]: 1.95
Observações:			Nº. acertos Alternância: 35.90 (40)

Detalhes:

- ✓ Classificação correta.
- ✗ Erro de classificação (por exemplo, diz "círculo" quando o arco-íris monocromático aparece) ou item pulado.
- ○ Erro de alternância (não seguiu a pista, independentemente de acerto ou erro na classificação).
- ? Dados ausentes devido a respostas ambíguas ou falha na anotação da resposta. Sugerimos que, se esses casos não excederem 10% dos estímulos em cada bloco, a regra de três * seja usada para estimar o número total de respostas. Quando a perda de dados excede 10%, os aplicadores devem decidir se devem usar respostas ou não.

* Regra de três (exemplo para este caso):
37 (respostas corretas) - 39 (total de itens respondidos)
x (acertos) - 40 (número total de itens)
x = 37.95 (use dois dígitos após o ponto decimal, arredondando os decimais para os centésimos mais próximos)

TAREFA ALTERNÂNCIA DE CATEGORIA – ADMINISTRAÇÃO E PONTUAÇÃO

INSTRUÇÕES GERAIS

Qual domínio executivo é avaliado por esta tarefa: Esta tarefa avalia a capacidade de alternar entre duas tarefas; nesse caso em particular, alternar a classificação de figuras (estímulos) de acordo com duas categorias: como entidades vivas / não-vivas (“vivas” ou “mortas”) e por tamanho (“grande” ou “pequeno”), mantendo em mente a ordem das classificações (sem pistas disponíveis) .

O que é requerido que os examinandos façam: Os examinandos precisam classificar os estímulos de imagem, em voz alta, como entidades vivas ou não-vivas ("mortas"), classificá-los por tamanho e alternar sequencialmente entre essas classificações de categoria. As tarefas devem ser realizadas o mais rápido possível, evitando erros. Autocorreções são permitidas desde que ocorram antes da resposta referente ao próximo estímulo.

O que a tarefa envolve: Essa tarefa inclui três blocos precedidos por ensaios para treino. O Bloco 1 requer a classificação de estímulos como entidades vivas e não vivas ("mortas"). O Bloco 2 envolve a classificação de estímulos como maior ou menor (grande ou pequeno) que uma bola de futebol real. No Bloco 3, os examinandos são solicitados a alternar sequencialmente entre as classificações nesses dois tipos de categoria, começando com "vivo ou morto", e devem ter em mente a ordem, pois não há pistas externas sobre qual categorização executar para cada estímulo.

As respostas são sempre vocais. A velocidade de realização da tarefa é determinada pelos próprios examinandos. Eles(as) passam das instruções para as tarefas, e entre páginas/slides e tarefas, deslizando o dedo na tela (em casos de telas sensíveis ao toque), pressionando um botão do mouse ou uma tecla no computador ou virando páginas, dependendo do equipamento ou modo de aplicação usado em cada experimento. Os examinandos não devem, no entanto, retornar aos slides/páginas que já foram vistos. O objetivo é concluir cada bloco o mais rápido possível evitando erros.

Quais características do examinando impede o uso desta tarefa para avaliar as funções executivas:

Esta tarefa não deve ser realizada para avaliar o funcionamento executivo de pessoas com deficiência visual ou cuja visão não esteja corrigida, que tenham diagnóstico de distúrbios de linguagem, que não estão familiarizados com os estímulos e/ou não sabem o tamanho real do que os estímulos representam, tampouco naqueles que não entendem o que significa ser uma entidade viva ou não viva, não sabem o tamanho de uma bola de futebol real ou outras condições que o aplicador julgar poderem interferir no desempenho.

O que o aplicador faz durante a tarefa:

1. Marcação da velocidade de realização da tarefa: O aplicador deve marcar quanto tempo o examinando leva para concluir cada bloco em segundos, desde o aparecimento do primeiro estímulo até a resposta referente ao último estímulo. Há uma página que precede o início de cada bloco que serve para ajudar a estabelecer quando a tarefa começará. Assim que o examinando classifica o último estímulo, o cronômetro deve ser parado. A resposta ao último estímulo deve ser anotada, primeiro. Somente então o aplicador deve olhar para o cronômetro e marcar o tempo em segundos, nas células cinza claro, na folha de respostas que correspondem a cada bloco.

2. Marcação da acurácia das respostas: Para ajudar o aplicador a acompanhar as respostas, todas as páginas/slides são numeradas e o número é exibido na folha de respostas na extremidade esquerda das células, seguido pelo nome do estímulo e sua possível categoria e classificação de estímulos: "M" para morto, "V" para vivo, "G" para grande e "P" para pequeno. No bloco 3, no entanto, ao lado do nome dos estímulos retratados, existem dois conjuntos de letras [por exemplo, para uma figura da aranha, está marcado "VP" que se refere a um ser vivo (V) que é menor (pequeno, P) do que uma bola de futebol).

Na folha de respostas, o aplicador deve trabalhar de cima para baixo (começando à esquerda) à medida que a tarefa avança. Eles(as) devem usar marcas de tique (✓) para indicar as respostas corretas, **"X"** para indicar erros ou estímulos sem resposta e **"?"** para respostas ambíguas, ou nos casos em que o aplicador não conseguir anotar as respostas. Essas marcações devem ser feitas nas letras M, V, G e P, ou acima das figuras, em todos os

blocos. Se o examinando se autocorrigir antes de dar a resposta referente ao estímulo seguinte, o aplicador deve marcar a última resposta fornecida. As autocorreções que ocorrem depois não devem ser consideradas.

No bloco 3, ao anotar as respostas dos examinandos, os aplicadores devem desconsiderar as marcações em negrito na letra que indicam a classificação nas duas categorias de cada estímulo. Eles(as) devem apenas prestar atenção à classificação. Assim, se o examinando classificar uma aranha (**V** P) como viva, uma marca de "✓" (marca de classificação correta) deve ser colocada sobre a letra V. Se a resposta for "pequeno", a marca de "✓"deve ser escrita sobre a letra P. Se o examinando disser "morto", um **"X"** deve ser colocado sobre a letra V e, se a resposta for "grande", o **"X"** deve ser colocado sobre a letra P. Neste exemplo, V é marcado em negrito porque é a classificação correta para esse teste *se o examinando não cometer erros de alternância*. No entanto, a capacidade de alternar categorizações sequencialmente é considerada apenas em outra métrica de pontuação (número correto de alternâncias e classificações corretas quando o examinando muda a categorização desse estímulo específico; veja abaixo).

O que é crucial para a aplicação adequada do teste:

• A tarefa não deve ser administrada antes que o aplicador pratique a marcação das respostas seguindo todas as instruções deste manual até que isso se torne automático.

• O aplicador deve garantir que os examinandos conseguem ver os estímulos, que conhecem o tamanho real do que eles representam, que conseguem classificá-los como entidades vivas e não-vivas e estar atento a possíveis dificuldades de percepção e nomeação.

DETALHES DA ADMINISTRAÇÃO DO TESTE

• O aplicador deve sentar-se ao lado do examinando para que ele(a) também possa ver os estímulos.

• O aplicador deve pedir permissão ao examinando para gravar a sessão em áudio, pois é difícil acompanhar as respostas. Isso permite que o aplicador ouça as respostas novamente caso tenha dificuldade em marcá-las na folha de respostas.

• O aplicador deve pedir ao examinando que leia as instruções ou, se este(a) preferir, as instruções podem ser lidas para ele(a). O aplicador deve se certificar de que o examinando entendeu as instruções durante os ensaios para treino. Em caso de dúvida, os ensaios para treino devem ser repetidos até que o examinando tenha entendido as instruções. Prepare o examinando, no treino, para começar a classificar os estímulos o mais rápido possível antes que eles(as) passem para a página que contém o primeiro estímulo do teste.

• As respostas que o aplicador considera semelhantes às exigidas devem ser contadas como válidas (por exemplo, menor é equivalente a "pequeno"; maior é equivalente a "grande"; não vivo é equivalente a "morto"). Em outras palavras, não se deve insistir que o examinando use um termo específico para se referir a um tipo de estímulo, desde que a resposta fornecida seja compreensível.

• O aplicador deve acionar o cronômetro com a mão não dominante assim que o primeiro estímulo estiver visível e pará-lo imediatamente após a resposta referente ao último estímulo em cada bloco. Antes de olhar para o cronômetro, o aplicador deve anotar a resposta referente ao último estímulo. Só então eles(as) devem olhar para o cronômetro e anotar o tempo que o examinando levou para concluir cada bloco (em segundos) na folha de respostas.

• Os examinandos podem descansar entre os blocos. O aplicador deve usar o bom senso para determinar por quanto tempo o examinando pode fazê-lo.

• O examinando deve completar todos os blocos na íntegra. Não há critérios de interrupção para esta tarefa.

COMO CRONOMETRAR QUANTO TEMPO O EXAMINANDO LEVA PARA COMPLETAR CADA BLOCO

• O aplicador deve marcar quanto tempo o examinando leva para concluir cada bloco em segundos, desde o aparecimento do primeiro estímulo até a resposta referente ao último estímulo. Há uma página que precede o início de cada bloco que serve para ajudar a estabelecer quando a tarefa começará. Assim que o examinando classificar o último estímulo, o cronômetro deve ser parado. A resposta ao último estímulo deve ser anotada, primeiro. Somente então o aplicador deve olhar para o cronômetro e marcar o tempo, em segundos, nas células específicas que correspondem a cada bloco.

COMO MARCAR PONTOS

-Blocos 1 e 2:

1. Para os blocos 1 e 2, após a conclusão da tarefa, o aplicador deve contar as marcas de ✓ (respostas corretas) e escrever o número total na folha de respostas: nas células cinza claro correspondentes e abaixo de cada bloco. Cada resposta correta é igual a um ponto. Não considere respostas de ensaios para treino. O número [por exemplo "(20)"] em cinza claro nessas células mostra o número máximo possível de respostas corretas.

- Bloco 3:

1. No bloco 3, após a conclusão da tarefa, o aplicador deve primeiro contar as marcas de ✓ (respostas corretas), independentemente das letras em negrito. Isso resultará no número de classificações corretas, que devem ser anotadas na célula apropriada na folha de respostas (células cinza claro abaixo do bloco 3). Não considere resposta nos ensaios para treino.

- Métricas de alternância

1. Calcular as medidas absolutas do custo de alternância: determine o tempo gasto no bloco de alternância (bloco 3) menos a soma do tempo de blocos completos 1 e 2; escreva esse número nas células cinza escuro correspondentes na parte inferior da folha de respostas. Faça o mesmo para classificações corretas. O custo de alternância relativo também pode ser calculado [por exemplo o tempo gasto no bloco de alternância (bloco 3) dividido pela soma do tempo para completar os blocos 1 e 2]. Um sistema de pontuação alternativo é o Rate Correct Score, ou o número total de respostas corretas por bloco (ou custo de deslocamento das respostas corretas) dividido pelo tempo total necessário para concluir o bloco (ou custo de deslocamento no tempo).

2. Somente para o bloco 3, outra métrica de pontuação deve ser usada para considerar se o examinando mudou de categorização sequencialmente, independentemente de classificações certas ou erradas (por exemplo, pode ter falado vivo para o estímulo do carro). Para facilitar a pontuação por esse critério, após a conclusão da tarefa, erros de alternância podem ser circulados na folha de respostas. Esses erros ocorrem quando há duas ou mais classificações de estímulos sequenciais usando a mesma categoria (por exemplo, classificando "anel" e "leão" na categoria vivo/morto em uma linha). Esse tipo de erro de alternância pode ser encontrado quando o padrão ziguezague indicado por letras em negrito, de cima para baixo, é quebrado. Depois de contornar os erros de alternância no bloco 3, conte as respostas não circuladas, independentemente de terem sido classificadas corretamente, e adicione o resultado a última célula cinza escura da folha de respostas. Se o examinando não começar com "vivo ou morto", isso também conta como um erro de alternância, portanto o número total de alternâncias corretas é 40 (indicado em cinza claro na célula). Se houver respostas ambíguas ou itens que foram ignorados, a próxima classificação deve ser de uma categoria diferente da resposta não ambígua anterior, mantendo o padrão de alternância.

3. A última métrica é o número de classificações corretas quando a alternância da categorização ocorreu. Para contá-los, destaque as alternâncias não circuladas no bloco 3 e depois conte os que receberam uma marca de ✓. Anote esse número na última célula cinza escura na folha de respostas.

Esteja atento a quaisquer eventos inesperados

• Se houver muitos erros sequenciais, é provável que o examinando tenha omitido (“pulado”) um estímulo e/ou que o aplicador não tenha anotado uma ou algumas respostas ou autocorreções dos examinandos. A gravação de áudio deve ser verificada.

• Pontuações muito baixas só devem ser interpretadas como dificuldades executivas quando o aplicador acreditar que não há outros déficits perceptivos ou cognitivos que possam explicar os resultados (verifique as características dos examinandos que impedem o uso desta tarefa). Nesses casos, os examinandos devem ser encaminhados ao tipo de profissional que possa confirmar um possível diagnóstico. Se o examinando for menor de idade, o professor e/ou responsáveis devem ser contatados quando houver suspeitas clínicas ou cognitivas que possam justificar o desempenho incomum dos examinandos. Alternativamente, pode ser que o examinando não esteja disposto a executar a tarefa ou seguir as instruções. Use o bom senso para determinar se as pontuações realmente refletem habilidades executivas.

• Use o espaço na parte inferior da folha para registrar incidentes considerados incomuns ou inesperados. Às vezes, apenas em retrospecto é possível entender a importância de eventos que ocorreram durante a avaliação cognitiva.

MATERIAL: 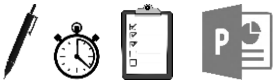 ou 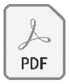

ALTERNÂNCIA DE CATEGORIA – FOLHA DE RESPOSTA

TREINO				(V-vivo; M-morto; P-pequeno; G-grande)
1. Morto/Vivo		2. Grande/Pequeno		3. Alternância
Gorila **V**		Xícara **P**		Aranha **V** P
Xícara **M**		Trem **G**		Xícara M **P**
Trem **M**		Aranha **P**		Gorila **V** G
Aranha **V**		Gorila **G**		Trem M **G**

**BLOCO DE TESTES**											
**Bloco 1 Vivo/Morto**			**Bloco 2 Grande/Pequeno**			**Bloco 3 Alternância (começar com Vivo/Morto)**					
01. Formiga	**V**		21.Carro	**G**		41.Formiga	**V**	P	61.Cavalo	**V**	G
02.Carro	**M**		22.Sapo	**P**		42.Garfo	M	**P**	62.Casa	M	**G**
03.Cavalo	**V**		23.Cama	**G**		43.Carro	**M**	G	63.Joaninha	**V**	P
04.Girafa	**V**		24.Joaninha	**P**		44.Apito	M	**P**	64.Cama	M	**G**
05.Casa	**M**		25.Anel	**P**		45.Elefante	**V**	G	65.Elefante	**V**	G
06.Apito	**M**		26.Garfo	**P**		46.Chave	M	**P**	66.Garfo	M	**P**
07.Garfo	**M**		27.Girafa	**G**		47.Joaninha	**V**	P	67.Chave	**M**	P
08.Leão	**V**		28.Apito	**P**		48.Sapo	V	**P**	68.Borboleta	V	**P**
09.Borboleta	**V**		29.Leão	**G**		49.Cama	**M**	G	69.Leão	**V**	G
10.Sapo	**V**		30.Borboleta	**P**		50.Leão	V	**G**	70.Garfo	M	**P**
11.Cama	**M**		31.Elefante	**G**		51.Elefante	**V**	G	71.Carro	**M**	G
12.Joaninha	**V**		32.Casa	**G**		52.Casa	M	**G**	72.Anel	M	**P**
13.Chave	**M**		33.Formiga	**P**		53.Cavalo	**V**	G	73.Joaninha	**V**	P
14.Anel	**M**		34.Chave	**P**		54.Borboleta	V	**P**	74.Geladeira	M	**G**
15.Geladeira	**M**		35.Cama	**G**		55.Girafa	**V**	G	75.Chave	**M**	P
16.Elefante	**V**		36.Cavalo	**G**		56.Geladeira	M	**G**	76.Sapo	V	**P**
17.Formiga	**V**		37.Borboleta	**P**		57.Sapo	**V**	P	77.Girafa	**V**	G
18.Carro	**M**		38.Girafa	**G**		58.Formiga	V	**P**	78.Apito	M	**P**
19.Apito	**M**		39.Geladeira	**G**		59.Geladeira	**M**	G	79.Casa	**M**	G
20.Leão	**V**		40.Anel	**P**		60.Anel	M	**P**	80.Cavalo	V	**G**

**Escore Bloco 1**		**Escores Bloco 2**		**Escores Bloco 3**	**Total scores**
Tempo (s):		Tempo (s):		Tempo (s):	Tempo custo [3-(1+2)]:
Acertos nºs.: (20)		Acertos nºs.: (20)		Acertos nºs.: (40)	Nº. custo [3-(1+2)]:
				Nº classificações por alternância: (40)	Nº acertos de alternância: (40)

Observações:

ALTERNÂNCIA DE CATEGORIA – FOLHA DE RESPOSTA COM EXEMPLOS DE CORREÇÃO

TREINO		(V-vivo; M-morto; P-pequeno; G-grande)	
1. Morto/Vivo	2. Grande/Pequeno	3. Alternância	
Gorila V ✓	Xícara P ✓	Aranha V✓ P	
Xícara M✓	Trem G ✓	Xícara M P✓	
Trem M✓	Aranha P ✓	Gorila V✓ G	
Aranha V ✓	Gorila G ✓	Trem M G✓	

BLOCO DE TESTES

Bloco 1 Vivo/Morto		Bloco 2 Grande/Pequeno		Bloco 3 Alternância (começar com Vivo/Morto)			
01. Formiga	V✓	21.Carro	G✓	41.Formiga	V✓ P	61.Cavalo	V✓ G
02.Carro	M✓	22.Sapo	P✓	42.Garfo	M P✓	62.Casa	M G✓
03.Cavalo	V ?	23.Cama	G ✗	43.Carro	M✓ G	63.Joaninha	V✓ P
04.Girafa	V✓	24.Joaninha	P ✓	44.Apito	M P✓	64.Cama	M G✓
05.Casa	M✓	25.Anel	P✓	45.Elefante	V✓ G	65.Elefante	V✓ G
06.Apito	M✓	26.Garfo	P✓	46.Chave	M P✓	66.Garfo	M P✓
07.Garfo	M✗	27.Girafa	G✓	47.Joaninha	V P✓ (○)	67.Chave	M✓ P
08.Leão	V✓	28.Apito	P ✗	48.Sapo	V✓ P	68.Borboleta	V P✓
09.Borboleta	V✓	29.Leão	G✓	49.Cama	M G✓	69.Leão	V✓ G
10.Sapo	V✓	30.Borboleta	P ✓	50.Leão	V✓ G	70.Garfo	M P✓
11.Cama	M✓	31.Elefante	G ✓	51.Elefante	V✓ (○) G	71.Carro	M✓ G
12.Joaninha	V✓	32.Casa	G✓	52.Casa	M G✗	72.Anel	M P✗
13.Chave	M✓	33.Formiga	P ✓	53.Cavalo	V✓ G	73.Joaninha	V✓ P
14.Anel	M✓	34.Chave	P✓	54.Borboleta	V P✓	74.Geladeira	M G✓
15.Geladeira	M✓	35.Cama	G✓	55.Girafa	V✓ G	75.Chave	M P✓ (○)
16.Elefante	V✗	36.Cavalo	G✓	56.Geladeira	M G✓	76.Sapo	V P✓ (○)
17.Formiga	V✓	37.Borboleta	P ✓	57.Sapo	V✓ P	77.Girafa	V✓ G
18.Carro	M✓	38.Girafa	G✓	58.Formiga	V P✓	78.Apito	M P✓
19.Apito	M✓	39.Geladeira	G ✓	59.Geladeira	M✓ G	79.Casa	M✓ G
20.Leão	V✗	40.Anel	P✓	60.Anel	M P✓	80.Cavalo	V G✓

Bloco 3 Alternância (começar com Vivo/Morto)			
41 Formiga	[V] P	61 Cavalo	[V] G
42 Garfo	M [P]	62 Casa	M [G]
43 Carro	[M] G	63 Joaninha	[V] P
44 Apito	M [P]	64 Cama	M [G]
45 Elefante	[V] G	65 Elefante	[V] G
46 Chave	M [P]	66 Garfo	M [P]
47 Joaninha	[V] P	67 Chave	[M] P
48 Sapo	V [P]	68 Borboleta	V [P]
49 Cama	[M] G	69 Leão	[V] G
50 Leão	V [G]	70 Garfo	M [P]

Escore Bloco 1	Escores Bloco 2	Escores Bloco 3	Total scores
Tempo (s): 27	Tempo (s): 21	Tempo (s): 59	Tempo custo [3-(1+2)]: 11
Acertos nºs.: 16.84 (20)	Acertos nºs.: 18 (20)	Acertos nºs.: 38 (40)	Nº. custo [3-(1+2)]: 3.16
Observações:		Nº classificações por alternância: 34 (40)	Nº acertos de alternância: 36 (40)

Detalhes:

- ✓ Classificação correta.
- ✗ Erro de classificação (por exemplo, dizendo "vivo" para o desenho que representa a geladeira).
- ○ Erro de alternância (seguindo o mesmo tipo de categorização duas vezes seguidas).
- ? Dados ausentes devido a respostas ambíguas, falha na resposta de gravação, notas incompreensíveis etc. Sugerimos que, se esses casos não excederem 10% dos estímulos em cada bloco, a regra de três * seja usada para estimar o número total de respostas. Quando a perda de dados excede 10%, os aplicadores devem decidir se devem usar respostas ou não.

* Regra de três (exemplo para este caso):
16 (respostas corretas) - 19 (número total de itens respondidos)
x (acertos) - 20 (número total de itens)
x = 16,84 (use dois dígitos após o ponto decimal, arredondando os decimais para os centésimos mais próximos)

TAREFA MEMÓRIA DE NÚMEROS – ADMINISTRAÇÃO E PONTUAÇÃO

INSTRUÇÕES GERAIS

Qual domínio executivo é avaliado por esta tarefa: Esta tarefa avalia a capacidade de atualizar continuamente as informações contidas na memória operacional; nesse caso em particular, manter em mente somente a última sequências de três dígitos mostrados.

O que é requerido que os examinandos façam: O examinando vê uma sequência de dígitos, um de cada vez, e deve se esforçar para lembrar continuamente somente os três últimos dígitos que foram vistos, atualizando essas informações à medida que cada novo dígito é apresentado. A tarefa deve ser realizada o mais rápido possível, evitando erros. Autocorreções são permitidas desde que ocorram antes da resposta referente ao próximo estímulo.

O que a tarefa envolve: Esta tarefa inclui dois blocos de teste com três ensaios cada e é precedida por ensaios para treino. O examinando vê um dígito em cada página. A partir da terceira página, o examinando deve dizer em voz alta os três últimos números (trio) apresentados, na mesma ordem sequencial em que apareceram, incluindo o número que eles(as) veem na página, tendo que atualizar esse trio sequencialmente até a lista de dígitos terminar (indicada por “???” na página). Para essa página, o examinando deve repetir os três últimos números mencionados anteriormente, respeitando a ordem serial. Lembrar esses números indica a memória para trios, sem necessidade de atualização, uma informação que pode ser útil caso se deseje determinar qual o papel da retenção (e não atualização) na memória operacional.

As respostas são sempre vocais. A velocidade de realização da tarefa é determinada pelos próprios examinandos. Eles(as) passam das instruções para a páginas com dígitos, e entre dígitos, ensaios e blocos, deslizando o dedo na tela (em casos de telas sensíveis ao toque), pressionando um botão do mouse ou uma tecla no computador ou virando páginas, dependendo do equipamento ou modo de aplicação usado em cada experimento. Os examinandos não devem, no entanto, retornar aos slides/páginas que já foram vistos. O objetivo é concluir cada bloco o mais rápido possível evitando erros.

Quais características do examinando impedem o uso desta tarefa para avaliar as funções executivas:

Esta tarefa não deve ser realizada para avaliar o funcionamento executivo de pessoas com deficiência visual ou cuja visão não esteja corrigida, que têm distúrbios de linguagem, que não estão familiarizados com os números hindu-arábicos, tampouco naqueles com dificuldade de ordenamento serial e outras dificuldades que o aplicador julgar poderem interferir no desempenho.

O que o aplicador faz durante a tarefa:

1. Marcação da velocidade de realização da tarefa: O aplicador deve marcar quanto tempo o examinando leva para concluir cada ensaio, em segundos, desde o aparecimento do primeiro estímulo até a resposta do último trio (logo antes da página com “???”). O tempo necessário para repetir o último trio não precisa ser cronometrado. Há uma página que precede o início de cada ensaio que serve para ajudar a estabelecer quando a tarefa começará. Assim que o examinando atualiza o último trio, o cronômetro deve ser parado. Anote primeiro a resposta referente ao último estímulo e os dígitos que foram repetidos para verificar a memória após o "???". Somente então o aplicador deve olhar para o cronômetro e marcar o tempo, em segundos, nas células cinza claro na folha de respostas que correspondem a cada ensaio.

2. Marcação da acurácia das respostas: Para ajudar o aplicador a acompanhar as respostas, todos os trios são apresentados na folha de respostas. Cada atualização é indicada como um grupo de 3 dígitos (trio) separados por um espaço do próximo trio. Os primeiros números, em cinza claro, no início de cada sequência na folha de respostas também devem ser ditos em voz alta pelo examinando, mas não são considerados oportunidades de atualização.

Na folha de respostas, o aplicador deve usar marcas de tique (✓) sobre o trio que foi atualizado corretamente (todos os três números devem ser ditos na mesma ordem indicada na folha de respostas). Escreva **"B"** (resposta em branco) acima ou ao lado de qualquer dígito que seja ignorado somente quando o examinando indicar que não se lembra desse dígito específico (considere qualquer expressão usada que torne isso claro). Quando o sujeito

disser menos de três números (por exemplo: “1 2”) e não indicar em qual posição ele(a) esqueceu o número restante desse trio, considere-os como os dois últimos números do trio e marque **"B"** sobre o primeiro. Quando o examinando disser um dígito errado, escreva **"X"** acima do dígito que deveria ter sido dito e depois escreva o dígito errado acima do **"X"**. Esse dígito errado será considerado na próxima oportunidade de atualização; ver abaixo). Usar **"?"** sobre os dígitos do trio para indicar respostas ambíguas ou nos casos em que o aplicador não conseguiu entender a resposta. Se o examinando se autocorrigir antes de dar a resposta ao estímulo seguinte, o aplicador deve marcar a última resposta fornecida. As autocorreções que ocorrem depois não devem ser consideradas.

Para a página/slide contendo “???”, anote a resposta (repetição do último trio) na mesma ordem serial que foi produzida pelo examinando nos três espaços da folha de respostas (“_ _ _”).

O que é crucial para a aplicação adequada do teste:

• A tarefa não deve ser administrada antes que o aplicador pratique a marcação das respostas seguindo todas as instruções deste manual até que isso se torne automático.

• O aplicador deve garantir que os examinandos conseguem ver os estímulos, conhecem os números hindu-arábicos e estar atento a possíveis dificuldades de nomeação, percepção visuoespacial e ordenamento serial.

DETALHES DA ADMINISTRAÇÃO DO TESTE

• O aplicador deve sentar-se ao lado do examinando para que ele(a) também possa ver os estímulos.

• O aplicador deve pedir permissão ao examinando para gravar a sessão em áudio, pois é difícil acompanhar as respostas. Isso permite que o aplicador ouça as respostas novamente caso tenha dificuldade em marcar as respostas na folha de respostas.

• O aplicador deve pedir ao examinando que leia as instruções ou, se este(a) preferir, as instruções podem ser lidas para ele(a). O aplicador deve se certificar de que o examinando entendeu as instruções durante os ensaios para treino. Em caso de dúvida, os ensaios para treino devem ser repetidos até que o examinando tenha entendido as instruções. Prepare o examinando para começar a nomear números o mais rápido possível antes de passarem para a página que inclui o primeiro estímulo.

• O aplicador deve acionar o cronômetro com a mão não dominante assim que o primeiro estímulo estiver visível e pará-lo imediatamente após a resposta referente ao último estímulo em cada ensaio. Antes de olhar para o cronômetro, o aplicador deve anotar a resposta referente ao último estímulo. Só então ele(a) deve olhar para o cronômetro e anotar o tempo que o examinando levou para concluir cada bloco (em segundos) na folha de respostas.

• Os examinandos podem descansar entre os blocos. O aplicador deve usar o bom senso para determinar por quanto tempo o examinando pode fazê-lo.

• O examinando deve completar todos os blocos na íntegra. Não há critérios de interrupção para esta tarefa.

COMO CRONOMETRAR QUANTO TEMPO O EXAMINANDO LEVA PARA COMPLETAR CADA ENSAIO

• O aplicador deve marcar quanto tempo o examinando leva para concluir cada ensaio, em segundos, desde o aparecimento do primeiro estímulo até a resposta do último trio a ser atualizado, quando o cronômetro deve ser parado. Há uma página que precede o início de cada ensaio que serve para ajudar a estabelecer quando a tarefa começará. Ao final de cada ensaio o examinando deve repetir o último trio, a ser anotado nos espaços _ _ _ na folha de respostas, na mesma ordem em que foi falado pelo examinando. Somente então o aplicador deve olhar para o cronômetro e marcar o tempo, em segundos, nas células cinza claro na folha de respostas que correspondem a cada ensaio.

COMO MARCAR PONTOS

1. Após o término da tarefa, o aplicador deve contar o número de marcas de seleção que indicam trios atualizados corretamente, respeitando a ordem serial dentro de cada trio. Cada trio atualizado corretamente corresponde a um ponto. O número total de trios atualizados corretamente em cada avaliação deve ser anotado na coluna cinza claro à direita de cada ensaio. Isso deve ser feito considerando o que segue (itens a-d abaixo):

a. A repetição vocal dos dígitos (impressos) em cinza claro no início de cada avaliação não é contabilizada como atualização (as atualizações são consideradas apenas a partir do quarto dígito em cada lista). Da mesma forma, não considere o desempenho nos ensaios para treino.

b. Inversão das posições seriais dos números dentro de um trio ou dizer apenas um ou dois dos três dígitos de um trio corretamente, mesmo se nas posições seriais corretas, não conta como uma atualização correta. Nesses casos, o examinando recebe zero ponto para esse trio em particular.

c. Se o examinando cometer um erro e incluir um dígito que não esteja no trio, o próximo trio será considerado correto se a atualização refletir o erro anterior. Por exemplo, se o trio foi 123 e o examinando diz 132, isso conta como um erro de atualização. No entanto, se 4 for o próximo número a aparecer e o examinando atualizar o trio como 324, esta é uma resposta correta (atualização correta) porque o examinando fez a atualização com base no que foi mantido em sua memória operacional, mesmo que um erro tenha sido cometido no trio anterior. Considere o mesmo para respostas em branco. É por isso que os dígitos ignorados ou indicados pelos examinandos como "em branco" (B) e o dígito recuperado incorretamente devem ser escritos acima ou ao lado do número correto em cada trio da folha de respostas.

d. Os números em cinza claro [por exemplo, “(2)”] no canto inferior direito das células correspondem ao número máximo de atualizações (ou possíveis dígitos a serem repetidos a partir do último trio) nos ensaios correspondentes nessas células.

2. Na última coluna à direita, anote o número total de dígitos do último trio, em cada ensaio, que foi recuperada da memória (máximo de 3), respeitando a ordem serial. Por exemplo, se o trio foi 123 e o examinando disser 321, a pontuação será 1 (apenas o dígito 2 estava na posição serial correta).

3. Some o tempo gasto e a acurácia (trios atualizados e recuperados corretamente) em todas os ensaios de teste (não leve em consideração os ensaios para treino) e escreva os resultados nas células cinza escuro correspondentes na parte inferior da folha de respostas.

Esteja atento a quaisquer eventos inesperados

• Se houver muitos erros sequenciais, é provável que o examinando tenha omitido (“pulado”) um estímulo e/ou que o aplicador não tenha anotado uma ou algumas respostas ou autocorreções dos examinandos. A gravação de áudio deve ser verificada.

• Pontuações muito baixas só devem ser interpretadas como dificuldades executivas quando o aplicador acreditar que não há outros déficits perceptivos ou cognitivos que possam explicar os resultados (verifique as características dos examinandos que impedem o uso desta tarefa). Nesses casos, os examinandos devem ser encaminhados ao tipo de profissional que possa confirmar um possível diagnóstico. Se o examinando for menor de idade, o professor e/ou responsáveis devem ser contatados quando houver suspeitas clínicas ou cognitivas que possam justificar o desempenho incomum dos examinandos. Alternativamente, pode ser que o examinando não esteja disposto a executar a tarefa ou seguir as instruções. Use o bom senso para determinar se as pontuações realmente refletem habilidades executivas.

• Use o espaço na parte inferior da folha para registrar incidentes considerados incomuns ou inesperados. Às vezes, apenas em retrospecto é possível entender a importância de eventos que ocorreram durante a avaliação cognitiva.

MATERIAL: ou

MEMÓRIA DE NÚMEROS – FOLHA DE CORREÇÃO

LISTA (cada trio corresponde a uma atualização)<br><br>TREINO	Tempo(s)	Nº acertos atualizações (max. 2,4 ou 6)	Nº acertos nos últimos três números (max.3)
**Treino 1**			
1 12 123 234 345 456 **567** __ __ __		(4)	
5 52 526 264 641 417 178 789 **893** __ __ __		(6)	
**Treino 2**			
3 31 312 124 248 487 **876** __ __ __		(4)	
8 89 893 935 **354** __ __ __		(2)	
3 36 367 674 742 429 291 915 **158** __ __ __		(6)	

BLOCOS DE TESTES

**Bloco de Teste 1**			
6 65 653 531 **315** __ __ __		(2)	
7 76 762 625 259 598 985 857 **574** __ __ __		(6)	
3 31 314 143 438 386 **869** __ __ __		(4)	

**Bloco de Teste 2**			
2 27 275 752 524 247 **473** __ __ __		(4)	
5 52 526 264 641 417 173 736 **361** __ __ __		(6)	
8 81 817 173 **736** __ __ __		(2)	

**Total**		(24)	(18)

Observações:

MEMÓRIA DE NÚMEROS – FOLHA DE RESPOSTA COM EXEMPLOS DE CORREÇÃO

LISTA (cada trio corresponde a uma atualização)<br>TREINO PRÁTICO	Tempo(s)	Nº acertos atualizações (max. 2,4 or 6)	Nº acertos nos últimos três números (max.3)
**Treino 1**			
1 12 123 234 345 456 **567** 5 6 7	16	4 (4)	3
5 52 526 264 641 417 178 789 **893** 8 9 3	40	6 (6)	3
**Treino 2**			
3 31 312 124 248 487 **876** 7 8 7 6	25	3 (4)	3
8 89 893 935 **354** 9 2 7	16	2 (2)	0
3 36 367 674 742 429 291 915 **158** B 5 8	44	6 (6)	2

BLOCOS DE TESTES

Bloco de Teste 1			
6 65 653 **531 315** 3 1 5	18	2 (2)	3
7 76 762 **625 259 598 985 857 574** 28 2 8 4	38	5 (6)	3
3 31 314 **143 438 386 869** ? 8 6 9	24	3.64 (4)	3

Bloco de Teste 2			
2 27 275 **752 524 247 473** 8 8 4 5 3	24	3 (4)	2
5 52 526 **264 641 417 173 736 361** 3 6 1	38	6 (6)	3
8 81 817 **173 736** BB 9 7 3 6	16	0 (2)	3
**Total**	158	19.64 (24)	17 (18)

Observação:

Detalhes:

- ✓ Atualização correta de um trio inteiro (números em cinza claro no início de cada lista não recebem pontos).
- ✗ Erro de atualização (por exemplo, dizer um número que não faz parte do trio) ou inverter a ordem serial dos itens no trio ou ignorar um ou mais dígitos do trio (ver exemplos na tabela a seguir).
- ***B*** (Branco) quando examinando diz que não se lembra de um número específico de um trio, indicando sua posição serial no trio. Quando relatar menos de três dígitos para um trio, a marca **B** deve ser feita no primeiro dígito do trio. Conte todos os trios com **B** como erros.

- **?** Dados perdidos devido a respostas ambíguas, ou falha para escrever a resposta ou anotações incompreensíveis. Sugerimos que, se esses casos não excederem 10% dos estímulos em cada bloco, a regra de três * seja usada para estimar o número total de respostas corretas. Primeiro * use uma regra de três considerando o total de 12 possíveis atualizações em cada bloco. Em seguida, use outra regra de três ** para determinar a proporção de atualizações corretas no teste em que respostas **"?"** Ocorreram. Se os dados perdidos excederem 10%, o aplicador deve decidir como proceder.

* Regra de três (exemplo considerando bloco 1, com uma resposta **?**):
10 (respostas corretas) – 11 (nº total de atualizações excluindo dados perdidos)
x (acertos) – 12 (nº total de atualizações)
x = 10.91 (use duas casas após a vírgula, arredondando a última decimal para números inteiros)

** Em seguida, calcule a equivalência no teste em que houve uma resposta **"?"** (No exemplo, com 4 atualizações):
10.91 (respostas corretas) – 12 (nª total)
x (acertos) – 4 (nª atualizações em ensaios com **?**)
x = 3.64 (arredonde decimais para números inteiros, para a casa decimal mais próxima)

Exemplo de como pontuar erros:

- O examinando não acerta o trio (por exemplo, em vez de dizer "124", ele(a) diz "724"). Importante: o trio que foi dito deve ser considerado na próxima tentativa de atualização. Em outras palavras, o examinando deve atualizar o próximo trio considerando os três últimos números que ele(a) lembrou no trio anterior.

- O examinando não conseguiu lembrar os três últimos números (em vez de dizer "354" ele(a) disse "927"). Considere um erro de memória ou zero pontos na última coluna.

- O examinando erra o primeiro e o segundo número do último trio, então não ganha um ponto para essa atualização. Em seguida, o examinando deve repetir o último trio da memória, então para esta repetição o que conta é o trio que ele(a) disse anteriormente (284) e não o trio original (574). Nesse caso, porque na repetição o examinando diz 284, repetindo o trio inteiro corretamente em função do trio que reteve na memória, mesmo que errado ele(a) ganha 3 pontos para "memória".

- O examinando erra um número (por exemplo, ao invés de dizer "524" ele(a) diz "584"), mas o trio a seguir é atualizado corretamente, mantendo o número informado anteriormente, mesmo que erroneamente: considere um erro para o primeiro trio e acerto para o segundo.

- O examinando não se lembra dos dois primeiros dígitos (1 e 7) do primeiro trio e indica isso dizendo algo na linha "em branco". Ele também errou o primeiro número do próximo trio [em vez de "7" ele(a) diz "9"]. Considere esses dois casos como erros de atualização.

TAREFA DOIS PARA TRÁS (2-Back) – ADMINISTRAÇÃO E PONTUAÇAO

INSTRUÇÕES GERAIS

Qual domínio executivo é avaliado por esta tarefa: Esta tarefa avalia a capacidade de atualizar o conteúdo retido na memória operacional; nesse caso em particular, reter a localização espacial dos dois últimos estímulos alvo apresentados e compará-lo à localização do estímulo alvo visível na página/slide.

O que é requerido que os examinandos façam: Nesta tarefa, existem blocos de teste precedidos por ensaios para treino. O examinando deve acompanhar a localização espacial dos quadrados pretos que mudam de posição de página para página e responder se a localização na página que vê é igual ou diferente da localização do quadrado preto duas páginas atrás. As tarefas devem ser realizadas o mais rápido possível, evitando erros. Autocorreções são permitidas desde que ocorram antes da resposta referente ao próximo estímulo.

O que a tarefa envolve: Essa tarefa inclui três blocos que envolvem a mesma tarefa e é precedida por vários ensaios para treino. Em todos os blocos os examinandos devem dizer em voz alta se o quadrado preto que veem na página está na mesma posição (resposta verbal equivalente a “=”) ou em uma posição diferente (resposta verbal equivalente a “≠”) que o quadrado preto que apareceu duas páginas atrás. Note que nenhuma resposta é necessária nas duas primeiras páginas/slides em todos os blocos (NRN = nenhuma resposta é necessária).

As respostas são sempre vocais. A velocidade de realização da tarefa é determinada pelos próprios examinandos. Eles(as) passam das instruções para a tarefa, e entre páginas/slides e blocos, deslizando o dedo na tela (em casos de telas sensíveis ao toque), pressionando um botão do mouse ou uma tecla no computador ou virando páginas, dependendo do equipamento ou modo de aplicação usado em cada experimento. Os examinandos não devem, no entanto, retornar aos slides/páginas que já foram vistos. O objetivo é concluir cada bloco o mais rápido possível evitando erros.

Quais características do examinando impedem o uso desta tarefa para avaliar as funções executivas:

Esta tarefa não deve ser realizada para avaliar o funcionamento executivo de pessoas com deficiência visual ou cuja visão não esteja corrigida, que tenham diagnóstico de distúrbios de linguagem, dificuldade em ordenamento serial e percepção visuoespacial ou outras dificuldades que o aplicador julgar poderem interferir no desempenho

O que o aplicador faz durante a tarefa:

1. Marcação da velocidade de realização da tarefa: O aplicador deve marcar quanto tempo o examinando leva para concluir cada bloco em segundos, desde o aparecimento do primeiro estímulo até a resposta até o último estímulo. Há uma página que precede o início de cada bloco que serve para ajudar a estabelecer quando a tarefa começará. Assim que o examinando responder “igual” ou “diferente” ao último estímulo, o cronômetro deve ser parado. A resposta ao último estímulo deve ser anotada, primeiro. Somente então o aplicador deve olhar para o cronômetro e marcar o tempo em segundos nas células cinza claro na folha de respostas que correspondem a cada bloco.

2. Marcação da acurácia das respostas: Para ajudar o aplicador a acompanhar as respostas, as páginas/slides são numeradas no canto inferior direito das páginas e na folha resposta. Na folha de respostas, o aplicador deve usar marcas de tiques (✓) para indicar as respostas corretas, **"X"** para indicar erros ou estímulos sem resposta e **"?"** para respostas ambíguas, ou nos casos em que o aplicador não conseguir anotar as respostas**. "B"** [marca indicativa da falta de resposta para um estímulo particulares (branco; considere qualquer expressão usada que deixe isso claro)] deve ser usada somente quando o examinando explicita ter esquecido a localização de dois quadrados atrás (conte como erro). Essas marcações **"B"** devem ser feitas sobre ou acima dos símbolos = ou ≠. Se o examinando se autocorrigir antes de dar a resposta ao estímulo seguinte, o aplicador deve marcar a última resposta fornecida. As autocorreções que ocorrem depois não devem ser consideradas.

O que é crucial para a aplicação adequada do teste:

• A tarefa não deve ser administrada antes que o aplicador pratique a marcação das respostas seguindo todas as instruções deste manual até que isso se torne automático.

• O aplicador deve garantir que os examinandos conseguem ver os estímulos e estar atento a possíveis dificuldades de percepção visuoespacial, nomeação ou de ordenamento serial de informações.

DETALHES DA ADMINISTRAÇÃO DO TESTE

• O aplicador deve sentar-se ao lado do examinando para que ele(a) também possa ver os estímulos.

• O aplicador deve pedir permissão ao examinando para gravar a sessão em áudio, pois é difícil acompanhar as respostas. Isso permite que o aplicador ouça as respostas novamente se tiver dificuldade em marcar respostas na folha de respostas.

• O aplicador deve pedir ao examinando que leia as instruções ou, se este(a) preferir, as instruções podem ser lidas para ele(a). O aplicador deve se certificar de que o examinando entendeu as instruções durante os ensaios para treino. Em caso de dúvida, os ensaios para treino devem ser repetidos até que o examinando tenha entendido as instruções. Prepare os examinandos para prestar atenção à localização espacial do quadrado alvo antes de passarem para a página que contém o primeiro estímulo.

• O aplicador deve iniciar o cronômetro com a mão não dominante assim que o primeiro estímulo estiver visível e pará-lo imediatamente após a última resposta ter sido dada em cada ensaio. Antes de olhar para o cronômetro, o aplicador deve anotar a resposta referente ao último estímulo. Só então deve olhar para o cronômetro e anotar o tempo que o examinando levou para concluir cada teste (em segundos) na folha de respostas.

• Os examinandos podem descansar entre os blocos. O aplicador deve usar o bom senso para determinar por quanto tempo o examinando pode fazê-lo.

• O examinando deve completar todos os blocos na íntegra. Não há critérios de interrupção para esta tarefa.

COMO CRONOMETRAR QUANTO TEMPO O EXAMINANDO LEVA PARA COMPLETAR CADA BLOCO

• O aplicador deve marcar quanto tempo o examinando leva para concluir cada bloco em segundos, desde o aparecimento do primeiro estímulo até a resposta referente ao último estímulo. Há uma página que precede o início de cada bloco que serve para ajudar a estabelecer quando a tarefa começará. Assim que o examinando classificar o último estímulo, o cronômetro deve ser parado. A resposta ao último estímulo deve ser anotada, primeiro. Somente então o aplicador deve olhar para o cronômetro e marcar o tempo, em segundos, nas células específicas que correspondem a cada bloco.

COMO MARCAR PONTOS

1. Após a conclusão da tarefa, o aplicador deve anotar o número total de respostas corretas nas células à direita de cada bloco, lembrando que cada resposta correta equivale a um ponto. O número cinza claro [por exemplo "(22)"] nas células indica o número máximo possível de respostas corretas que podem ser colocadas nessa célula.

2. O aplicador deve somar o tempo e as respostas corretas nos três blocos (excluindo escores de treino) e escrever o resultado na célula apropriada em cinza escuro na parte inferior da folha de respostas.

Esteja atento a quaisquer eventos inesperados

• Se houver muitos erros sequenciais, é provável que o examinando tenha omitido (“pulou”) um estímulo e/ou que o aplicador não tenha anotado uma ou algumas respostas ou autocorreções dos examinandos. A gravação de áudio deve ser verificada.

• Pontuações muito baixas só devem ser interpretadas como dificuldades executivas quando o aplicador acreditar que não há outros déficits perceptivos ou cognitivos que possam explicar os resultados (verifique as características dos examinandos que impedem o uso desta tarefa). Nesses casos, os examinandos devem ser encaminhados ao tipo de profissional que possa confirmar um possível diagnóstico. Se o examinando for menor de idade, o professor e/ou responsáveis devem ser contatados quando houver suspeitas clínicas ou cognitivas que possam justificar o desempenho incomum dos examinandos. Alternativamente, pode ser que o examinando não esteja disposto a executar a tarefa ou seguir as instruções. Use o bom senso para determinar se as pontuações realmente refletem habilidades executivas.

• Use o espaço na parte inferior da folha para registrar incidentes considerados incomuns ou inesperados. Às vezes, apenas em retrospecto é possível entender a importância de eventos que ocorreram durante a avaliação cognitiva.

MATERIAL: 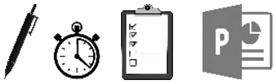 ou 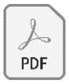

TWO-BACK – FOLHA DE RESPOSTA

TREINO PRÁTICO

Treino 1												Tempo (s)	Acertos (nºs)
1) NRN	2) NRN	3) =	4) ≠	5) ≠	6) ≠	7) =	8) ≠	9) ≠	10) ≠	11) ≠	12) =		
**Treino 2**													
13) NRN	14) NRN	15) ≠	16) =	17) ≠	18) ≠	19) ≠	20) =	21) ≠	22) ≠	23) =	24) ≠		
25) ≠	26) ≠	27) =	28) ≠	29) ≠	30) =	31) ≠	32) ≠	33) ≠	34) =	35) ≠	36) ≠		(22)

BLOCOS DE TESTE

Bloco 1													
1) NRN	2) NRN	3) ≠	4) ≠	5) =	6) ≠	7) ≠	8) =	9) ≠	10) =	11) ≠	12) ≠		
13) ≠	14) =	15) ≠	16) ≠	17) ≠	18) ≠	19) =	20) ≠	21) ≠	22) ≠	23) =	24) ≠		(22)

Bloco 2													
25) NRN	26) NRN	27) =	28) =	29) ≠	30) ≠	31) ≠	32) ≠	33) ≠	34) ≠	35) =	36) ≠		
37) ≠	38) ≠	39) =	40) ≠	41) ≠	42) ≠	43) ≠	44) =	45) ≠	46) ≠	47) ≠	48) =		(22)

Bloco 3													
49) NRN	50) NRN	51) ≠	52) =	53) ≠	54) ≠	55) =	56) ≠	57) ≠	58) ≠	59) ≠	60) ≠		
61) =	62) ≠	63) ≠	64) =	65) ≠	66) ≠	67) ≠	68) =	69) ≠	70) ≠	71) =	72) ≠		(22)

	Tempo (s)	Acertos (nºs)
**Total**		(66)

NRN= nenhuma resposta é necessária.
Observações:

TWO-BACK – FOLHA DE RESPOSTA COM EXEMPLOS DE CORREÇÃO

TREINO PRÁTICO

Treino 1											
1) NRN	2) NRN	3) = ✓	4) ≠ ✓	5) ≠ ✓	6) ≠ ✓	7) = ✓	8) ≠ ✓	9) ≠ ✓	10) ≠ ✓	11) ≠ ✓	12) = ✓

Treino 2												Tempo (s)	Acertos (nºs)
13) NRN	14) NRN	15) ≠ ✓	16) = ✗	17) ≠ ✓	18) ≠ ✓	19) ≠ ✓	20) = ✓	21) ≠ ✓	22) ≠ ✓	23) = ✓	24) ≠ ✓	80	21
25) ≠ ✓	26) ≠ ✓	27) = ✓	28) ≠ ✓	29) ≠ ✓	30) = ✓	31) ≠ ✓	32) ≠ ✓	33) ≠ ✓	34) = ✓	35) ≠ ✓	36) ≠ ✓		(22)

BLOCOS DE TESTE

Bloco 1													
1) NRN	2) NRN	3) ≠ ✓	4) ≠ ✓	5) = ✓	6) ≠ ✓	7) ≠ ✓	8) ✗	9) ≠ ✓	10) = ✓	11) ≠ ✓	12) ≠ ✓	71	19
13) ≠ ✓	14) = ✓	15) ≠ ✓	16) ≠ ✗	17) ≠ ✓	18) ≠ ✓	19) = ✓	20) ≠ ✓	21) ≠ ✓	22) ≠ ✓	23) = ✗	24) ≠ ✓		(22)

Bloco 2													
25) NRN	26) NRN	27) = ✓	28) = ✓	29) ≠ ✓	30) ≠ ✓	31) ≠ B	32) ≠ ✓	33) ≠ ✓	34) ≠ ✓	35) = ✗	36) ≠ ✓	69	19
37) ≠ ✓	38) ≠ ✓	39) = ✗	40) ≠ ✓	41) ≠ ✓	42) ≠ ✓	43) ≠ ✓	44) = ✓	45) ≠ ✓	46) ≠ ✓	47) ≠ ✓	48) = ✓		(22)

Bloco 3													
49) NRN	50) NRN	51) ≠ ✓	52) = ✗	53) ≠ ✓	54) ≠ ✓	55) = ?	56) ≠ ✓	57) ≠ ✓	58) ≠ ✓	59) ≠ ✓	60) ≠ ✓	68	19.90
61) = B	62) ≠ ✓	63) ≠ ✓	64) = ✓	65) ≠ ✓	66) ≠ ✓	67) ≠ ✓	68) = ✓	69) ≠ ✓	70) ≠ ✓	71) = ✓	72) ≠ ✓		(22)

	Tempo (s)	Acertos (nºs)
Total	208	57.90 (66)

NRN= nenhuma resposta é necessária.
Observações:

Detalhes:

- ✓ Resposta correta.
- ✗ Erro de atualização (por exemplo, diz "igual" quando o correto é "diferente") ou estímulo ignorado.
- B (Branco) quando examinando diz que não se lembra do local de dois quadrados atrás. Contar como erro.
- ? Dados perdidos devido a respostas ambíguas, falha para escrever a resposta ou anotações incompreensíveis. Sugerimos que, se esses casos não excederem 10% dos estímulos em cada bloco, a regra de três * seja usada para estimar o número total de respostas corretas. Se os dados perdidos excederem 10%, o aplicador deve decidir como proceder.

* Regra de três (exemplo para esse caso):
19 (respostas corretas) – 21 (nº total de itens respondidos + resposta em branco, indicadas explicitamente)
x (acertos) – 22 (nº total de itens)
x=19.90 (use dois dígitos após o ponto decimal, arredondando os decimais para os centésimos mais próximos)
